# Phenotyping the genus *Hypericum* by secondary metabolite profiling: emodin vs. skyrin, two possible key intermediates in hypericin biosynthesis

**DOI:** 10.1007/s00216-018-1384-0

**Published:** 2018-10-05

**Authors:** Katarína Kimáková, Andrea Kimáková, Jakub Idkowiak, Maciej Stobiecki, Paweł Rodziewicz, Łukasz Marczak, Eva Čellárová

**Affiliations:** 1 Faculty of Science, Institute of Biology and Ecology, Department of Genetics, P. J. Šafárik University in Košice, Mánesova 23, 040 01 Košice, Slovakia; 2 Institute of Bioorganic Chemistry, Polish Academy of Sciences, Noskowskiego 12/14, 61-704 Poznań, Poland

**Keywords:** Anthraquinones, *Hypericum*, Hypericin biosynthesis, Metabolite profiling, Naphthodianthrones, Phloroglucinols, Liquid chromatography mass spectrometry

## Abstract

A wide range of compounds that occur in the genus *Hypericum* are listed as effective drugs of natural origin. The main biological activities of several *Hypericum* representatives are due to the presence of naphthodianthrones, phloroglucinols, and other diverse groups of secondary metabolites that synergistically contribute to their therapeutic effects. The regulation of biosynthesis of hypericin as the key bioactive naphthodianthrone remains uncertain. Here, we present liquid chromatography mass spectrometry-based phenotyping of 17 *Hypericum* species, the results of which suggest an important role for skyrin and its derivatives in the polyketide pathway that leads to hypericin formation. Moreover, we report for the first time the presence of new metabolites in the genus *Hypericum* that are related to classes of anthraquinones, their derivatives, and phloroglucinols. As skyrin and other species of anthraquinones are rarely found in higher plants but frequently occur in fungal microorganisms, the obtained results suggest that further research on the synthesis pathways of hypericin and the role of anthraquinone derivatives in plant metabolism should be carried out. The fact that these compounds are commonly synthesized in endophytic fungi and perhaps there is some similarity in the metabolic pathways between these organisms should also be investigated.

**Electronic supplementary material:**

The online version of this article (10.1007/s00216-018-1384-0) contains supplementary material, which is available to authorized users.

## Introduction

The genus *Hypericum* contains nearly 500 representatives [1] of which *H. perforatum* is the most studied and well-characterized species. The presence of a complex spectrum of bioactive compounds ranks this species as one of the top herbal remedies and supplements in the world according to Global Industry Analysts, Inc. [2]. However, very few metabolic profiles of other *Hypericum* species are available in the phytochemical interaction database (PCIDB, http://www.genome.jp/db/pcidb). A comprehensive review by Stojanovič et al. [3] concentrated on the presence of 11 main bioactive compounds including naphthodianthrones, phloroglucinols, flavonoids, and xanthones in 132 representatives of the *Hypericum* genus. Porzel et al. [4] compared the use of two methods, liquid chromatography mass spectrometry (LC-MS) and nuclear magnetic resonance (NMR) spectroscopy coupled with multivariate data analysis, for profiling the floral metabolome of six *Hypericum* spp.; they found 38 compounds, and some of them remain unidentified or even unknown. Despite the wide use of *Hypericum* preparations and the promising potential of hypericin in the diagnosis and treatment of cancer [5], the *in planta* biosynthesis of some secondary metabolites, especially naphthodianthrones, is not well understood. Hypericin is synthesized via the polyketide pathway. The results of Kusari et al. [6] and Nigutová et al. [7] revealed a positive correlation between the accumulation of hypericin/pseudohypericin and emodin, which led to the conclusion that the anthraquinone emodin could be a putative precursor of hypericin. However, the occurrence of emodin in higher plants is not restricted to hypericin-producing *Hypericum* spp. [8]. In the plant kingdom, hypericin is present almost exclusively in some species of the genus *Hypericum*, but it can be found in several fungi [9], where it is accompanied by a bisanthraquinone, skyrin. Skyrin was detected in *H. perforatum* [10] and proposed as a precursor of protohypericin [11]. Except for the isolation of a few glycosides of skyrin in some *Hypericum* spp. [12–14], its potential role as an intermediate of hypericin biosynthesis has not been further investigated.

To better understand the biosynthetic sites of its main bioactive compounds, we recently developed an approach for the accurate localization of 17 *Hypericum* spp. using desorption electrospray ionization mass spectrometric imaging (DESI-MSI) [15], matrix-assisted laser desorption/ionization high-resolution mass spectrometry (MALDI-HRMS) [16], and metabolic profiling coupled to robust chemometric analyses [7]. Consequently, the aim of the present study was to perform a comprehensive metabolomic analysis of selected *Hypericum* spp. to investigate a wide spectrum of bioactive compounds, to use the dataset to explore the relatedness between groups of secondary metabolites through principal component analysis (PCA) to reveal potential intermediate(s) in hypericin biosynthesis, and to ascertain the presence of compounds not yet identified in the genus *Hypericum* thus far.

## Results

**Scheme 1:**
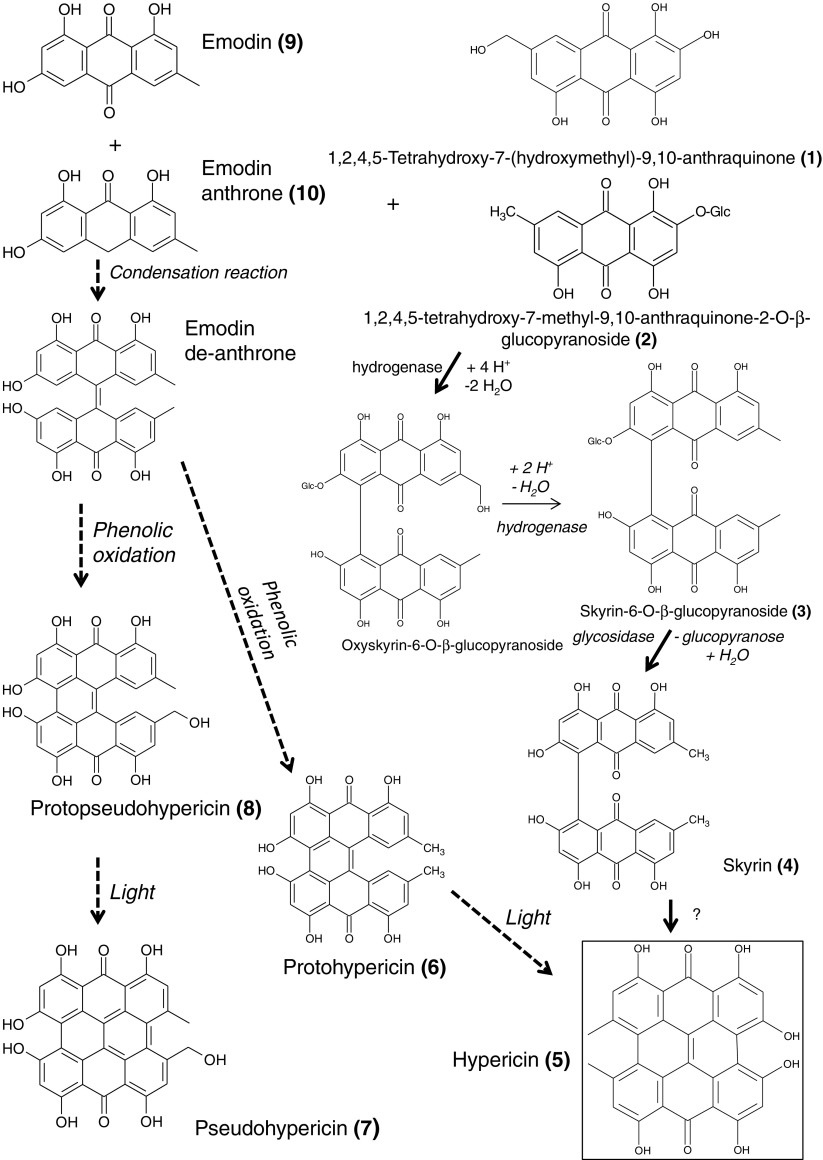
Structural formulae of putative precursors in two suggested metabolic pathways for hypericin 5, pseudohypericin 7, and their protoforms (protohypericin 6 and protopseudohypericin 8). Emodin 9 and emodin anthrone 10—dashed line; 1,2,4,5-tetrahydroxy-7-(hydroxymethyl)-9,10-anthraquinone 1, 1,2,4,5-tetrahydroxy-7-methyl-9,10-anthraquinone-2-O-β-glucopyranoside 2, and skyrin 4—solid line (pathways based on the results of previous studies [11, 17])

The metabolomic study of species within the genus *Hypericum* revealed a broad spectrum of secondary metabolites, and consecutively identified components displayed different biosynthetic potential. LC-MS analysis enabled the recognition of 34 compounds including anthraquinone/naphthodianthrones (1–10), phloroglucinols (23–27, 34), flavonoids (28–31), chlorogenic acid derivatives (11–22), and a xanthone (33), each with different abundances in the studied species (Table 1, Scheme 1, and Electronic Supplementary Material (ESM) Figs. S1, S3, and S4).

**Table 1:** List of metabolites identified in 17 *Hypericum* spp. The occurrence in individual species is shown in ESM Fig. S2—boxplots 1–35

No.	Putatively identified metabolite	RT [min]	MW [Da]	Elemental composition [C_n_H_m_O_x]_	[M-H] ¯ [*m/z*]				Level of identification (A-standard; B-MS/MS)
					Calculated	Obtained	Mass error		
							ppm	mDa	
1.	1,2,4,5-Tetrahydroxy-7-(hydroxymethyl)-9,10-anthraquinone	4.3	302	C_15_H_10_O_7_	301.0353	301.0349	1.33	0.4	B
2.	1,2,4,5-Tetrahydroxy-7-methyl-9,10-anthraquinone-2-O-β-glucopyranoside	4.0	448	C_21_H_20_O_11_	447.0932	447.0973	− 9.17	− 4.1	B
3.	Skyrin-6-O-β-glucopyranoside	9.8	700	C_36_H_28_O_15_	699.1355	699.1413	− 8.30	− 5.8	B
4.	Skyrin (2,2′,4,4′,5,5′-hexahydroxy-7,7′-dimethyl-1,1′-bianthracene-9,9′,10,10′-tetrone)	9.4	538	C_30_H_18_O_10_	537.0827	537.0869	− 7.82	− 4.2	B
5.	Hypericin (4,4′,5,5′,7,7′-hexahydroxy-2,2′-dimethylnaphthodianthrone)	14.2	504	C_30_H_16_O_8_	503.0772	503.0749	4.57	2.3	A
6.	Protohypericin (1,3,4,6,8,15-hexahydroxy-10,13-dimethyldibenzo[a,o]perylene-7,16-dione)	13.2	506	C_30_H_18_O_8_	505.0928	505.0911	3.37	1.7	A
7.	Pseudohypericin (1,3,4,6,8,13-hexahydroxy-10-(hydroxymethyl)-11-methylphenanthro[1,10,9,8-opqra]perylene-7,14-dione)	11.1	520	C_30_H_16_O_9_	519.0721	519.0669	10.02	5.2	A
8.	Protopseudohypericin (1,3,4,6,8,15-hexahydroxy-10-(hydroxymethyl)-13-methyldibenzo[a,o]perylene-7,16-dione)	10.2	522	C_30_H_18_O_9_	521.0878	521.0826	9.98	5.2	A
9.	Emodin (1,3,8-trihydroxy-6-methylanthracene-9,10-dione)	7.2	270	C_15_H_10_O_5_	269.0455	269.0459	− 1.49	− 0.4	B
10.	Emodin anthrone (1,3,8-trihydroxy-6-methyl-10H-anthracen-9-one)	7.2	254	C_15_H_12_O_4_	255.0662	255.0629	12.94	3.3	B
11.	Chlorogenic acid (3-*O*-caffeoylquinic acid)	3.8	354	C_16_H_18_O_9_	353.0878	353.0884	− 1.70	− 0.6	A
12.	Caffeoylquinic acid isomer—CQA I^a^	3.7	354	C_16_H_18_O_9_	353.0878	353.0872	1.70	0.6	B
13.	Caffeoylquinic acid isomer—CQA II^a^	4.0	354	C_16_H_18_O_9_	353.0878	353.0877	0.28	0.1	B
14.	Caffeoylquinic acid isomer—CQA III^a^	4.4	354	C_16_H_18_O_9_	353.0878	353.0865	3.68	1.3	B
15.	Dicaffeoylquinic acid isomer—diCQA I^a^	0.7	516	C_25_H_24_O_12_	515.1195	515.1204	− 1.75	− 0.9	B
16.	Dicaffeoylquinic acid isomer—diCQA II^a^	2.9	516	C_25_H_24_O_12_	515.1195	515.1211	− 3.11	− 1.6	B
17.	Feruoylquinic acid isomer—FerQA I^a^	4.0–4.1	368	C_17_H_20_O_9_	367.1035	367.1044	− 2.45	− 0.9	B
18.	Feruoylquinic acid isomer—FerQA II^a^	4.7	368	C_17_H_20_O_9_	367.1035	367.1045	− 2.72	− 1	B
19.	Coumaroylquinic acid isomer—CouQA I^a^	3.7	338	C_16_H_18_O_8_	337.0929	337.0934	− 1.48	− 0.5	B
20.	Coumaroylquinic acid isomer—CouQA II^a^	4.3–4.4	338	C_16_H_18_O_8_	337.0929	337.0939	− 2.97	− 1	B
21.	Coumaroylquinic acid isomer—CouQA III^a^	4.6	338	C_16_H_18_O_8_	337.0929	337.0923	1.78	0.6	B
22.	Coumaroylquinic acid isomer—CouQA IV^a^	4.8	338	C_16_H_18_O_8_	337.0929	337.0939	− 2.97	− 1	B
23.	Hyperforin ((1R,5R,6R,7S)-2-hydroxy-6-methyl-1,3,7-tris(3-methylbut-2-enyl)-6-(4-methylpent-3-enyl)-5-(2-methylpropanoyl)bicyclo[3.3.1]non-2-ene-4,9-dione)	15.9	536	C_35_H_52_O_4_	535.3793	535.3798	− 0.93	− 0.5	A
24.	Adhyperforin (6-methyl-5-(2-methylbutanoyl)-1,3,7-tris(3-methylbut-2-enyl)-6-(4-methylpent-3-enyl)-4,9-dioxobicyclo[3.3.1]non-2-en-2-olate)	16.2	550	C_36_H_54_O_4_	549.3949	549.3934	2.73	1.5	B
25.	Furohyperforin ((1S,3S,8R,9R,10S)-3-(2-hydroxy-2-propanyl)-8-isobutyryl-9-methyl-6,10-bis(3-methyl-2-buten-1-yl)-9-(4-methyl-3-penten-1-yl)-4-oxatricyclo[6.3.1.01,5]dodec-5-ene-7,12-dione)	14.3	552	C_35_H_52_O_5_	551.3742	551.3725	3.08	1.7	B
26.	Hyperfirin (8S)-4-hydroxy-1-isobutyryl-8-methyl-3,5-bis(3-methyl-2-buten-1-yl)-8-(4-methyl-3-penten-1-yl)bicyclo[3.3.1]non-3-ene-2,9-dione)	14.8	468	C_30_H_44_O_4_	467.3167	467.3164	0.64	0.3	B
27.	Adhyperfirin ((1R,5R,6S)-2-hydroxy-6-methyl-5-(2-methylbutanoyl)-1,3-bis(3-methylbut-2-enyl)-6-(4-methylpent-3-enyl)bicyclo[3.3.1]non-2-ene-4,9-dione)	16.5	482	C_31_H_46_O_4_	481.3323	481.3311	2.49	1.2	B
28.	Quercetin (2-(3,4-dihydroxyphenyl)-3,5,7-trihydroxy-4H-chromen-4-one)	6.6	302	C_15_H_10_O_7_	301.0354	301.0355	− 0.33	− 0.1	A
29.	Quercitrin (quercetin 3-rhamnoside)	5.5	448	C_21_H_20_O_11_	447.0932	447.0973	− 9.17	− 4.1	B
30.	Hyperoside (quercetin 3-*O*-galactoside)	5.1	464	C_21_H_20_O_12_	463.0882	463.0908	− 5.61	− 2.6	A
31.	Rutin (quercetin 3-*O*-glucosyl-rhamnoside)	5.0	610	C_27_H_30_O_16_	609.1461	609.1507	− 7.55	− 4.6	B
32.	Cryptosporin quinate	6.4	450	C_21_H_22_O_11_	449.1089	449.1073	3.56	1.6	B
33.	Mangiferin	4.2	422	C_19_H_18_O_11_	421.0776	421.0771	1.19	0.5	B
34.	4-Hydroxy-1-isobutyryl-8-methyl-3,7,8-tris(3-methyl-2-buten-1-yl)-5-(2-methyl-1-propen-1-yl)bicyclo[3.3.1]non-3-ene-2,9-dione	15.3	508	C_33_H_48_O_4_	507.3479	507.3516	− 7.29	− 3.7	B

^a^Substitution by phenylpropanoid acid was not defined

The presence of the bioactive compounds was proven based on retention times that were registered on single-ion chromatograms drawn for the monoisotopic masses of deprotonated molecules [M-H]^−^ and their exact *m/z* values that were registered in mass spectra (MS mode). Additionally, collision-induced dissociation mass spectra (CID MS/MS) with recognized product ions facilitated the definition of target metabolite chemical structures (ESM Table S1). Some compounds were putatively identified based on the spectra and retention times that were registered for available standards (indicated in Table 1).

### New compounds in the genus *Hypericum*

Several compounds were detected that have not previously been identified in the genus *Hypericum*. We putatively determined the presence of two new anthraquinones, 1,2,4,5-tetrahydroxy-7-(hydroxymethyl)-9,10-anthraquinone (1) and 1,2,4,5-tetrahydroxy-7-methyl-9,10-anthraquinone-2-O-ß-glucopyranoside (2). 1,2,4,5-Tetrahydroxy-7-(hydroxymethyl)-9,10-anthraquinone was found in 11 *Hypericum* spp., with the highest relative abundance in *H. humifusum*, followed by *H. perforatum*, *H. androsaemum*, and *H. rumeliacum* (ESM Fig. S2). 1,2,4,5-Tetrahydroxy-7-methyl-9,10-anthraquinone-2-O-ß-glucopyranoside was present in six *Hypericum* spp. and was most abundant in *H. rumeliacum* (ESM Fig. S2)*.* Among the other anthraquinones, skyrin (4) and skyrin-6-O-ß-glucopyranoside (3), which were previously found only in *H. perforatum*, were detected in all hypericin-producing *Hypericum* spp. (ESM Fig. S2).

Based on the analysis of fragmentation spectra in both the negative and positive ionization modes, a new cryptosporin ester of quinic acid, cryptosporin quinate (MW 450 Da), was also putatively identified in several *Hypericum* representatives, with the highest relative abundance in *H. annulatum* (ESM Figs. S2 and S4).

In addition, an unidentified compound with an [M-H]^−^ at *m/z* 507.3479 was present in some of the studied *Hypericum* species. The MS/MS spectrum and proposed chemical structure of the compound (34), whose highest relative abundance was in *H. perforatum* (ESM Fig. S2), are shown in ESM Fig. S4. The exact measured monoisotopic mass of this compound suggests its composition to be C_33_H_48_O_4_. A hyperforin derivative with this composition can be found in the chemical databases ChemSpider and PubChem (ChemSpider ID 8007183). The fragmentation pattern obtained for this compound showed typical alkyl or hydroxyl alkyl group losses, which strongly suggests that it belongs to the phloroglucinol class and even shows that it is likely to be a hyperforin derivative. Of course, to confirm the structure of this compound (34), other identification methods such as NMR spectroscopy need to be performed.

### PCA and HCA of identified metabolites

A complete spectrum of the secondary metabolites detected in the present study was subjected to PCA and hierarchical clustering analysis (HCA) to identify compounds that co-existed with the profiled metabolites in *Hypericum* spp., especially hypericins, to reveal potential intermediates in hypericin biosynthesis.

To compare the selected sets of metabolites of the *Hypericum* spp. to each other, we performed principal component analysis. PCA changes large data sets into elements placed in the orthogonal space, resulting in components capturing the majority of variation. Usually, two or three main components are responsible for more than 90% of data variability. In our case, the first two components, PC1 and PC2, composed 52.7% of the total metabolites variability in the studied species. The first principal component (PC1) has large positive associations with group of compounds comprising anthraquinones, and it accounted for 31.9% of the metabolite variability. The second component (PC2) has clearly positive association with phloroglucinol derivatives, and explained 20.8% variability. The relatively low values of the first two PCs accounted for a large number of the variables that significantly distinguished the studied sample groups.

Based on an analysis of the PCA loadings, three main groups of secondary metabolites were distinguished (Fig. 2). First, the relatively homogenous group constituting PC1 (high, positive values in range 2–4) was mainly composed of the anthraquinones/naphthodianthrones hypericin (5), pseudohypericin (7), and their protoforms protohypericin (6) and protopseudohypericin (8), followed by 1,2,4,5-tetrahydroxy-7-methyl-9,10-anthraquinone-2-O-ß-glucopyranoside (2), skyrin-6-O-ß-glucopyranoside (3), and skyrin (4) (ESM Fig. S2). Cryptosporin quinate (32) was associated with this group as well (ESM Fig. S2).

The second homogenous group, positively associated with PC2, consisted of the phloroglucinols hyperforin (23), adhyperforin (24), furohyperforin (25), hyperfirin (26), adhyperfirin (27), and the new compound previously unidentified in *Hypericum* with an [M-H]^−^ at *m/z* 507.3479 (34) (ESM Fig. S2 and S4).

The remaining cluster of metabolites was a heterogeneous group of compounds including flavonoids (28–31) (ESM Fig. S2), chlorogenic acid derivatives (11–22) (ESM Fig. S2) and, surprisingly, the anthraquinones emodin (9) (ESM Fig. S2), emodin anthrone (10) (ESM Fig. S2), and 1,2,4,5-tetrahydroxy-7-(hydroxymethyl)-9,10-anthraquinone (1) (ESM Fig. S2). In addition, the xanthone glucoside mangiferin (33) (ESM Fig. S2) was separated on the loading plot.

Considering the PCA results and the abundances of particular metabolites in the studied *Hypericum* spp., the presumed hypericin derivatives were determined to be most abundant in *H. rumeliacum*, *H. humifusum*, *H. tetrapterum*, and *H. annulatum*. Phloroglucinol derivatives prevailed in *H. androsaemum*, *H. stellatum*, and *H. kouytchense*, all of which are hypericin-lacking species. *H. perforatum* was the only species that contained both naphthodianthrones and phloroglucinols in high amounts (ESM Fig. S2). In contrast, *H. bupleuroides*, *H. canariense*, *H. balearicum*, and *H. pulchrum* were rich with diverse flavonoids and chlorogenic acid derivatives.

**Fig. 1:**
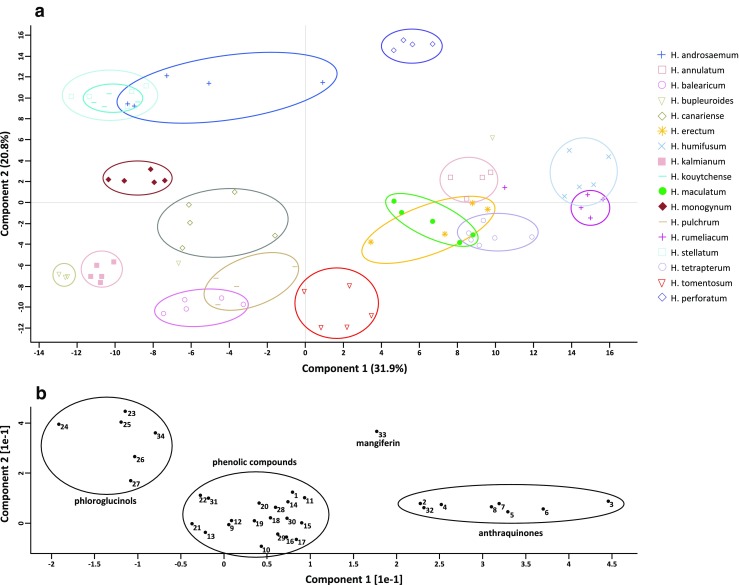
PCA showing the relations between the major groups of metabolites in *Hypericum* spp. Projection of the analyzed plant samples (**A**). Spatial component score of the metabolites (**B**). The anthraquinone glucopyranoside 2, skyrin glucopyranoside 3, skyrin 4, and cryptosporin quinate 32 were correlated with hypericin 5, pseudohypericin 7, and their protoforms 6 and 8

The variability in the abundances of secondary metabolites within the studied *Hypericum* spp. was also evident from the HCA. In fact, the dendrogram of metabolites showed clusters of compounds that commonly occur in certain groups of *Hypericum* spp. Briefly, the HCA separated the studied *Hypericum* spp. into two main clusters (Fig. 1). Examining the heat map, *Hypericum* species that accumulate hypericin in the leaves were positioned on the right side of the dendrogram, while species that lack hypericin and have higher abundances of phloroglucinols formed the left cluster. Moreover, the species *H. perforatum* and *H. annulatum*, which formed a separate subcluster on one side of the dendrogram, and *H. balearicum*, which was on the opposite side, had the most distinguished metabolic profiles among the studied species.

### The possible role of skyrin and other anthraquinones in hypericin biosynthesis

**Fig. 2:**
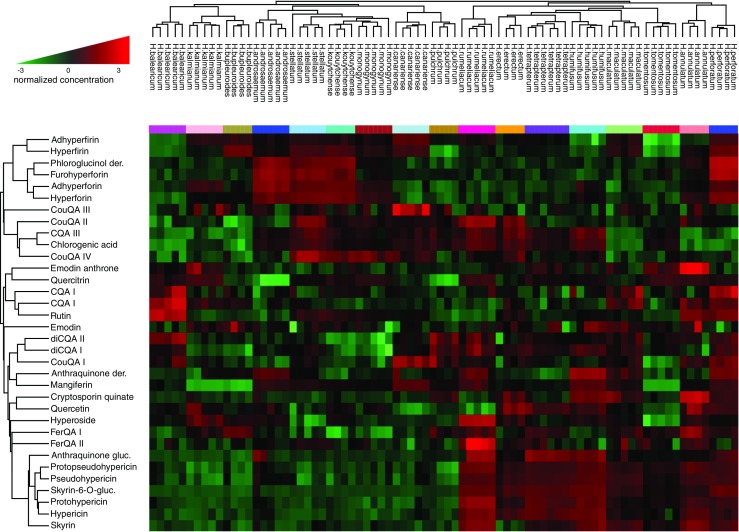
Heat map and hierarchical clustering of *Hypericum* spp. and metabolites

HCA and PCA enabled the identification of correlations between analyzed compounds in the set of 17 *Hypericum* spp. The results of both statistical analyses demonstrated that skyrin (4), skyrin-6-O-ß-glucopyranoside (3), and the newly putatively identified 1,2,4,5-tetrahydroxy-7-methyl-9,10-anthraquinone-2-O-β-glucopyranoside (2) formed a cluster with other naphthodianthrones, namely, hypericin and its derivatives and protoforms (Figs. 1 and 2). In addition, the occurrence of skyrin was strikingly correlated with the occurrence of hypericin in the studied *Hypericum* spp. (ESM Fig. S2). Moreover, skyrin-6-O-ß-glucopyranoside (3) and 1,2,4,5-tetrahydroxy-7-methyl-9,10-anthraquinone-2-O-β-glucopyranoside (2) were also present only in the species that accumulate hypericin (ESM Fig. S2). In contrast, emodin and emodin anthrone, commonly thought to be precursors of hypericin, were also found in hypericin-lacking species. Despite the fact that emodin and emodin anthrone are supposed to be precursors of hypericin, our findings indicate potential roles for the abovementioned compounds (2, 3, 4) in hypericin biosynthesis.

## Discussion

Representatives of the genus *Hypericum* contain a wide range of bioactive compounds, among which naphthodianthrones and phloroglucinols, with assumed synergistic effects from other groups of secondary metabolites, especially contribute to their biological activities.

In our study, PCA defined phloroglucinols and anthraquinones as two principal components, PC1 and PC2, when grouping the compounds based on their chemical structure and occurrence in the studied *Hypericum* spp. In general, phloroglucinols were most abundant in *Hypericum* spp. that lack hypericins (*H. androsaemum*, *H. stellatum*, and *H. kouytchense*), which is in accordance with previous studies [6, 7, 15, 18]. In contrast, species with a higher abundance of naphthodianthrones contained fewer or no phloroglucinols, except for *H. perforatum*, which was determined to have a high content of both of these metabolite groups.

The present metabolomic study was performed to examine a spectrum of secondary metabolites not previously identified in the genus *Hypericum* and to identify compounds present in the same PC1 group as hypericin to elucidate its potential precursors.

LC/MS analysis enabled the detection of several new compounds. A compound with an [M-H]^−^ at *m/z* 507.3479 (34) was identified with highest relative abundance in *H. perforatum*, followed by *H. androsaemum*, *H. kouytchense*, *H. stellatum*, *H. annulatum*, and *H. monogynum.* Based on both the PCA and HCA results as well as the relationships of this compound with phloroglucinols putatively determined from the MS/MS spectra, the proposed chemical structure of this compound, which has the molecular formula C_33_H_47_O_4_, indicates that it is probably a new phloroglucinol (the proposed structure can be found in the ChemSpider database). A compound with an identical molecular formula was previously detected as an unidentified compound in *H. perforatum* [19] and in the flower extracts of six *Hypericum* spp. [4]. Our results confirm the presence of this compound in the leaves of the three species, *H. perforatum*, *H. androsaemum*, and *H. kouytchense*, along with the flowers analyzed by [4], and we report for the first time its occurrence in *H. stellatum*, *H. annulatum*, and *H. monogynum.*

Another new compound, tentatively proposed to be cryptosporin quinate (32), has not been included in any chemical database to date. This compound is probably derived from cryptosporin, which has previously been isolated from the fermentation broths of *Cryptosporium pinicola* Linder [20]. However, the correlation between the occurrence of cryptosporin quinate and hypericins in the studied *Hypericum* spp., as revealed by PCA and HCA, was unexpected given the differences in their chemical structures.

The biosynthesis of hypericin, the principle bioactive compound produced by some *Hypericum* spp., remains not well understood. The anthraquinones emodin and/or emodin anthrone are considered intermediates in hypericin biosynthesis even though they are distributed in many plant species worldwide [8] (Scheme 1). We putatively identified three anthraquinones, namely, 1,2,4,5-tetrahydroxy-7-methyl-9,10-anthraquinone-2-O-ß-glucopyranoside (2), skyrin-6-O-ß-glucopyranoside (3), and skyrin (4), as other possible precursors of hypericin that correlated with the presence of hypericin, its protoforms, and derivatives in the studied *Hypericum* spp. Contrary to 1,2,4,5-tetrahydroxy-7-methyl-9,10-anthraquinone-2-O-ß-glucopyranoside (2), which was tentatively identified in the genus *Hypericum* for the first time, skyrin and the four bisanthraquinone glycosides S-(+)-skyrin-6-O-b-glucopyranoside, R-(−)-skyrin-6-O-b-glucopyranoside, S-(+)-skyrin-6-O-b-xylopyranoside, and S-(+)-skyrin-6-O-b-a-arabinofuranoside had already been identified in aerial parts of *H. perforatum* by [12]. Similarly, R-(−)-skyrin-6-O-β-D-xylopyranoside was found in *H. sampsonii* [13], and skyrin-2-O-glucopyranoside was detected in the flower extracts of six *Hypericum* spp. by [4]. This work provides the first report of skyrin and (2) in other *Hypericum* spp., all of which accumulate hypericin in their leaves. Skyrin (4) and both anthraquinone glycosides, 1,2,4,5-tetrahydroxy-7-methyl-9,10-anthraquinone-2-O-ß-glucopyranoside (2) and skyrin-6-O-ß-glucopyranoside (3), were not detected in any of the hypericin-lacking *Hypericum* spp.

The other putatively identified anthraquinones, 1,2,4,5-tetrahydroxy-7-(hydroxymethyl)-9,10-anthraquinone (1), emodin (9), and emodin anthrone (10), did not correlate with the presence of hypericins. Recent results have not been completely consistent with the previously published assumption that emodin is the key intermediate in hypericin biosynthesis [6, 7, 21]. Moreover, studies [15, 16] revealed the presence of emodin and emodin anthrone in non-hypericin-accumulating species from the genera *Ascyreia* and *Webbia*, thus corroborating our present results (ESM Fig. S2).

The occurrence rates of emodin and skyrin are different in higher plants. While emodin is present in many plant families [8], skyrin is rare and has been found only in *H. perforatum* [10], the mangrove *Kandelia rheedii* Wight & Arn. [22], and *Ventilago leiocarpa* Benth. (Rhamnaceae) [23]. Conversely, skyrin, which was discovered by Howard and Raistrick [24], was isolated from *Penicillium islandicum* Sopp. and was later found together with hypericin and pseudohypericin in the insect superfamily Coccoidea [25, 26] as well as in several fungi and lichens [12, 27–29].

The metabolomic analysis of 17 *Hypericum* spp., including species that contain and lack hypericin, proved that skyrin and the anthraquinone glycosides (2) and (3) are present exclusively in hypericin-producing *Hypericum* spp., while emodin was detected in all analyzed species. Naphthodianthrones are closely connected to skyrin (4) and its glucoside (3) as well as 1,2,4,5-tetrahydroxy-7-methyl-9,10-anthraquinone-2-O-β-glucopyranoside (2) and a group comprising feruoylquinic acid derivatives (17, 18) (Fig. 1). In turn, emodin and emodin anthrone, both of which were linked to different subclusters, did not correlate with the presence of hypericins. These results favor skyrin as a possible key intermediate in hypericin biosynthesis (Scheme 1).

This work builds upon our recent metabolomic studies performed with the same set of *Hypericum* spp. [7, 16]. In this paper, we tentatively identified new compounds present in the genus *Hypericum* and proposed that the anthraquinone skyrin is the key intermediate in hypericin biosynthesis. This anthraquinone, along with several others, is common in fungi [30] but rare in higher plants, suggesting there is some similarity in the metabolic pathways between *Hypericum* and these organisms. This possible crosstalk should also be investigated in the future.

## Materials and methods

### Plant material and culture conditions

Selected *Hypericum* species (*H. perforatum* L., *H. maculatum* Crantz, *H. androsaemum* L., *H. humifusum* L., *H. bupleuroides* Griseb., *H. kalmianum* L., *H. annulatum* Moris, *H. balearicum* L., *H. tomentosum* L., *H. tetrapterum* Fr., *H. kouytchense* Levl., *H. pulchrum* L., *H. erectum* Thunb., *H. stellatum* N. Robson, *H. monogynum* L., *H. canariense* L., and *H. rumeliacum* Boiss) were used in the experiment.

Seeds of the *Hypericum* spp. were acquired via the International Seed Exchange Program *Index seminum*, and they were cultivated in vitro on solid basal medium containing Murashige and Skoog’s salt mixture [31] enriched with Gamborg’s B5 vitamins [32], 30.0 g l^−1^ of sucrose, 2.0 mg l^−1^ of glycine, and 7.0 g l^−1^ of agar. The pH of the medium was adjusted to 5.6 before autoclaving. The in vitro propagated *Hypericum* plants were grown in a culture room at ± 23 °C with 34% relative humidity, a 16/8-h photoperiod, and 32 μM/m^2^/s of photosynthetically active radiation (PAR).

All *Hypericum* spp. were characterized cytogenetically by chromosome number counts and DNA content according to the procedure described by Bruňáková and Čellárová [33]. Species with ambiguous results were identified by DNA barcoding using the *psbA-trnH* intergenic spacer of their cpDNA [21].

For metabolome analyses, the leaves of the *Hypericum* spp. a week after subculturing were isolated, immediately frozen in liquid nitrogen, and stored at − 80 °C prior to metabolite extraction.

### Metabolite extraction

Frozen leaves from the *Hypericum* spp. were ground in liquid nitrogen in precooled adaptors for 45 s at a frequency of 30 Hz using an MM400 ball mill (Retsch, Germany). Then, 100 mg of each frozen sample (five replicates for each *Hypericum* sp.) was suspended in 4 ml of 100% methanol. The Genistein analytical standard (Sigma-Aldrich, USA) was used as an internal standard (6 μl of 1.0 mg/ml solution per sample). The mixtures were shaken vigorously for 10 min at room temperature in a thermomixer (TS-100, Biosan, Latvia) at 950 rpm. The suspensions were then centrifuged at 11,000*g* at room temperature (RT), and the supernatants were evaporated at RT using a vacuum concentrator (Eppendorf, Germany). The dried extract samples were re-dissolved in 500 μl of 100% methanol prior to LC-MS analysis.

### Metabolite profiling using LC-MS

LC-MS measurements were performed with a Waters ACQUITY ultra performance liquid chromatography (UPLC) system (Milford, MA, USA) connected to a micrOTOF-Q mass spectrometer from Bruker Daltonics (Bremen, Germany). A Poroshell 120 EC-C18 column (Agilent, USA) with a size and granulation of 2.1 × 100 mm and 1.8 μm, respectively, was used. Chromatographic separation was performed at a 0.6 ml/min flow rate using mixtures of two solvents, A (99.5% H_2_O/0.5% formic acid *v*/*v*) and B (99.5% acetonitrile/0.5% formic acid *v*/*v*), with a 2:1 split of the column effluent, 0.2 ml/min of which was delivered to the ESI ion source. The elution steps were as follows: 0–5 min, linear gradient from 10 to 30% of B; 5–12 min, isocratic at 30% of B; 12–13 min, linear gradient from 30 to 95% of B; and 13–15 min, isocratic at 95% of B. After returning to the initial conditions, equilibration was achieved in 4 min. The micrOTOF-Q mass spectrometer consisted of an ESI source operating at a voltage of ± 4.5 kV with nitrogen nebulization at 1.2 bars and a dry gas flow of 8.0 L min^−1^ at a temperature of 220 °C. The instrument was operated using the program micrOTOF control ver. 2.3, and the data were analyzed using the Bruker data analysis ver. 4 package. The system was calibrated externally using a calibration mixture containing sodium formate clusters. Additional internal calibration was performed for every run by injecting the calibration mixture via the diverter valve during the LC separation. All calculations were performed using the high-performance computing (HPC) quadratic algorithm. Our calibration gave the *m/z* value measurements an accuracy of at least 5 ppm.

To identify the compounds, the instrument was operated in the CID MS/MS mode. These targeted MS/MS experiments were performed using a collision energy ramp from 15 to 30 eV (positive ion mode) and from 25 to 30 eV (negative ion mode). For the pseudo-MS^3^ experiments, the in-source collision energy (ISCID) was increased from 0 to 80 or 85 eV in the positive and negative ion modes, respectively. The instrument was operated at a resolution greater than 17,000 full width at half maximum (FWHM). The spectra were recorded in the targeted mode within the *m/z* mass range of 50–1000.

### Statistical analyses

Chromatographic peaks of the putatively identified compounds traced in the extracted ion chromatograms (EIC) were integrated and corresponding areas of each peak were transferred to Excel. Internal standard (genistein) was used only to control reproducibility of sample preparation and injection. Peak of the internal standard was measured in each sample and in case its value was different by more than 25% from the references, such sample was rejected and analysis was repeated. We decided to use only single internal standard due to the assumption of performing non-targeted analyses, thereby we were unable to provide an internal standard for each potentially identified compound. For statistical correctness, data were normalized to TIC and each *Hypericum* species was analyzed in four biological replicates. Boxplots for each identified compound were prepared directly in Excel.

For the metabolic profiling data, two types of statistical analyses were performed, HCA and PCA. All calculations were completed using Perseus software (Max Planck Institute of Biochemistry, Germany [34]). Perseus is a statistical package devoted to the analysis of large proteomic data, but its functions can also be used seamlessly to calculate metabolomic data with a similar structure. For our purposes, Excel files were exported in csv format for Perseus analysis. Next, all numeric values were transformed to a logarithmic scale, and all samples were grouped using categorical annotation. Missing values were then replaced by imputation for hierarchical clustering purposes. Data were normalized for each sample with Z-score algorithm using median values for Z-score calculation. After all transformations, PCA analysis was performed and then, for the clustering analysis, the data were again normalized for each compound using the Z-score algorithm.

## Electronic supplementary material

ESM 1 (PDF 5771 kb)

## References

[1] Crockett SL, Robson NKB (2011). Taxonomy and chemotaxonomy of the genus Hypericum. Med Aromat Plant Sci Biotechnol.

[2] Herbal supplements and remedies. http://www.strategyr.com/MarketResearch/Herbal_Supplements_and_Remedies_Market_Trends.asp. Accessed 23 Jan 2018.

[3] Stojanović G, Ðorđević A, Šmelcerović A (2013). Do other Hypericum species have medical potential as St. John’s wort (Hypericum perforatum)?. Curr Med Chem.

[4] Porzel A, Farag MA, Mülbradt J, Wessjohann LA (2014). Metabolite profiling and fingerprinting of Hypericum species: a comparison of MS and NMR metabolomics. Metabolomics.

[5] Jendželovská Z, Jendželovský R, Kuchárová B, Fedoročko P (2016). Hypericin in the light and in the dark: two sides of the same coin. Front Plant Sci.

[6] Kusari S, Zühlke S, Borsch T, Spiteller M (2009). Positive correlations between hypericin and putative precursors detected in the quantitative secondary metabolite spectrum of Hypericum. Phytochemistry.

[7] Nigutová K, Kusari S, Sezgin S, Petijová L, Henzelyová J, Bálintová M, et al. Chemometric evaluation of hypericin and related phytochemicals in 17 *in vitro* cultured *Hypericum* species, hairy root cultures and hairy root-derived transgenic plants. J Pharm Pharmacol. 2017. 10.1111/jphp.12782. 10.1111/jphp.12782 28722156

[8] Izhaki I (2002). Emodin—a secondary metabolite with multiple ecological functions in higher plants. New Phytol.

[9] Gill M, Gimenez A, McKenzie RW (1988). Pigments of fungi, part 8. Bianthraquinones from dermocybe a ustroveneta. J Nat Prod.

[10] Berghöfer R (1987) Analytik und Isolierung phenolischer Inhaltsstoffe von Hypericum perforatum L. aus Anbau und Wildvorkommen und Vergleich mit anderen heimischen Hypericum-Arten. J Cramer.

[11] Hoelzl J, Petersen M (2003). Chemical constituents of Hypericum spp. Med Aromat Plants--Industrial Profiles.

[12] Wirz A, Simmen U, Heilmann J, Çalis I, Meier B, Sticher O (2000). Bisanthraquinone glycosides of Hypericum perforatum with binding inhibition to CRH-1 receptors. Phytochemistry.

[13] Don M-J, Huang Y-J, Huang R-L, Lin Y-L (2004). New phenolic principles from Hypericum sampsonii. Chem Pharm Bull (Tokyo).

[14] Jie M (2012). Chemical constituents from Hypericum perforatum. China J Chinese Mater Medica.

[15] Kucharíková A, Kimáková K, Janfelt C, Čellárová E (2016). Interspecific variation in localization of hypericins and phloroglucinols in the genus Hypericum as revealed by desorption electrospray ionization mass spectrometry imaging. Physiol Plant.

[16] Kucharíková A, Kusari S, Sezgin S, Spiteller M, Čellárová E (2016). Occurrence and distribution of phytochemicals in the leaves of 17 in vitro cultured Hypericum spp. adapted to outdoor conditions. Front Plant Sci.

[17] Zobayed SMA, Afreen F, Goto E, Kozai T (2006). Plant-environment interactions: accumulation of hypericin in dark glands of Hypericum perforatum. Ann Bot.

[18] Kusari S, Sezgin S, Nigutova K, Cellarova E, Spiteller M (2015). Spatial chemo-profiling of hypericin and related phytochemicals in Hypericum species using MALDI-HRMS imaging. Anal Bioanal Chem.

[19] Farag M, Wessjohann L (2012). Metabolome classification of commercial *Hypericum perforatum* (St. John’s wort) preparations via UPLC-qTOF-MS and chemometrics. Planta Med.

[20] Gupta RB, Franck RW (1989). The total synthesis of (-)-cryptosporin. J Am Chem Soc.

[21] Košuth J, Smelcerovic A, Borsch T, Zuehlke S, Karppinen K, Spiteller M, Hohtola A, Čellárová E (2011). The *hyp*-*1* gene is not a limiting factor for hypericin biosynthesis in the genus *Hypericum*. Funct Plant Biol.

[22] Zaman A (2012). Docking studies and network analyses reveal capacity of compounds from Kandelia rheedii to strengthen cellular immunity by interacting with host proteins during tuberculosis infection. Bioinformation.

[23] Lin LC, Chou CJ, Kuo YC (2001). Cytotoxic principles from Ventilago leiocarpa. J Nat Prod.

[24] Howard BH, Raistrick H (1949). Studies in the biochemistry of micro-organisms. 80. The colouring matters of Penicillium islandicum Sopp. Part 1. 1:4:5-trihydroxy-2-methylanthraquinone. Biochem J.

[25] Banks H, Cameron D, Raverty W (1976). Chemistry of the Coccoidea. II. Condensed polycyclic pigments from two Australian pseudococcids (Hemiptera). Aust J Chem.

[26] Cameron D, Raverty W (1976). Pseudohypericin and other phenanthroperylene quinones. Aust J Chem.

[27] Hermawati, E., Hakim, E.H., Juliawaty LD (2014) Secondary metabolites of endophytic fungi from Morus plant. In: Nat Prod Chem Res, 2, 2014, 5 (2nd International Conference and Exhibition on Pharmacognosy, Phytochemistry & Natural Products, August 25–27). Beijing, China.

[28] Zhai M-M, Li J, Jiang C-X, Shi Y-P, Di D-L, Crews P, Wu Q-X (2016). The bioactive secondary metabolites from Talaromyces species. Nat Prod Bioprospect.

[29] Jahn L, Schafhauser T, Wibberg D, Rückert C, Winkler A, Kulik A, Weber T, Flor L, van Pée K-H, Kalinowski J, Ludwig-Müller J, Wohlleben W (2017). Linking secondary metabolites to biosynthesis genes in the fungal endophyte Cyanodermella asteris: the anti-cancer bisanthraquinone skyrin. J Biotechnol.

[30] Bräse S, Gläser F, Kramer CS, Lindner S, Linsenmeier AM, Masters K-S, Meister AC, Ruff BM, Zhong S. Skyrins. 2013. p. 139–151. 23781707

[31] Murashige T, Skoog F (1962). A revised medium for rapid growth and bio assays with tobacco tissue cultures. Physiol Plant.

[32] Gamborg OL, Miller RA, Ojima K (1968). Nutrient requirements of suspension cultures of soybean root cells. Exp Cell Res.

[33] Bruňáková Katarína, Čellárová Eva (2016). Shoot Tip Meristem Cryopreservation of Hypericum Species. Methods in Molecular Biology.

[34] Tyanova S, Temu T, Sinitcyn P, Carlson A, Hein MY, Geiger T, Mann M, Cox J (2016). The Perseus computational platform for comprehensive analysis of (prote)omics data. Nat Methods.

