# Supplementary material for: Phenotyping the genus Hypericum by secondary metabolite profiling: emodin vs. skyrin, two possible key intermediates in hypericin biosynthesis
Source: Anal Bioanal Chem. 2018 Oct 5;410(29):7689–99. doi: 10.1007/s00216-018-1384-0 (PMC6244766; doi:10.1007/s00216-018-1384-0)
Supplement: Supplementary file 1 — (PDF 5771 kb) [file 216_2018_1384_MOESM1_ESM.pdf]

Analytical and Bioanalytical Chemistry

Electronic Supplementary Material

Phenotyping the genus *Hypericum* by secondary metabolite profiling: emodin vs. skyrin, two possible key intermediates in hypericin biosynthesis

Katarína Kimáková, Andrea Kimáková, Jakub Idkowiak, Maciej Stobiecki Paweł Rodziewicz, Łukasz Marczak, Eva Čellárová

Fig. S1 Structural formulae of the identified metabolites excluding those depicted in Scheme 1, S3 and S4

R^1, R^2 = OH – Caffeoylquinic acid (Chlorogenic acid) **(11-14)**

R^1, R^2 = OH – Dicaffeoylquinic acid **(15, 16)**

R^1 = OCH_3, R^2 = OH – Feruloylquinic acid **(17, 18)**

R^1 = H, R^2 = OH – Coumaroylquinic acid **(19-22)**

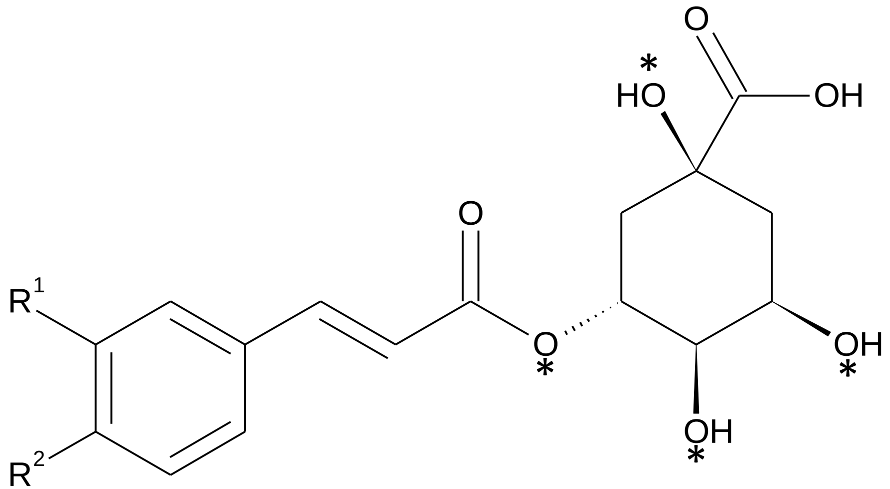

* indicates positions on which phenylpropenoic acid substitution is possible, depending on the compound, one or two positions are occupied

R = CH_3 – Hyperforin **(23)**

R = CH_2CH_3 – Adhyperforin **(24)**

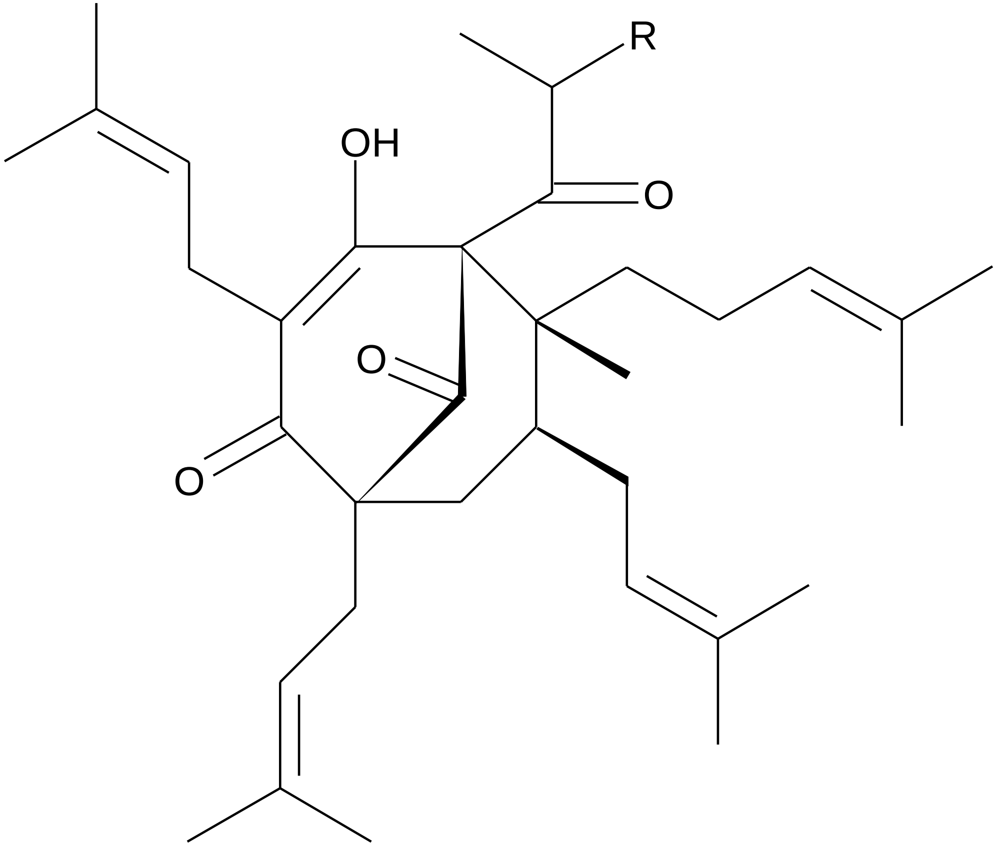

Furohyperforin **(25)**

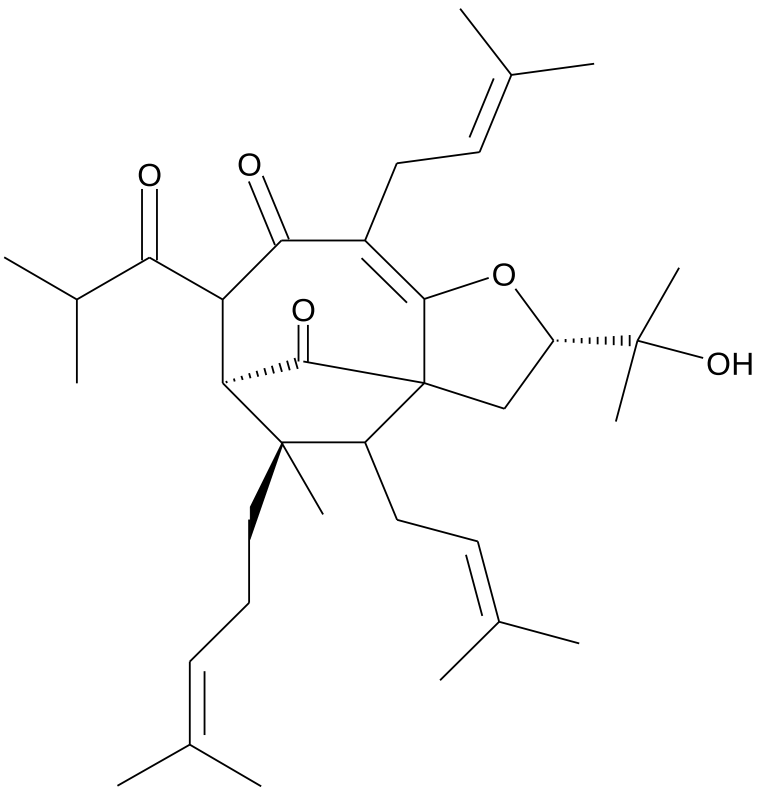

(25)

R = CH_3 – Hyperfirin **(26)**

R = CH_2CH_3 – Adhyperfirin **(27)**

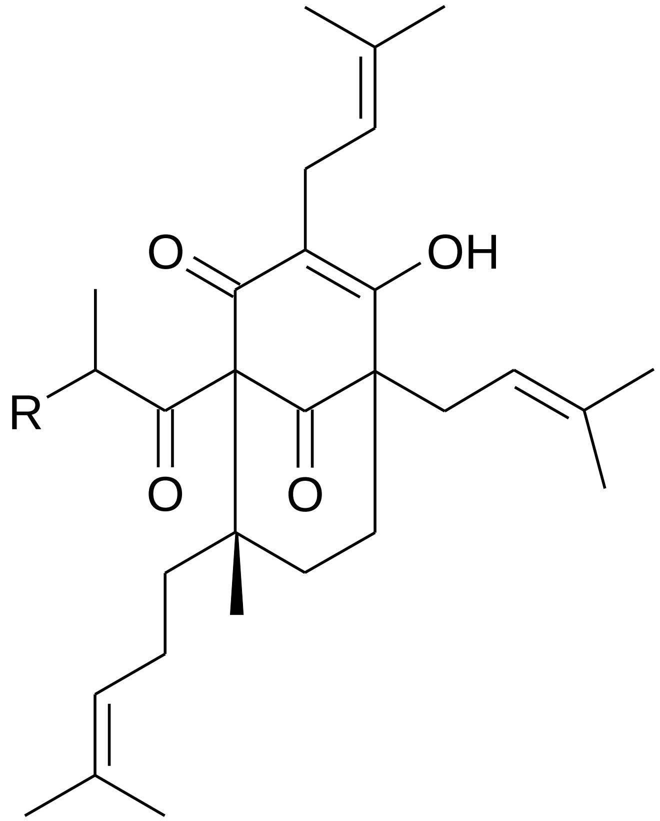

R_1, R_3 = H; R_2, R_4 = OH – Quercetin **(28)**
R_1, R_3 = H; R_2 = OH; R_4 = Rha – Quercitrin **(29)**
R_1, R_3 = H; R_2 = OH; R_4 = Gal – Hyperoside **(30)**
R_1, R_3 = H; R_2 = OH; R_4 = Rha-Glc – Rutin **(31)**

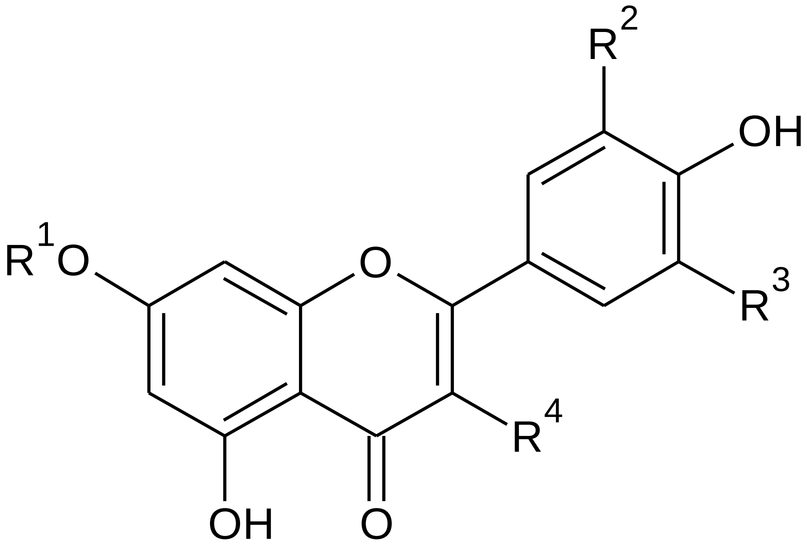

Mangiferin **(33)**

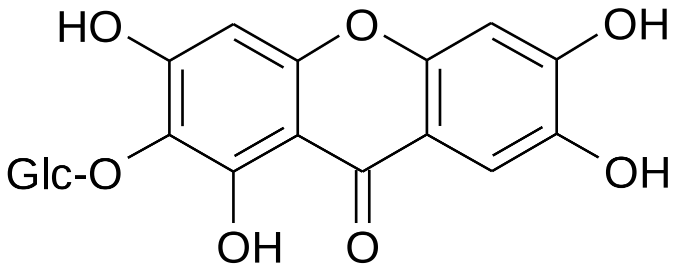

Fig. S2 Boxplots showing the relative abundances of the detected metabolites in individual Hypericum spp.

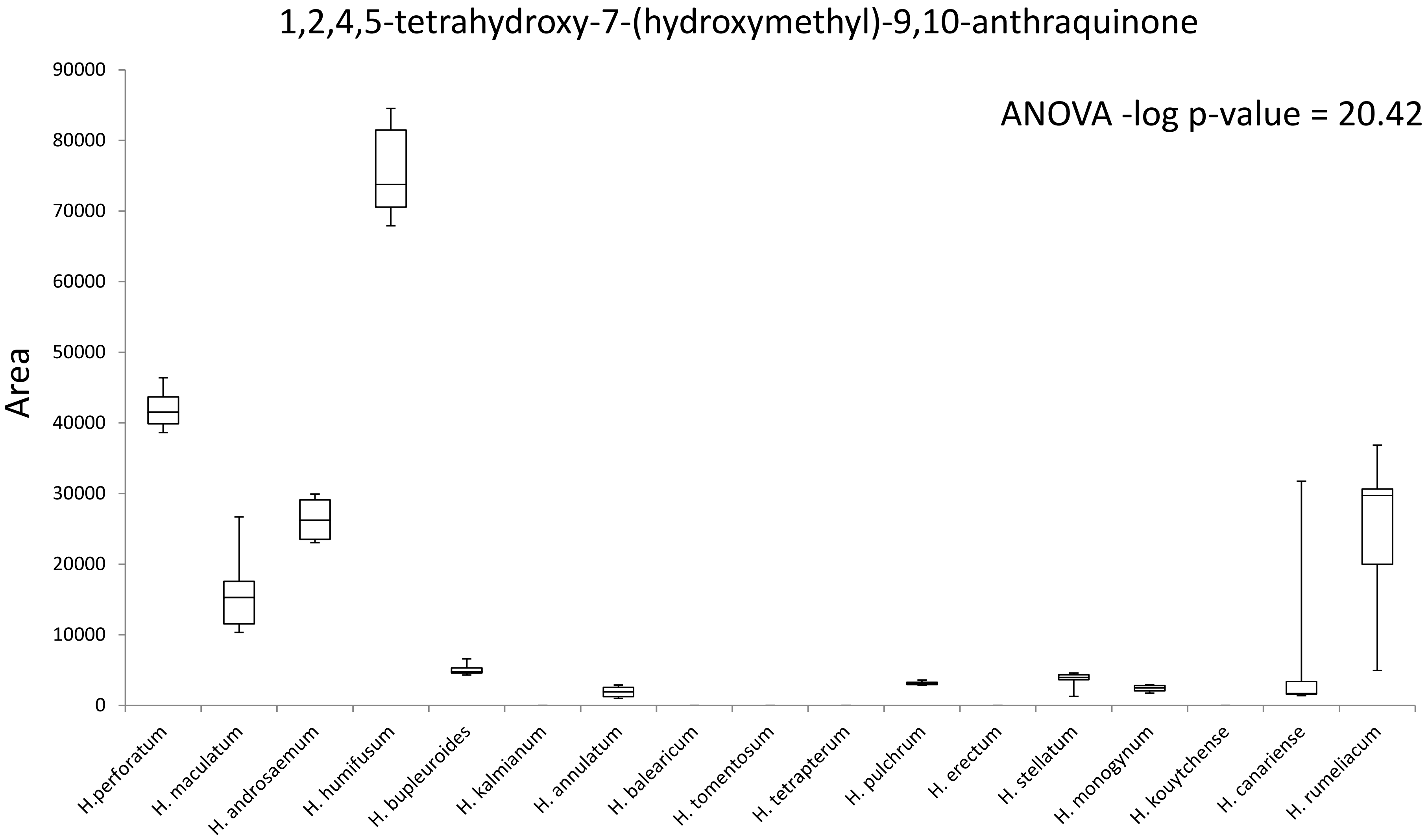

2

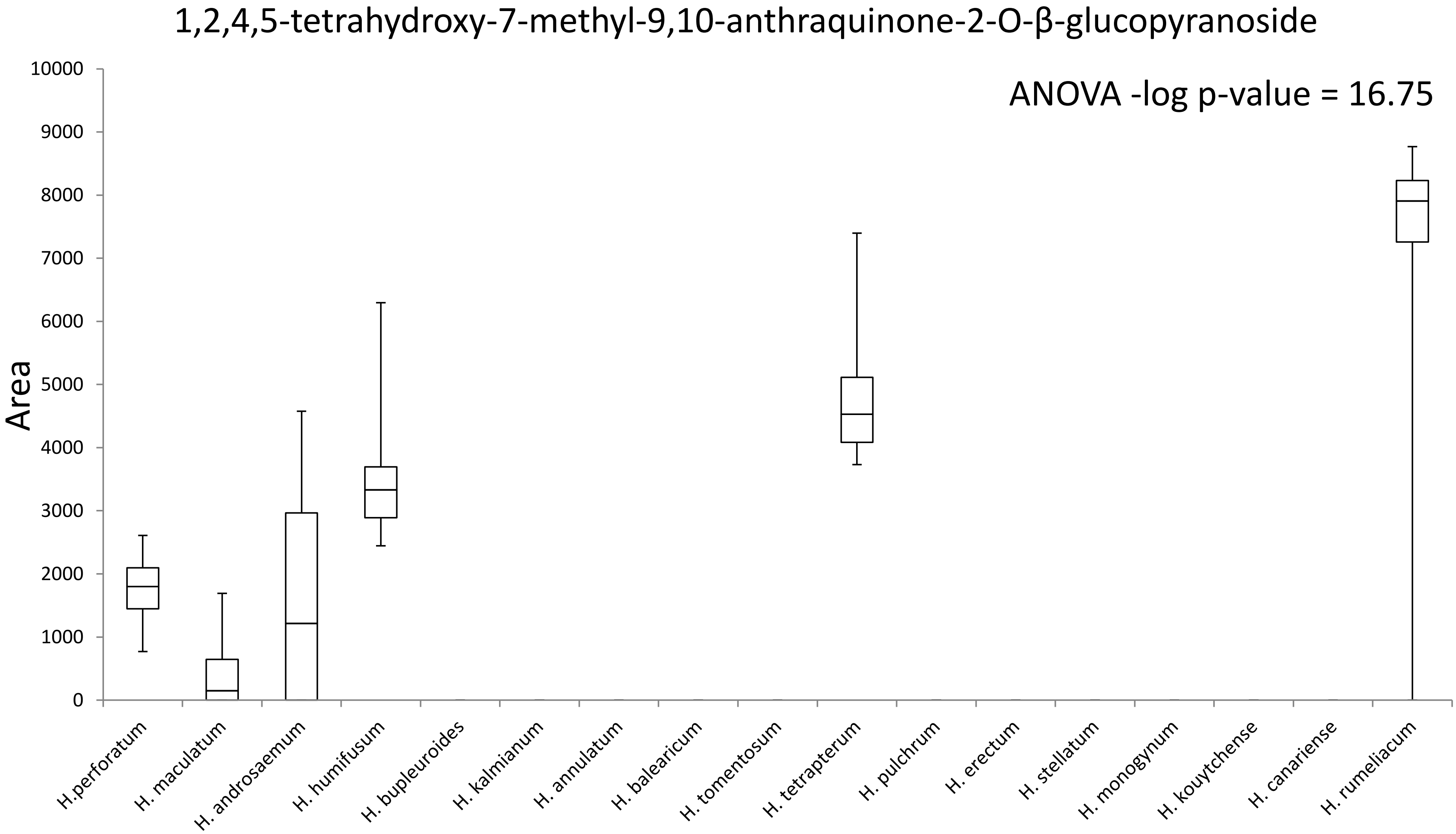
1,2,4,5-tetrahydroxy-7-methyl-9,10-anthraquinone-2-O-β-glucopyranoside
ANOVA -log p-value = 16.75
Area
0
1000
2000
3000
4000
5000
6000
7000
8000
9000
10000
H.perforatum
H. maculatum
H. androsaemum
H. humifusum
H. bupleuroides
H. kalmianum
H. annulatum
H. balearicum
H. tomentosum
H. tetrapterum
H. pulchrum
H. erectum
H. stellatum
H. monogynum
H. kouytchense
H. canariense
H. rumeliacum

3

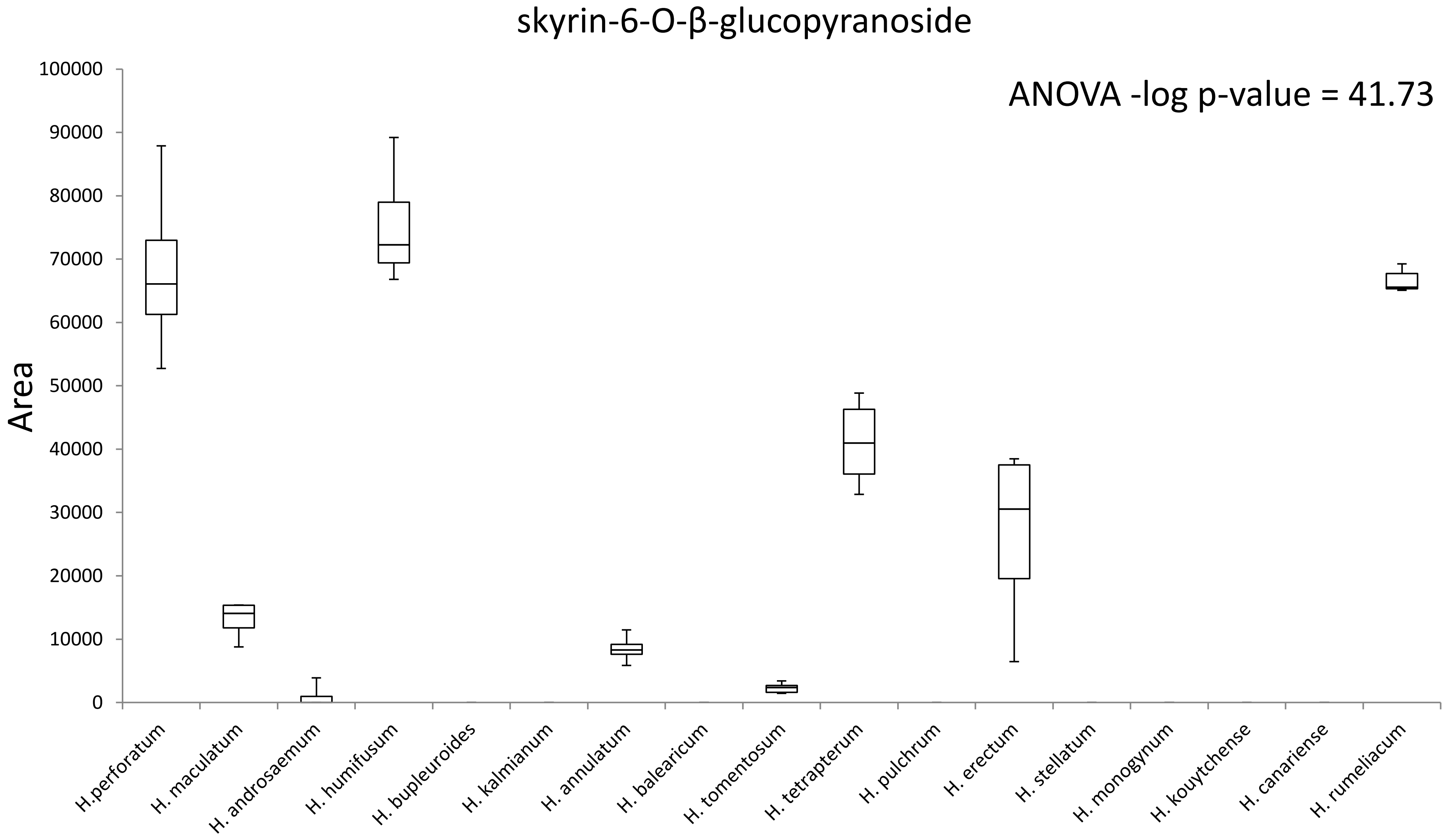
skyrin-6-O-β-glucopyranoside
ANOVA -log p-value = 41.73
Area
100000
90000
80000
70000
60000
50000
40000
30000
20000
10000
0
H.perforatum
H. maculatum
H. androsaemum
H. humifusum
H. bupleuroides
H. kalmianum
H. annulatum
H. balearicum
H. tomentosum
H. tetrapterum
H. pulchrum
H. erectum
H. stellatum
H. monogynum
H. kouytchense
H. canariense
H. rumeliacum

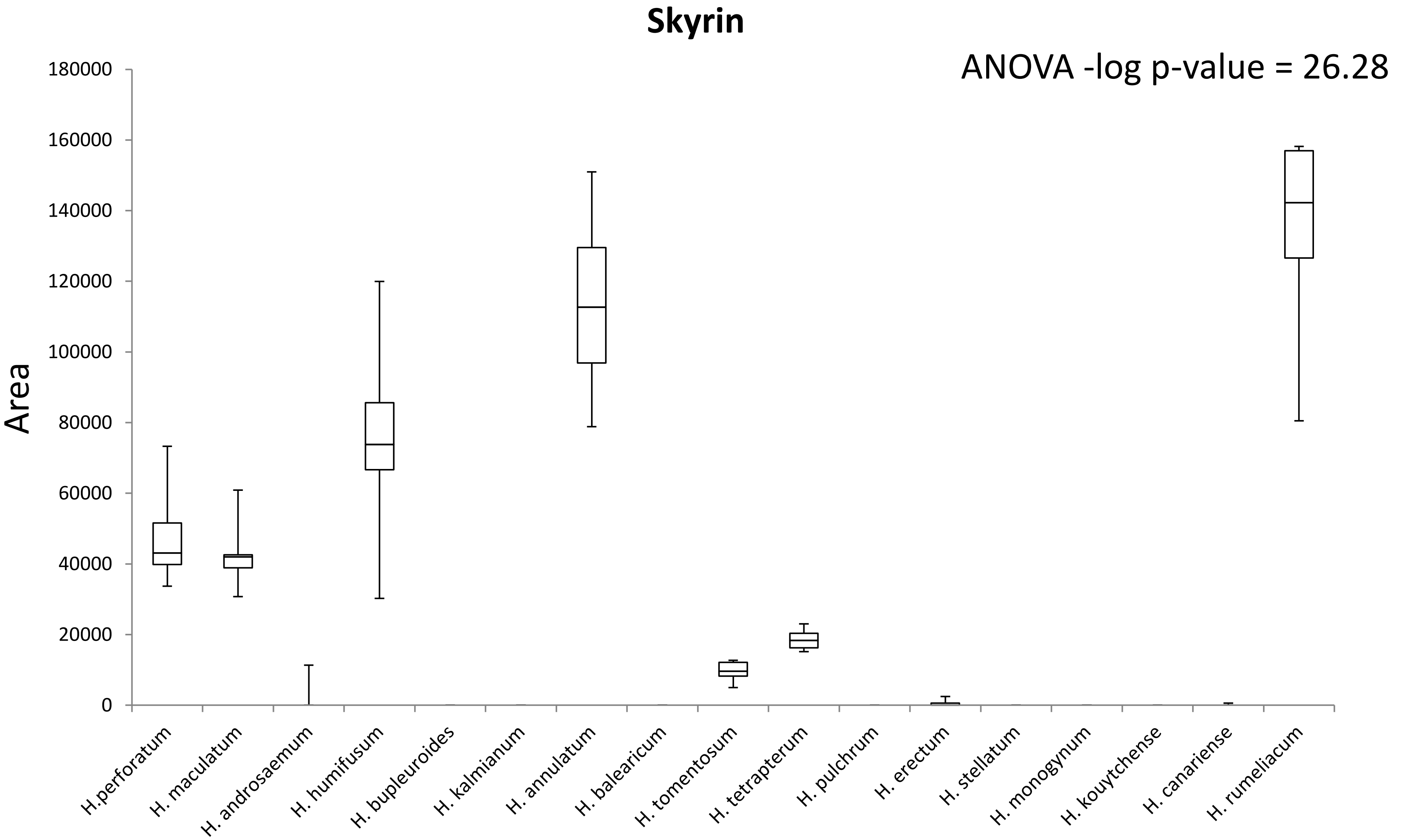
Skyrin
ANOVA -log p-value = 26.28
Area
180000
160000
140000
120000
100000
80000
60000
40000
20000
0
H.perforatum
H. maculatum
H. androsaemum
H. humifusum
H. bupleuroides
H. kalmianum
H. annulatum
H. balearicum
H. tomentosum
H. tetrapterum
H. pulchrum
H. erectum
H. stellatum
H. monogynum
H. kouytchense
H. canariense
H. rumeliacum

5

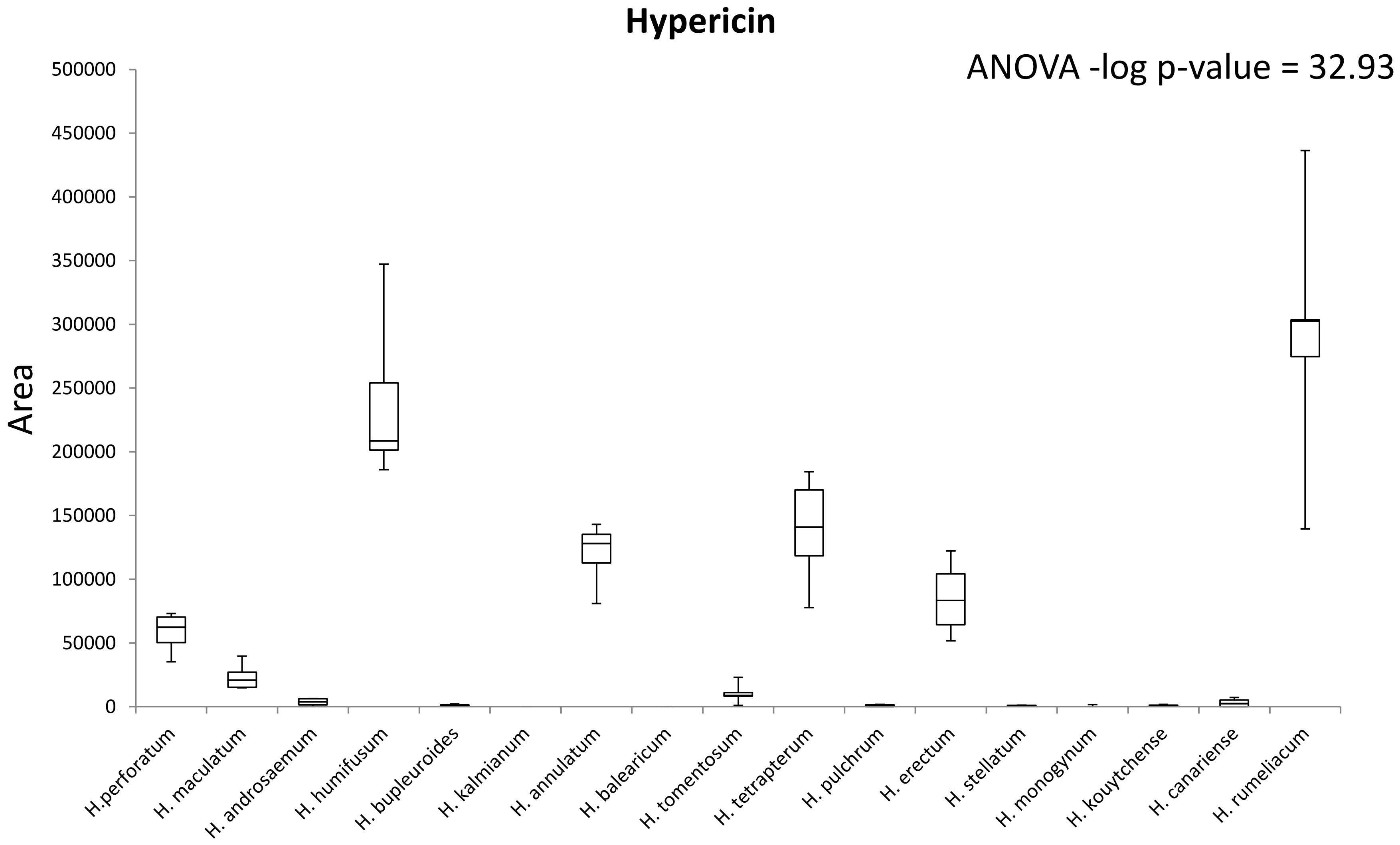
Hypericin
ANOVA -log p-value = 32.93
Area
500000
450000
400000
350000
300000
250000
200000
150000
100000
50000
0
H.perforatum
H. maculatum
H. androsaemum
H. humifusum
H. bupleuroides
H. kalmianum
H. annulatum
H. balearicum
H. tomentosum
H. tetrapterum
H. pulchrum
H. erectum
H. stellatum
H. monogynum
H. kouytchense
H. canariense
H. rumeliacum

6

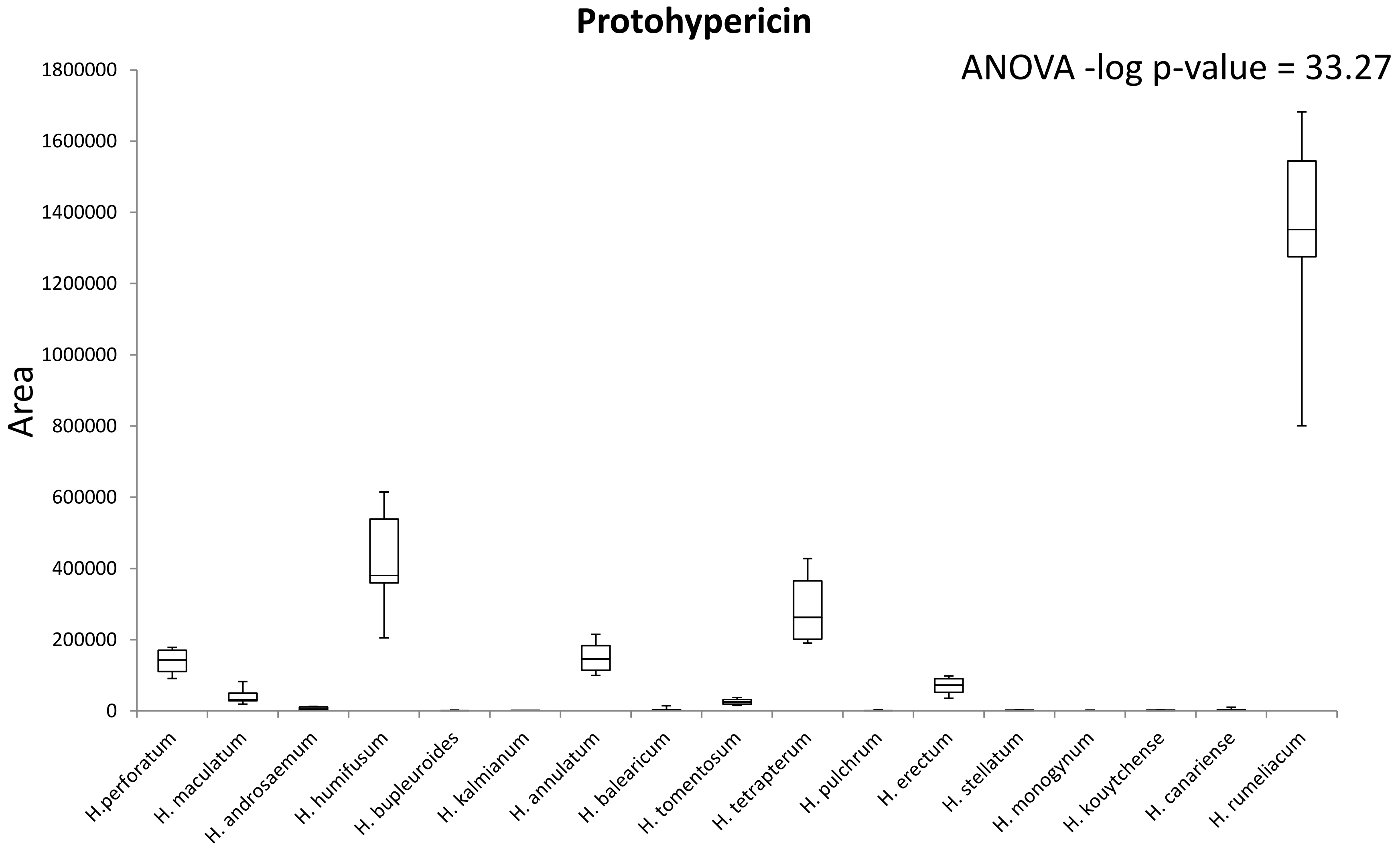

Protohypericin
ANOVA -log p-value = 33.27
Area
1800000
1600000
1400000
1200000
1000000
800000
600000
400000
200000
0
H.perforatum
H. maculatum
H. androsaemum
H. humifusum
H. bupleuroides
H. kalmianum
H. annulatum
H. balearicum
H. tomentosum
H. tetrapterum
H. pulchrum
H. erectum
H. stellatum
H. monogynum
H. kouytchense
H. canariense
H. rumeliacum

7

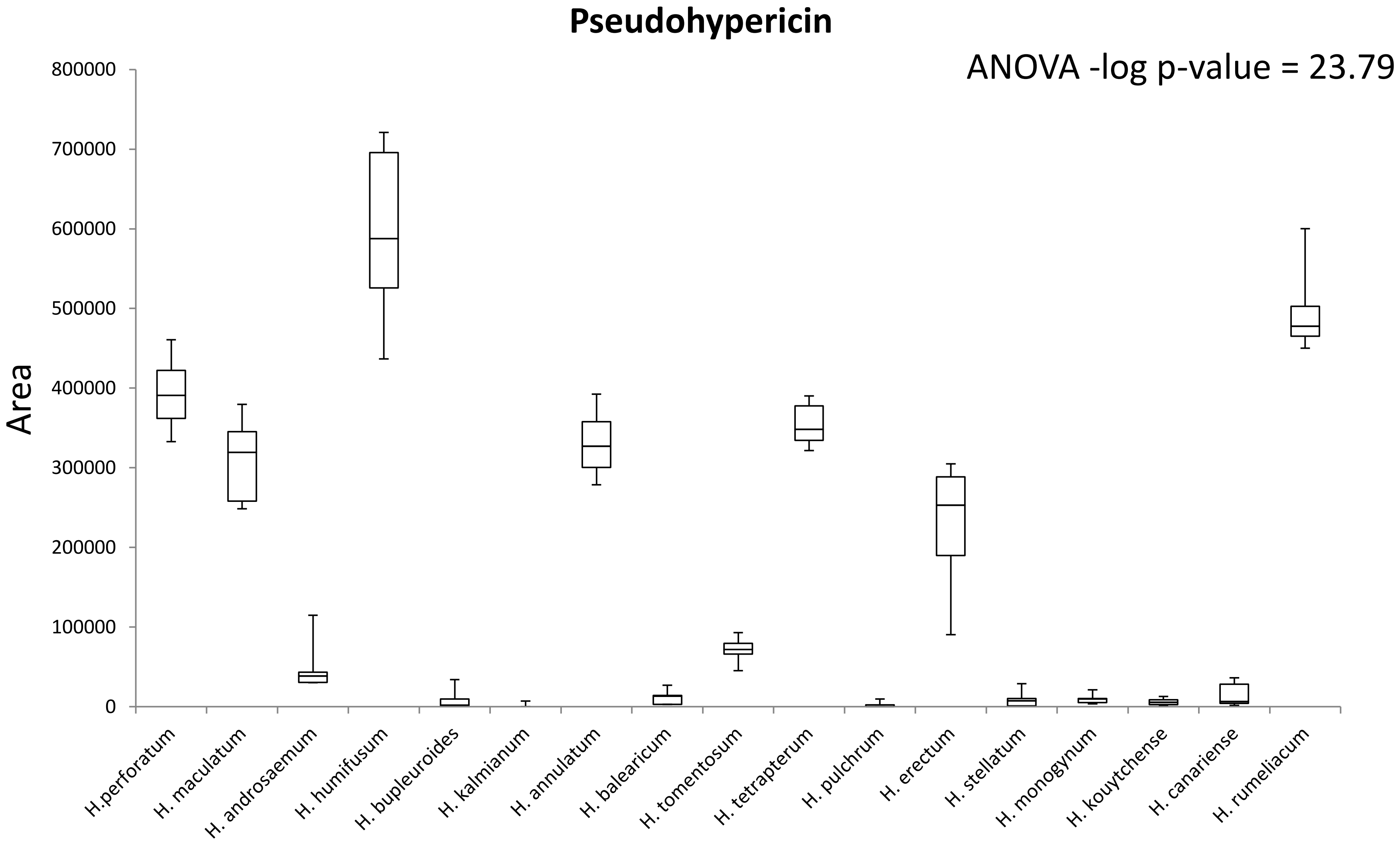
Pseudohypericin
ANOVA -log p-value = 23.79
Area
800000
700000
600000
500000
400000
300000
200000
100000
0
H.perforatum
H. maculatum
H. androsaemum
H. humifusum
H. bupleuroides
H. kalmianum
H. annulatum
H. balearicum
H. tomentosum
H. tetrapterum
H. pulchrum
H. erectum
H. stellatum
H. monogynum
H. kouytchense
H. canariense
H. rumeliacum

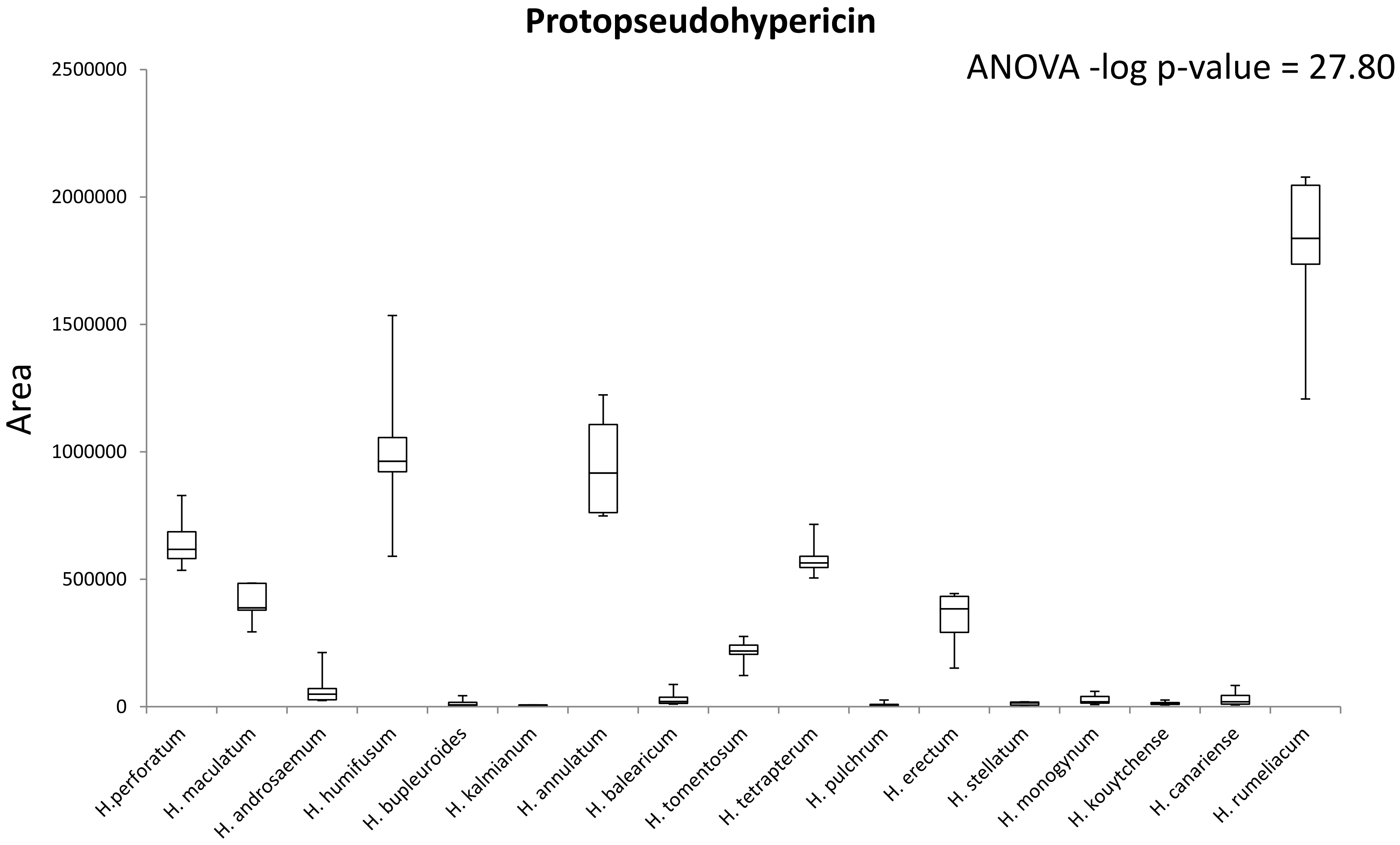
Protopseudohypericin
ANOVA -log p-value = 27.80
Area
2500000
2000000
1500000
1000000
500000
0
H.perforatum
H. maculatum
H. androsaemum
H. humifusum
H. bupleuroides
H. kalmianum
H. annulatum
H. balearicum
H. tomentosum
H. tetrapterum
H. pulchrum
H. erectum
H. stellatum
H. monogynum
H. kouytchense
H. canariense
H. rumeliacum

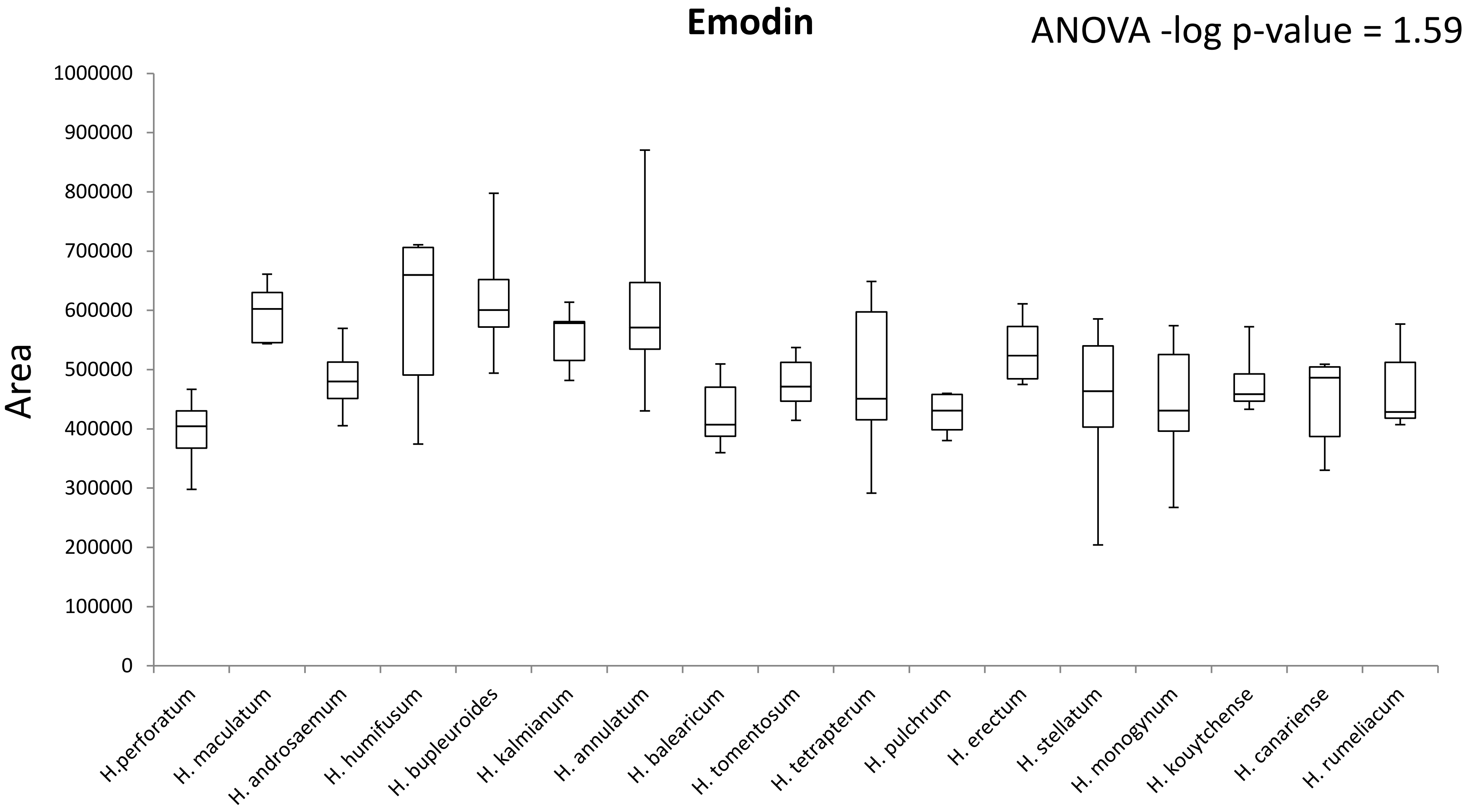
Emodin
ANOVA -log p-value = 1.59
Area
1000000
900000
800000
700000
600000
500000
400000
300000
200000
100000
0
H.perforatum
H. maculatum
H. androsaemum
H. humifusum
H. bupleuroides
H. kalmianum
H. annulatum
H. balearicum
H. tomentosum
H. tetrapterum
H. pulchrum
H. erectum
H. stellatum
H. monogynum
H. kouytchense
H. canariense
H. rumeliacum

10

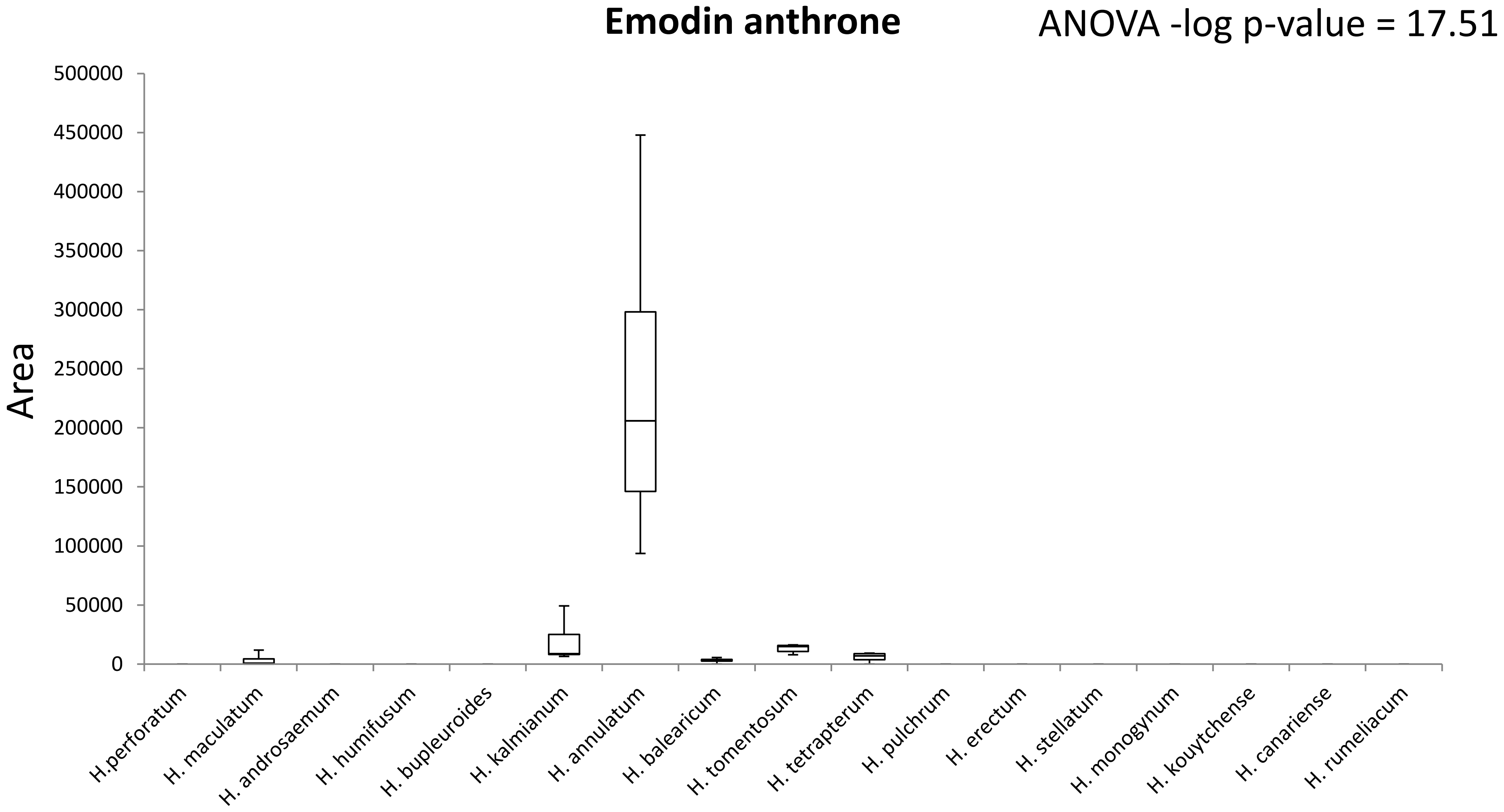

Emodin anthrone
ANOVA -log p-value = 17.51
Area
500000
450000
400000
350000
300000
250000
200000
150000
100000
50000
0
H.perforatum
H. maculatum
H. androsaemum
H. humifusum
H. bupleuroides
H. kalmianum
H. annulatum
H. balearicum
H. tomentosum
H. tetrapterum
H. pulchrum
H. erectum
H. stellatum
H. monogynum
H. kouytchense
H. canariense
H. rumeliacum

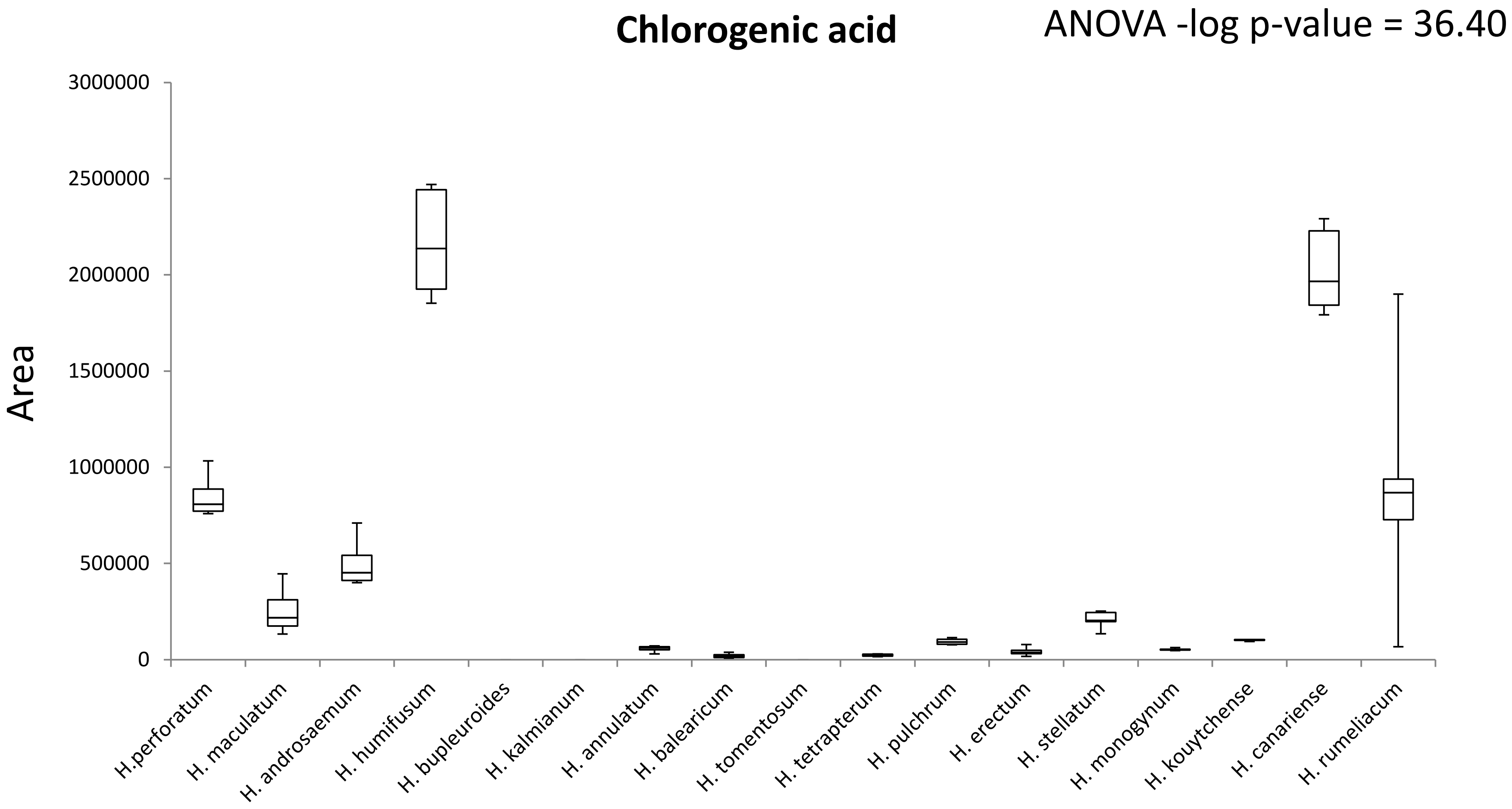

Chlorogenic acid
ANOVA -log p-value = 36.40
Area
3000000
2500000
2000000
1500000
1000000
500000
0
H.perforatum
H. maculatum
H. androsaemum
H. humifusum
H. bupleuroides
H. kalmianum
H. annulatum
H. balearicum
H. tomentosum
H. tetrapterum
H. pulchrum
H. erectum
H. stellatum
H. monogynum
H. kouytchense
H. canariense
H. rumeliacum

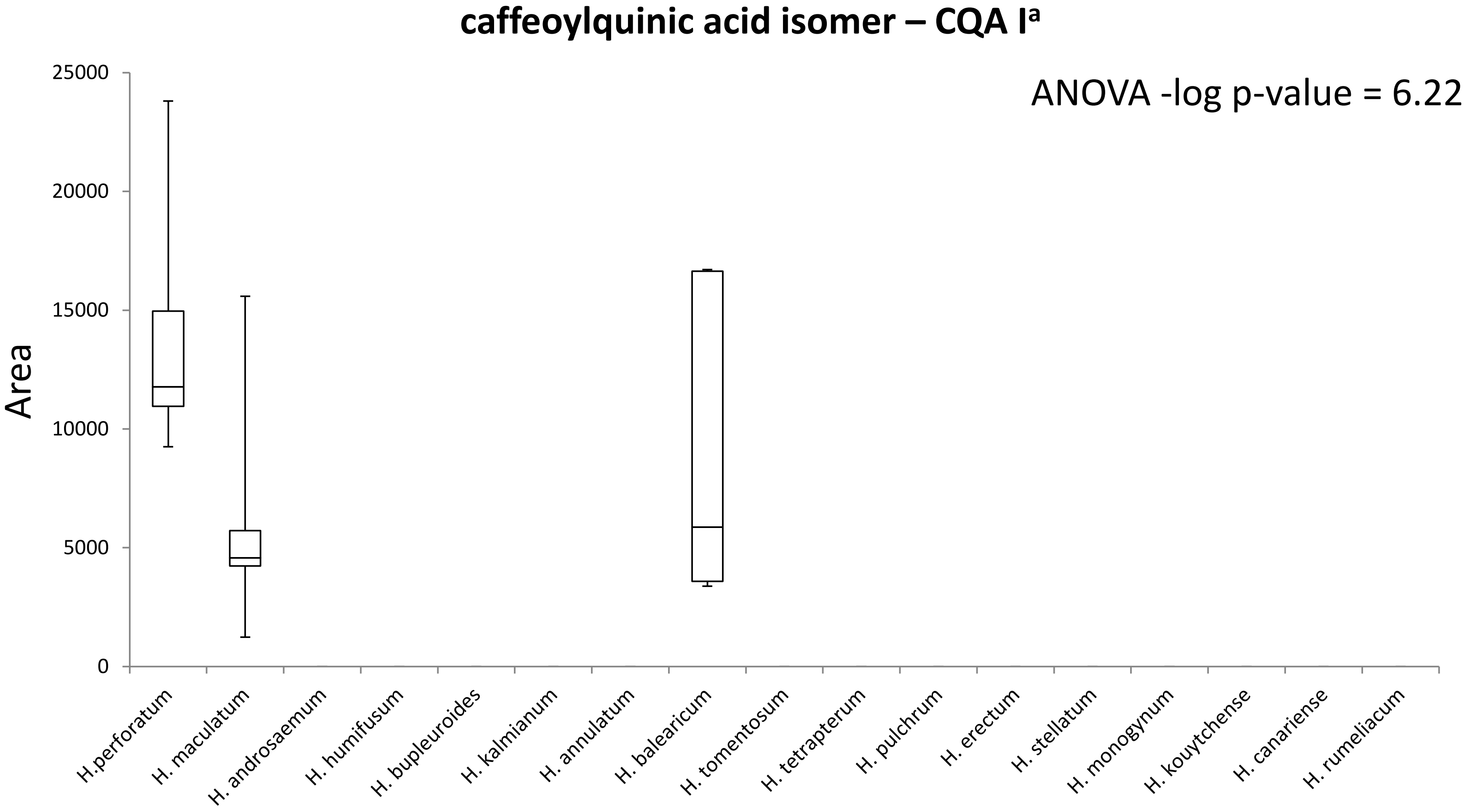
caffeoylquinic acid isomer – CQA I[a]
ANOVA -log p-value = 6.22
Area
25000
20000
15000
10000
5000
0
H.perforatum
H. maculatum
H. androsaemum
H. humifusum
H. bupleuroides
H. kalmianum
H. annulatum
H. balearicum
H. tomentosum
H. tetrapterum
H. pulchrum
H. erectum
H. stellatum
H. monogynum
H. kouytchense
H. canariense
H. rumeliacum

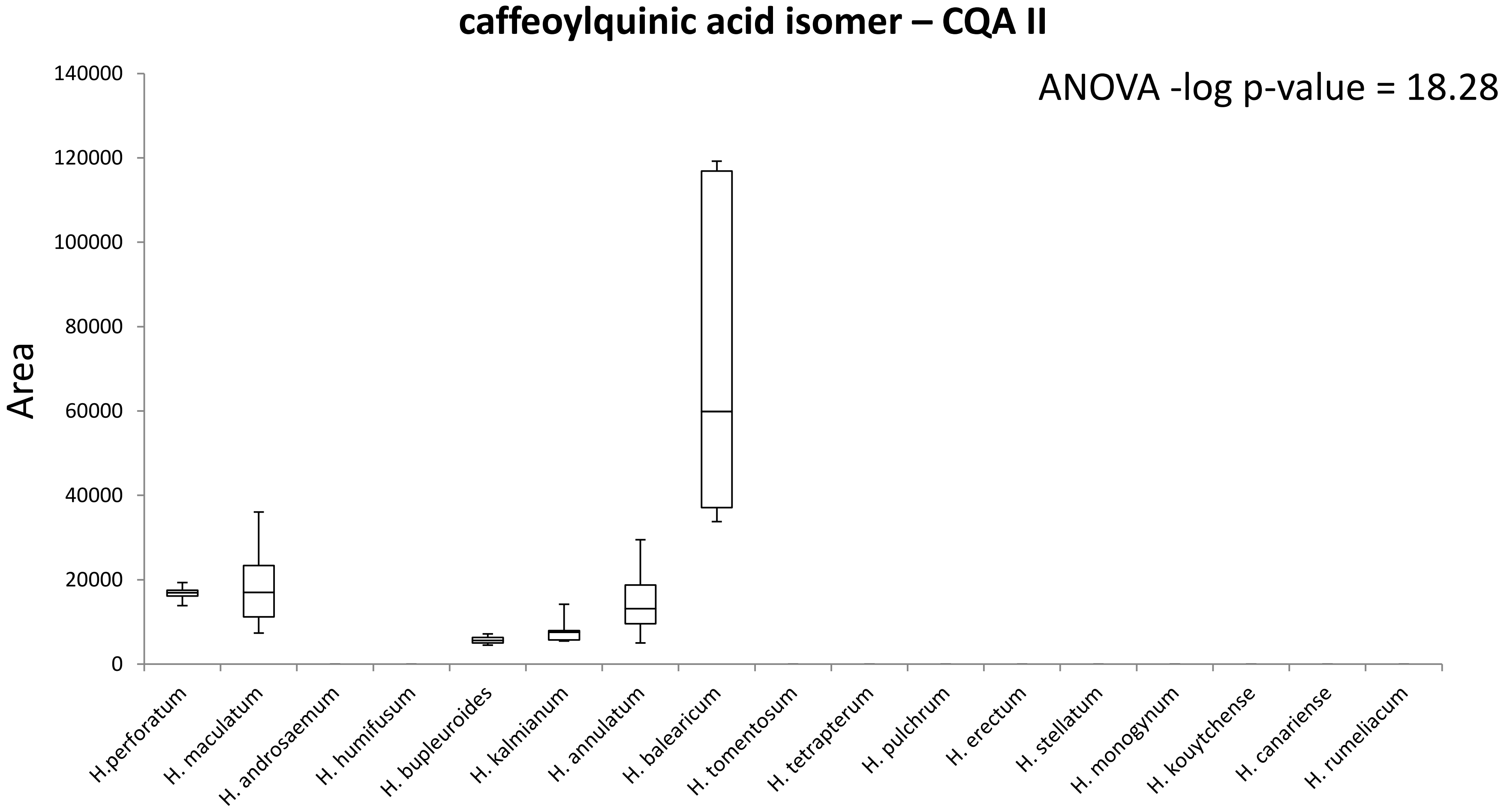
caffeoylquinic acid isomer – CQA II
ANOVA -log p-value = 18.28
Area
140000
120000
100000
80000
60000
40000
20000
0
H.perforatum
H. maculatum
H. androsaemum
H. humifusum
H. bupleuroides
H. kalmianum
H. annulatum
H. balearicum
H. tomentosum
H. tetrapterum
H. pulchrum
H. erectum
H. stellatum
H. monogynum
H. kouytchense
H. canariense
H. rumeliacum

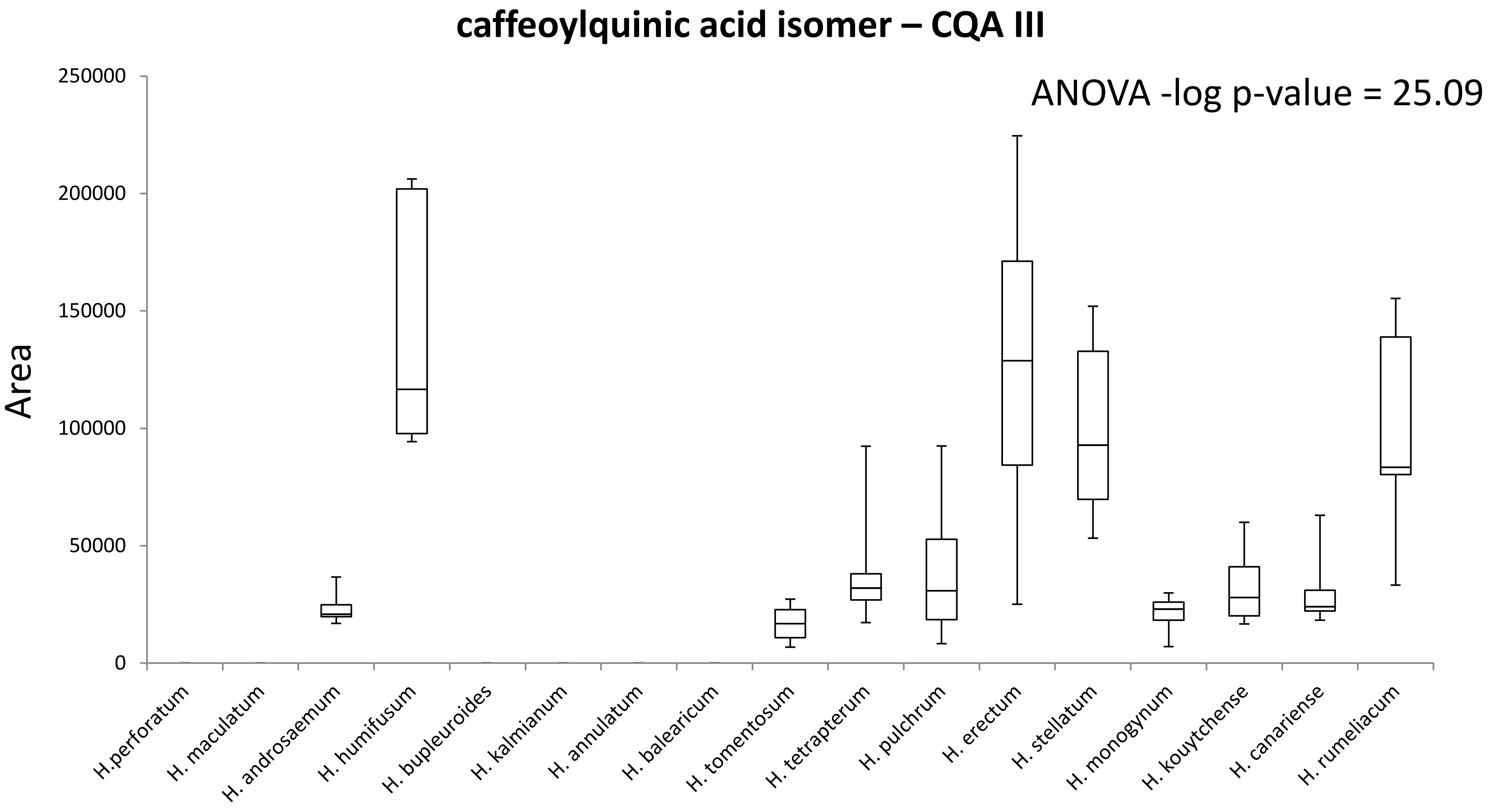
caffeoylquinic acid isomer – CQA III
ANOVA -log p-value = 25.09
Area
250000
200000
150000
100000
50000
0
H.perforatum
H. maculatum
H. androsaemum
H. humifusum
H. bupleuroides
H. kalmianum
H. annulatum
H. balearicum
H. tomentosum
H. tetrapterum
H. pulchrum
H. erectum
H. stellatum
H. monogynum
H. kouytchense
H. canariense
H. rumeliacum

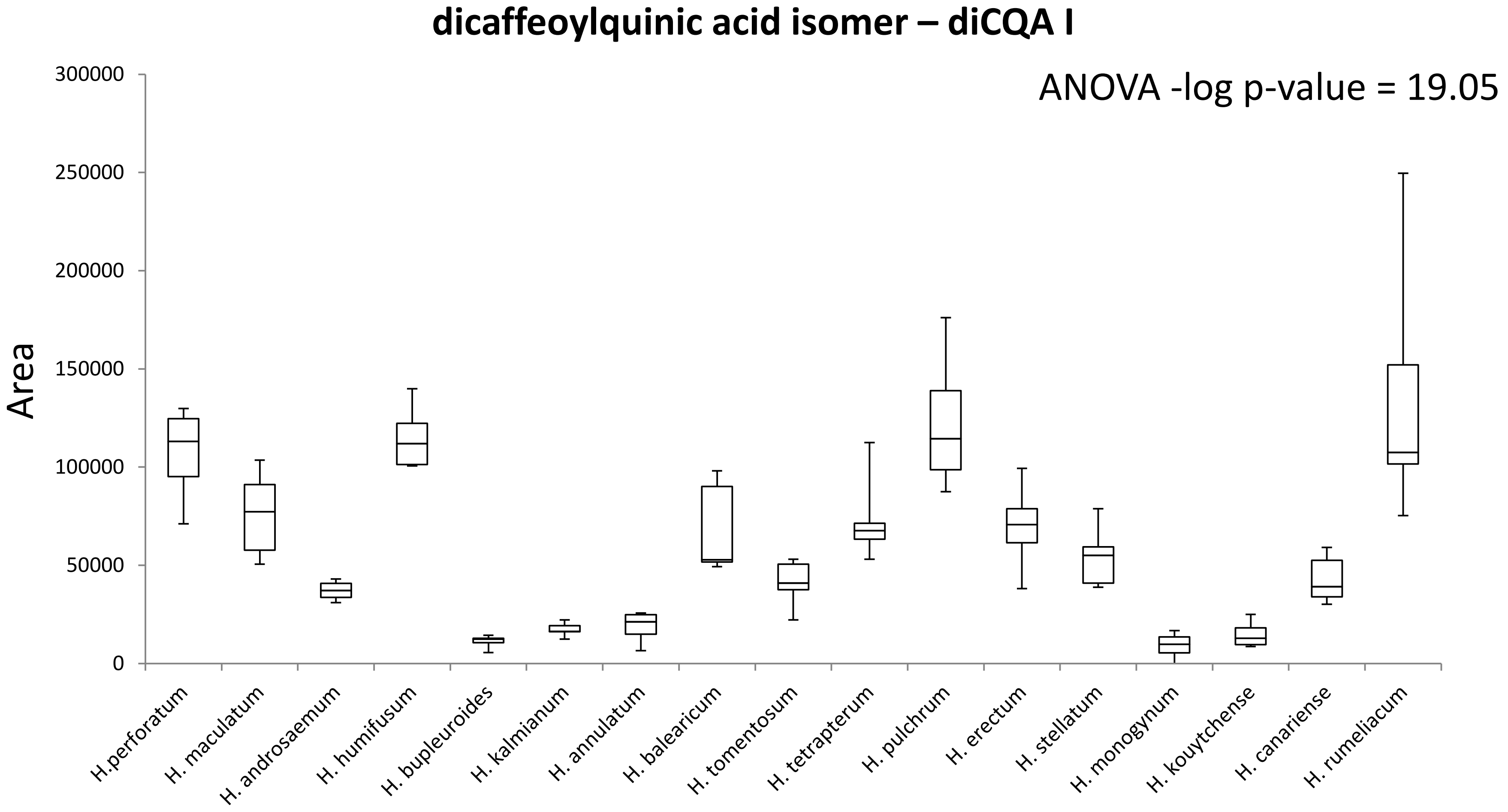
dicaffeoylquinic acid isomer – diCQA I
ANOVA -log p-value = 19.05
Area
300000
250000
200000
150000
100000
50000
0
H.perforatum
H. maculatum
H. androsaemum
H. humifusum
H. bupleuroides
H. kalmianum
H. annulatum
H. balearicum
H. tomentosum
H. tetrapterum
H. pulchrum
H. erectum
H. stellatum
H. monogynum
H. kouytchense
H. canariense
H. rumeliacum

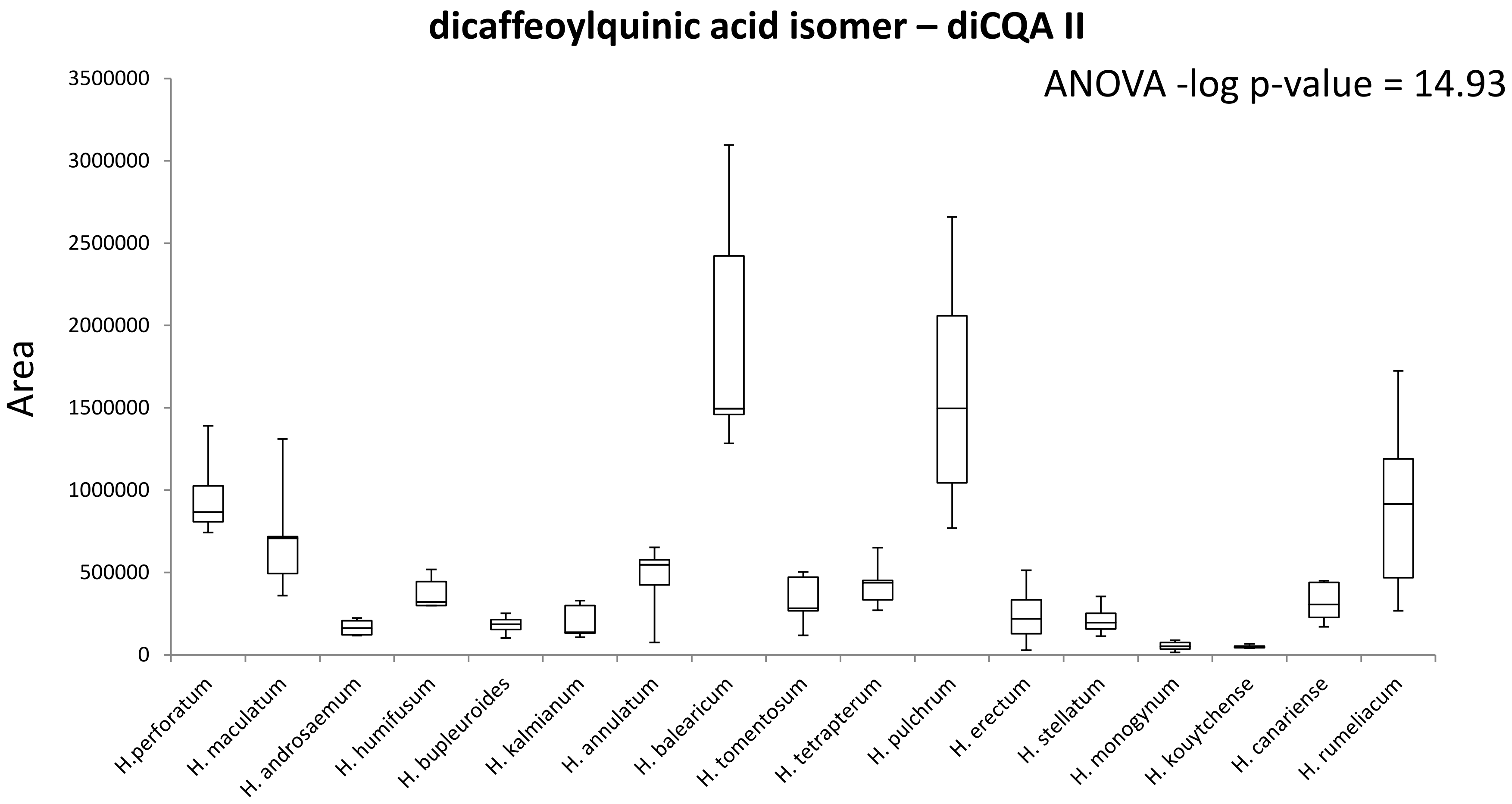

dicaffeoylquinic acid isomer – diCQA II
ANOVA -log p-value = 14.93
Area
3500000
3000000
2500000
2000000
1500000
1000000
500000
0
H.perforatum
H. maculatum
H. androsaemum
H. humifusum
H. bupleuroides
H. kalmianum
H. annulatum
H. balearicum
H. tomentosum
H. tetrapterum
H. pulchrum
H. erectum
H. stellatum
H. monogynum
H. kouytchense
H. canariense
H. rumeliacum

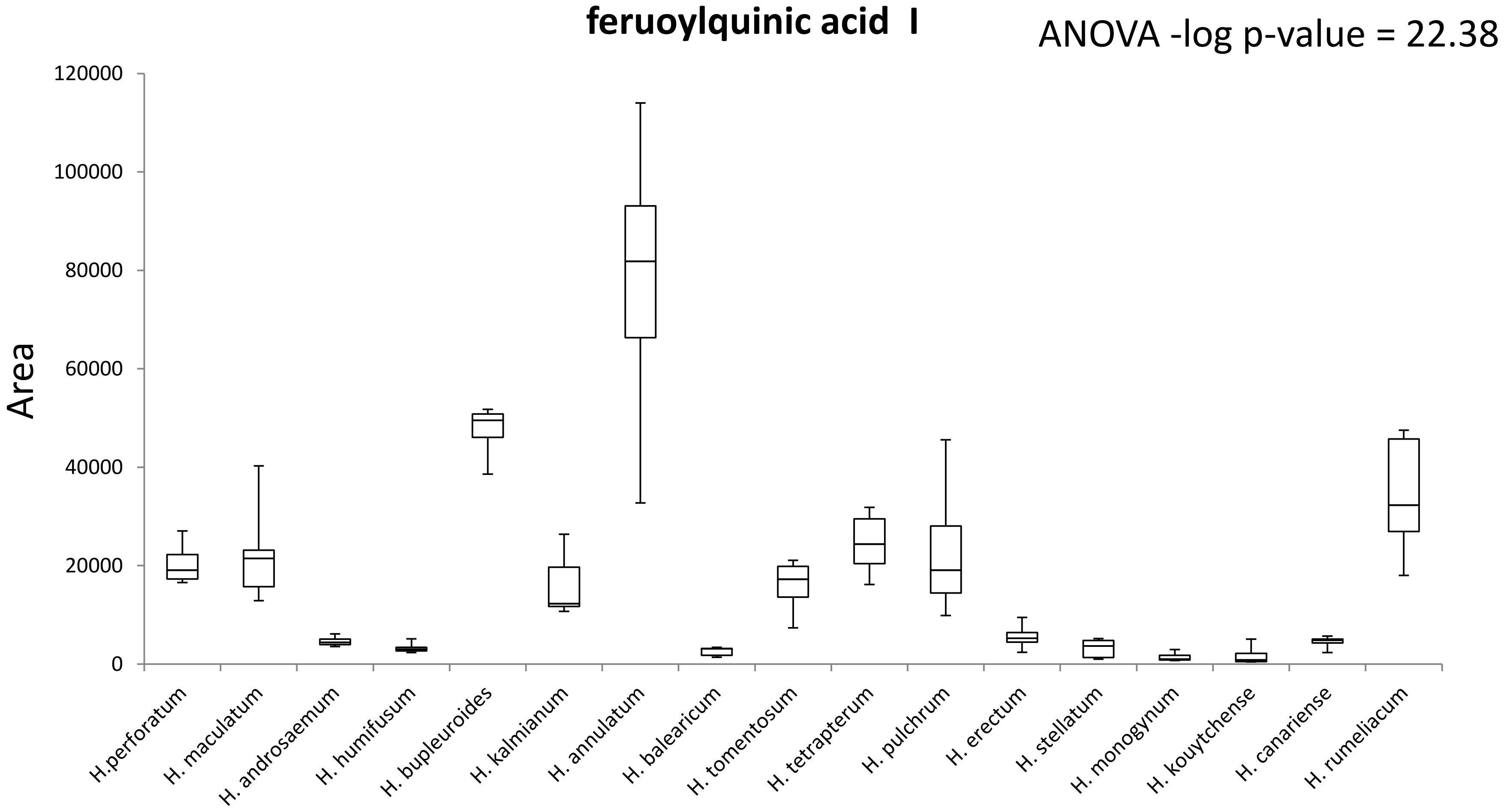
feruoylquinic acid I
ANOVA -log p-value = 22.38
Area
120000
100000
80000
60000
40000
20000
0
H.perforatum
H. maculatum
H. androsaemum
H. humifusum
H. bupleuroides
H. kalmianum
H. annulatum
H. balearicum
H. tomentosum
H. tetrapterum
H. pulchrum
H. erectum
H. stellatum
H. monogynum
H. kouytchense
H. canariense
H. rumeliacum

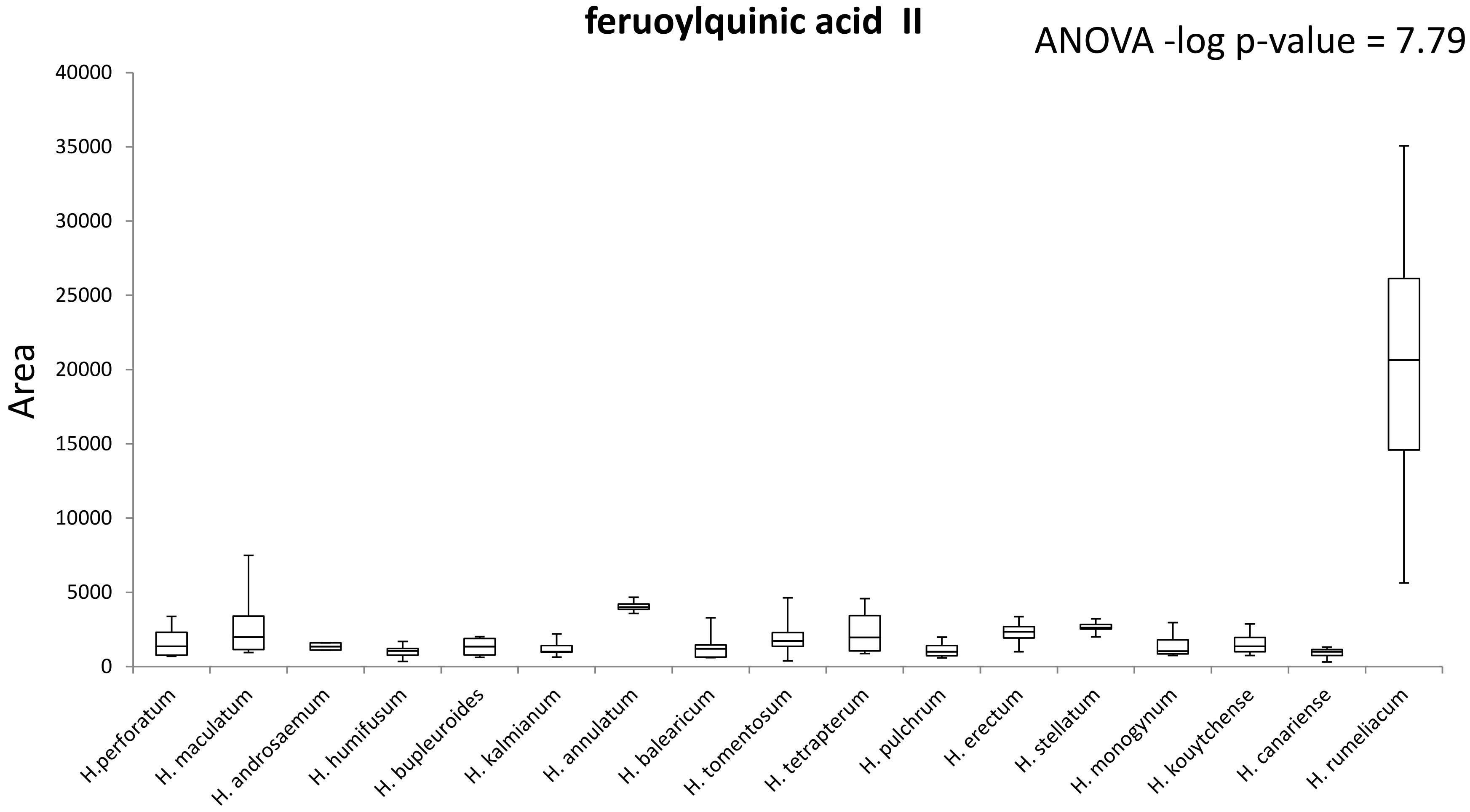

feruoylquinic acid II
ANOVA -log p-value = 7.79
Area
40000
35000
30000
25000
20000
15000
10000
5000
0
H.perforatum
H. maculatum
H. androsaemum
H. humifusum
H. bupleuroides
H. kalmianum
H. annulatum
H. balearicum
H. tomentosum
H. tetrapterum
H. pulchrum
H. erectum
H. stellatum
H. monogynum
H. kouytchense
H. canariense
H. rumeliacum

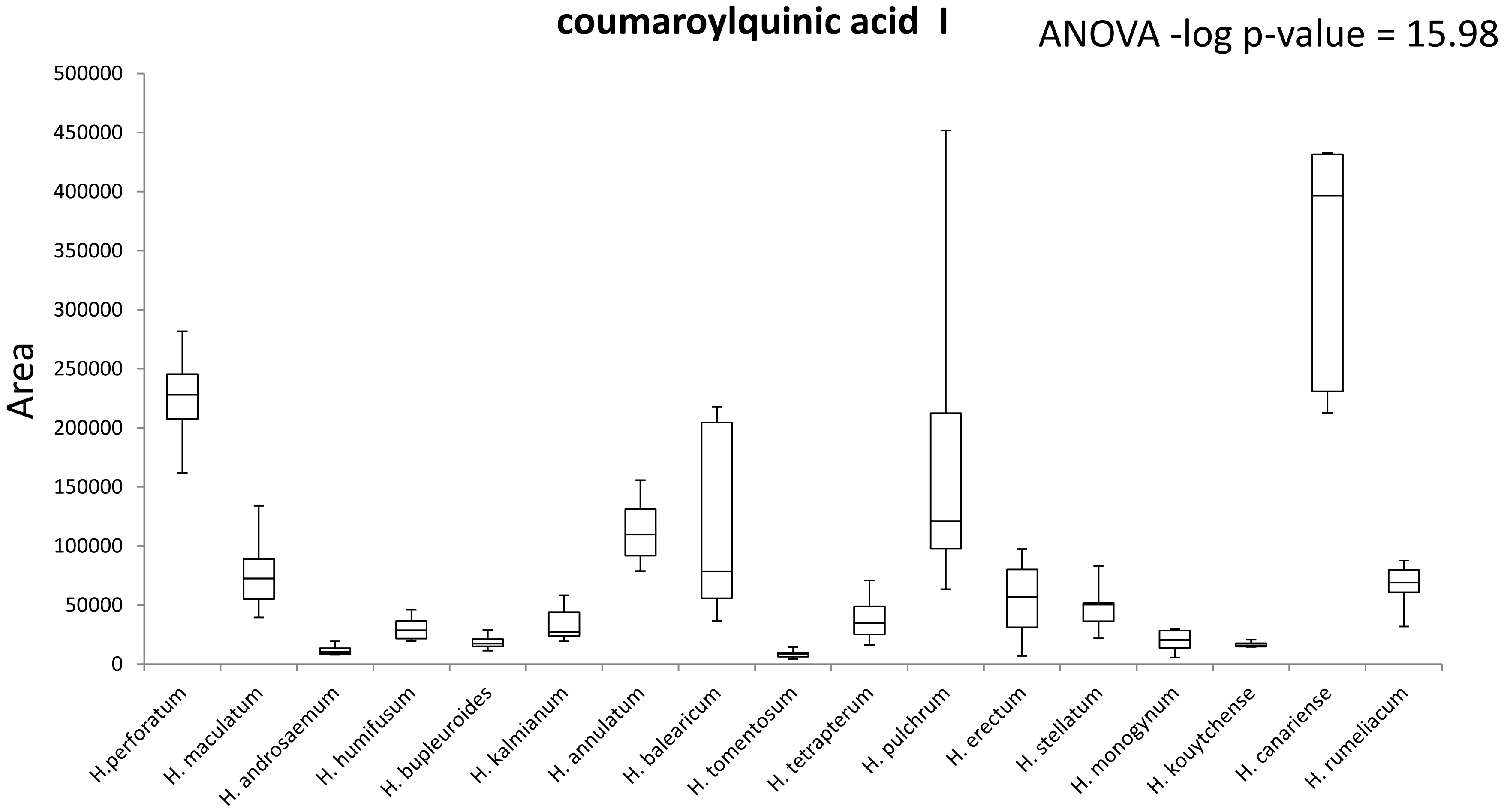
coumaroylquinic acid I
ANOVA -log p-value = 15.98
Area
500000
450000
400000
350000
300000
250000
200000
150000
100000
50000
0
H.perforatum
H. maculatum
H. androsaemum
H. humifusum
H. bupleuroides
H. kalmianum
H. annulatum
H. balearicum
H. tomentosum
H. tetrapterum
H. pulchrum
H. erectum
H. stellatum
H. monogynum
H. kouytchense
H. canariense
H. rumeliacum

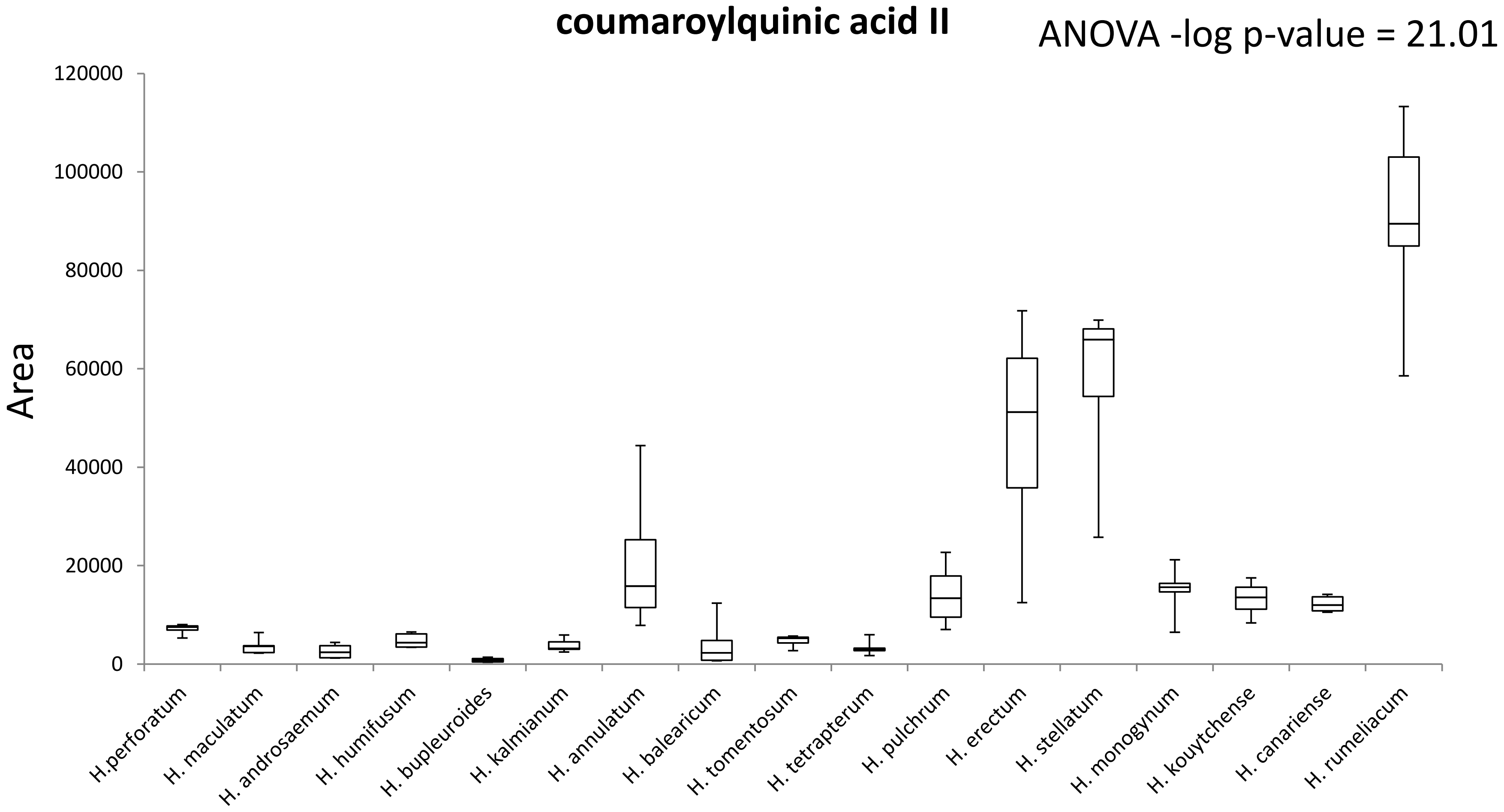
coumaroylquinic acid II
ANOVA -log p-value = 21.01
Area
120000
100000
80000
60000
40000
20000
0
H.perforatum
H. maculatum
H. androsaemum
H. humifusum
H. bupleuroides
H. kalmianum
H. annulatum
H. balearicum
H. tomentosum
H. tetrapterum
H. pulchrum
H. erectum
H. stellatum
H. monogynum
H. kouytchense
H. canariense
H. rumeliacum

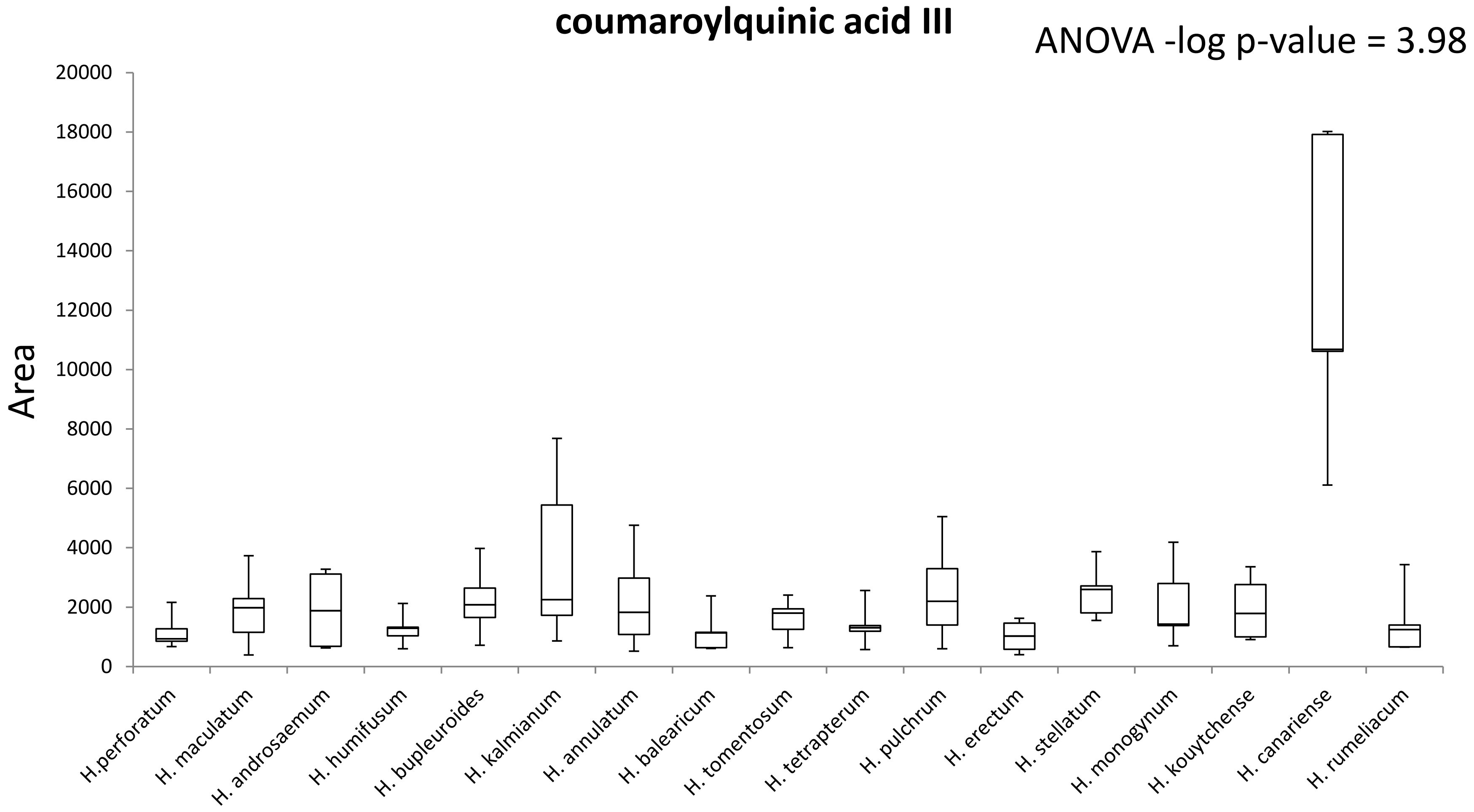
coumaroylquinic acid III
ANOVA -log p-value = 3.98
Area
20000
18000
16000
14000
12000
10000
8000
6000
4000
2000
0
H.perforatum
H. maculatum
H. androsaemum
H. humifusum
H. bupleuroides
H. kalmianum
H. annulatum
H. balearicum
H. tomentosum
H. tetrapterum
H. pulchrum
H. erectum
H. stellatum
H. monogynum
H. kouytchense
H. canariense
H. rumeliacum

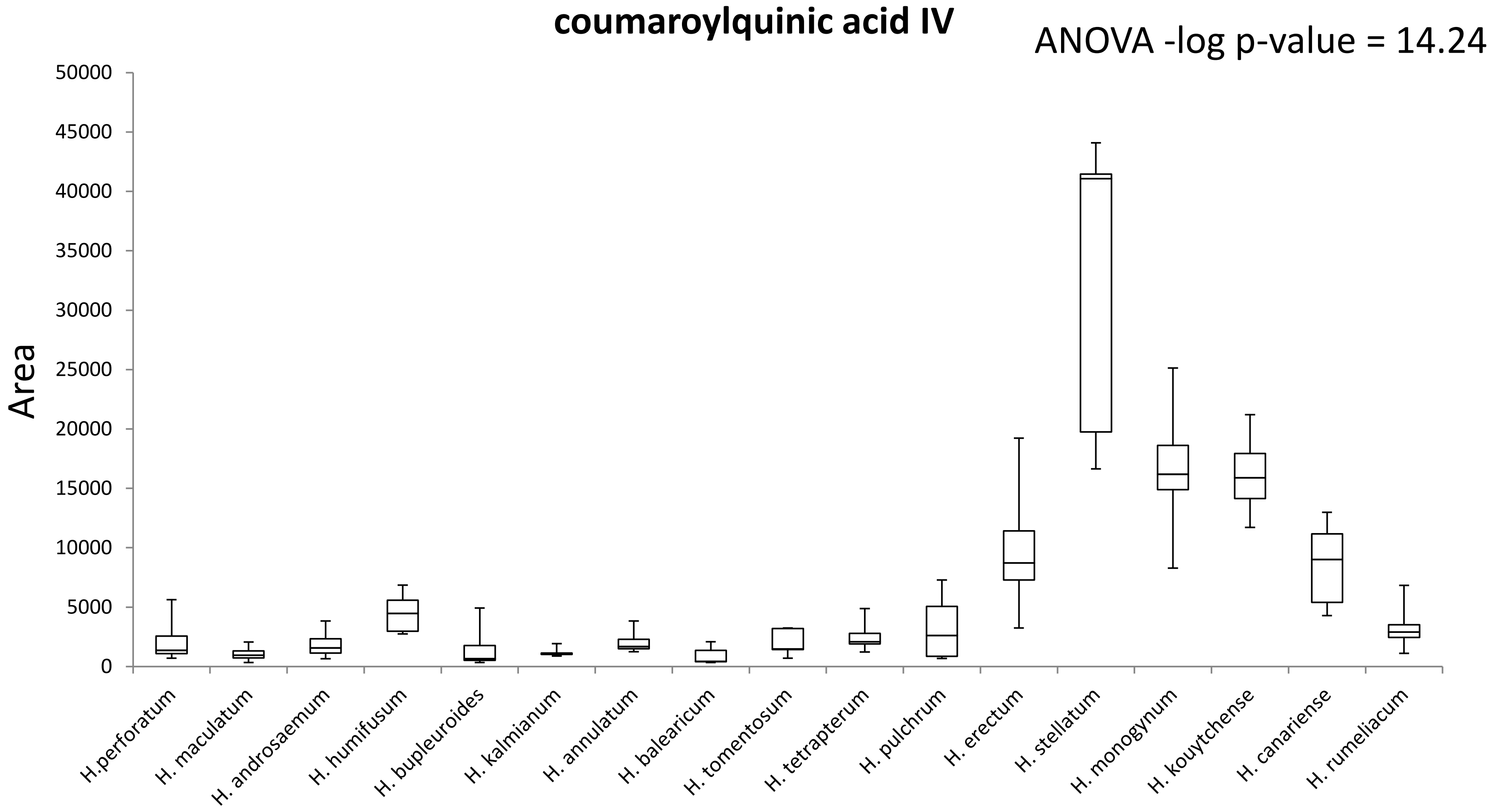
coumaroylquinic acid IV
ANOVA -log p-value = 14.24
Area
50000
45000
40000
35000
30000
25000
20000
15000
10000
5000
0
H.perforatum
H. maculatum
H. androsaemum
H. humifusum
H. bupleuroides
H. kalmianum
H. annulatum
H. balearicum
H. tomentosum
H. tetrapterum
H. pulchrum
H. erectum
H. stellatum
H. monogynum
H. kouytchense
H. canariense
H. rumeliacum

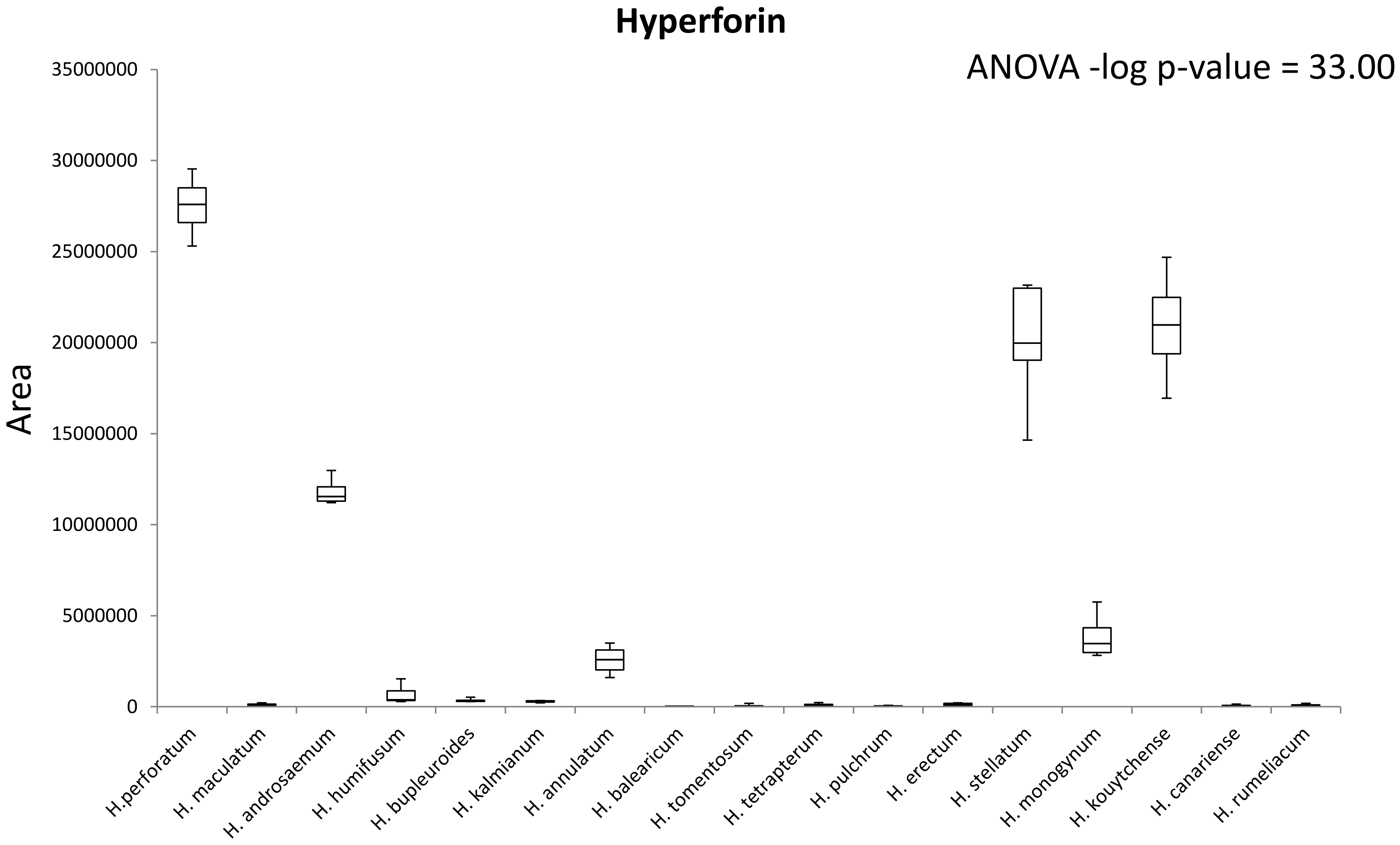
Hyperforin
ANOVA -log p-value = 33.00
Area
35000000
30000000
25000000
20000000
15000000
10000000
5000000
0
H.perforatum
H. maculatum
H. androsaemum
H. humifusum
H. bupleuroides
H. kalmianum
H. annulatum
H. balearicum
H. tomentosum
H. tetrapterum
H. pulchrum
H. erectum
H. stellatum
H. monogynum
H. kouytchense
H. canariense
H. rumeliacum

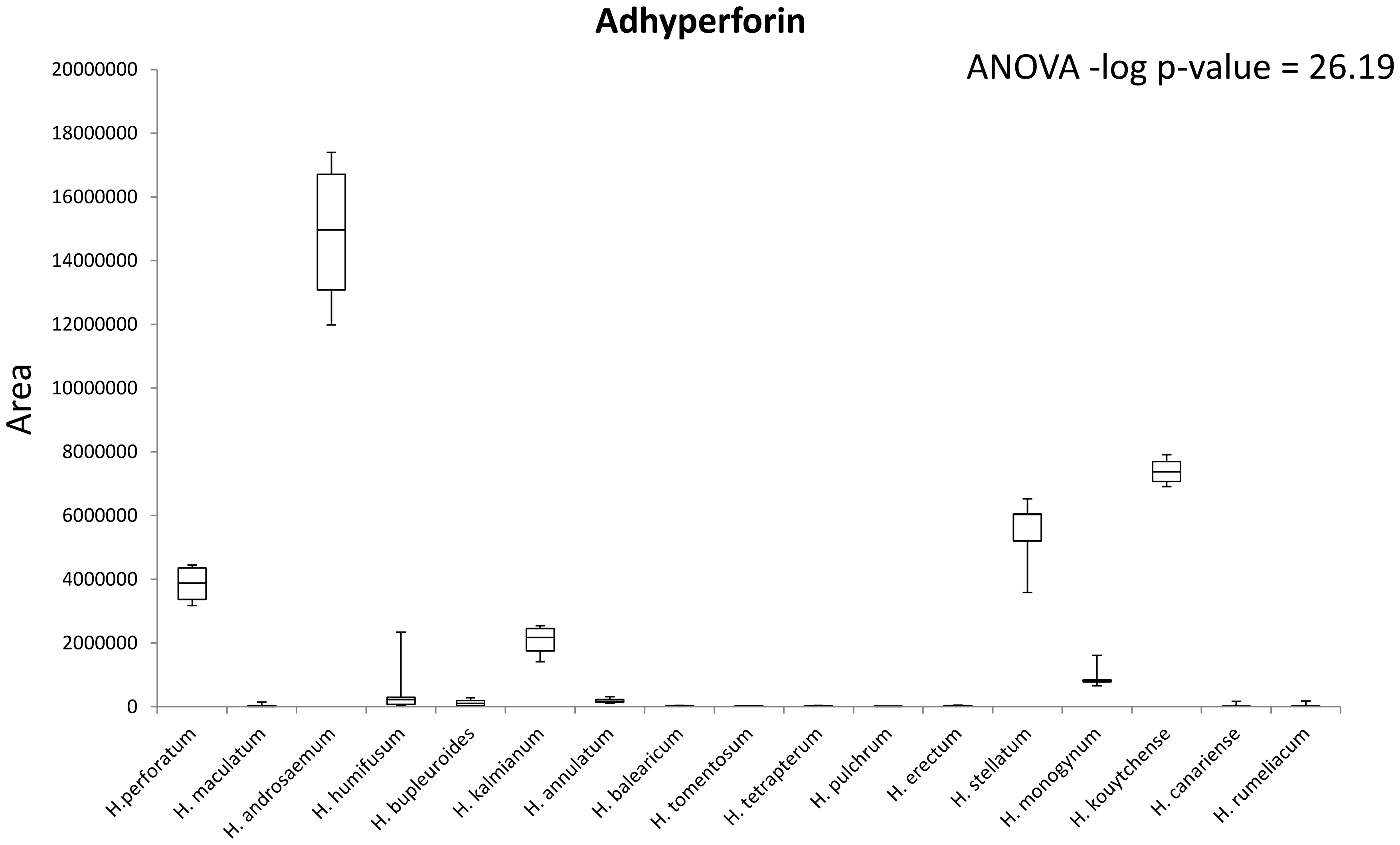
Adhyperforin
ANOVA -log p-value = 26.19
Area
20000000
18000000
16000000
14000000
12000000
10000000
8000000
6000000
4000000
2000000
0
H.perforatum
H. maculatum
H. androsaemum
H. humifusum
H. bupleuroides
H. kalmianum
H. annulatum
H. balearicum
H. tomentosum
H. tetrapterum
H. pulchrum
H. erectum
H. stellatum
H. monogynum
H. kouytchense
H. canariense
H. rumeliacum

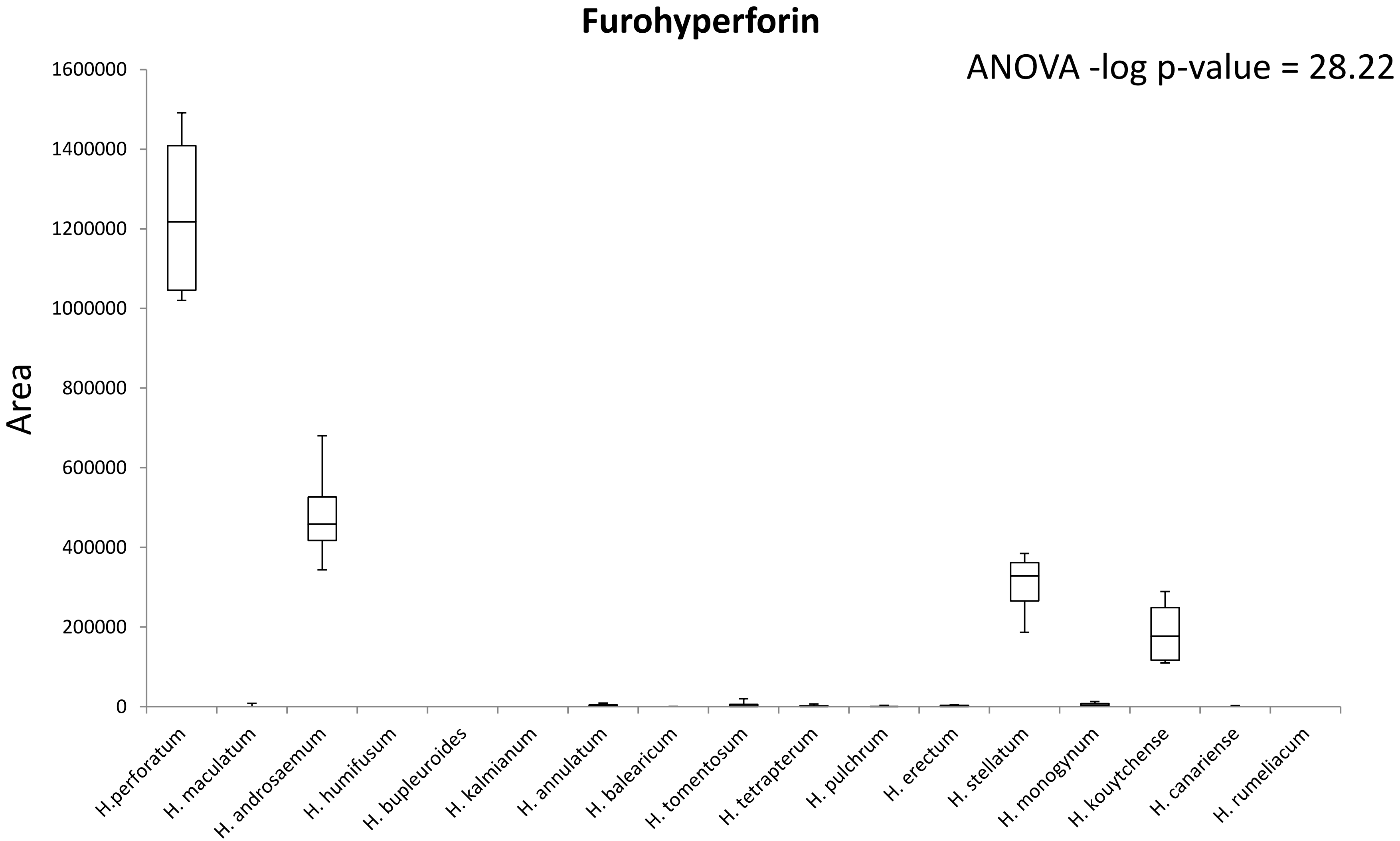
Furohyperforin
ANOVA -log p-value = 28.22
Area
1600000
1400000
1200000
1000000
800000
600000
400000
200000
0
H.perforatum
H. maculatum
H. androsaemum
H. humifusum
H. bupleuroides
H. kalmianum
H. annulatum
H. balearicum
H. tomentosum
H. tetrapterum
H. pulchrum
H. erectum
H. stellatum
H. monogynum
H. kouytchense
H. canariense
H. rumeliacum

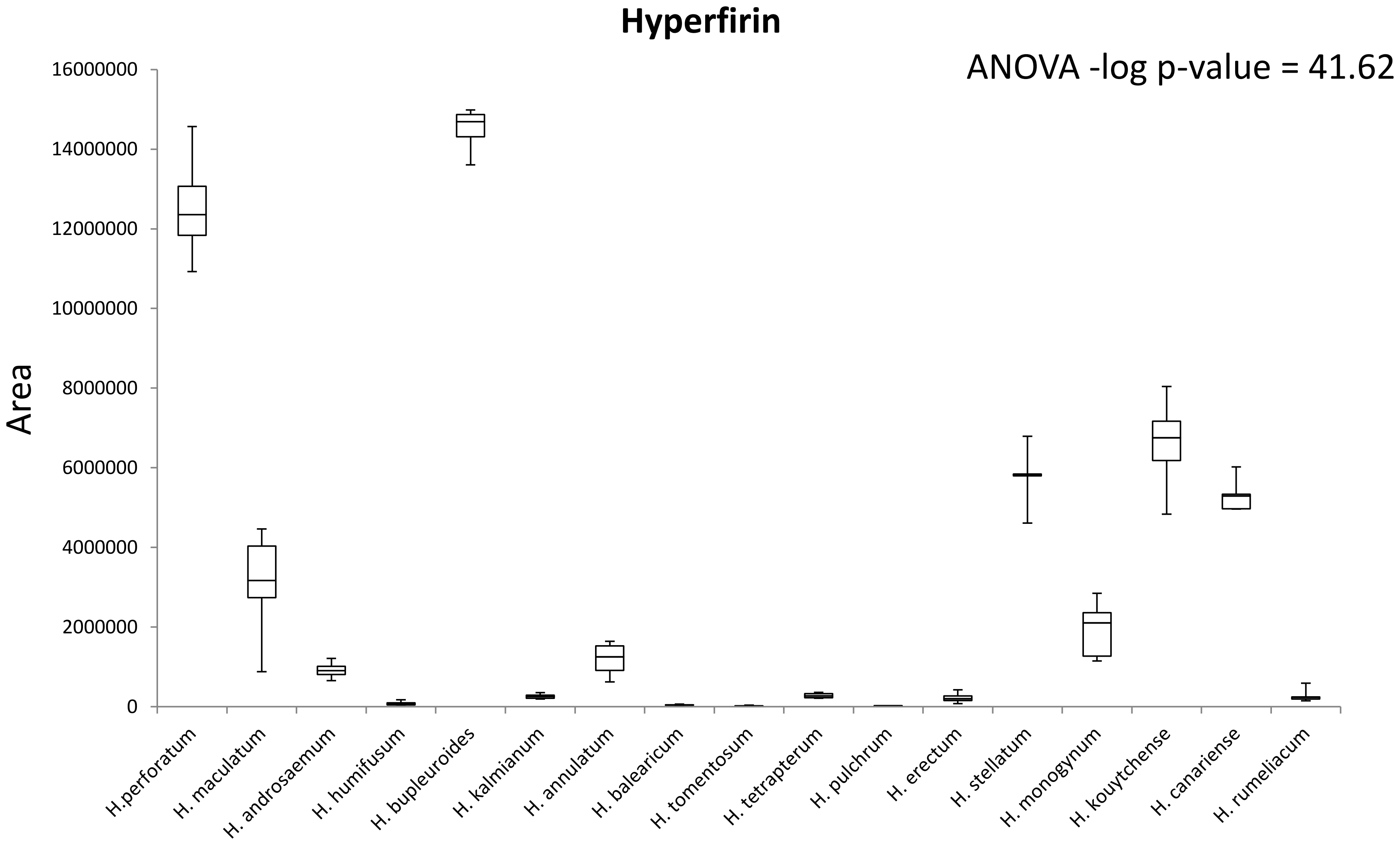
Hyperfirin
ANOVA -log p-value = 41.62
Area
16000000
14000000
12000000
10000000
8000000
6000000
4000000
2000000
0
H.perforatum
H. maculatum
H. androsaemum
H. humifusum
H. bupleuroides
H. kalmianum
H. annulatum
H. balearicum
H. tomentosum
H. tetrapterum
H. pulchrum
H. erectum
H. stellatum
H. monogynum
H. kouytchense
H. canariense
H. rumeliacum

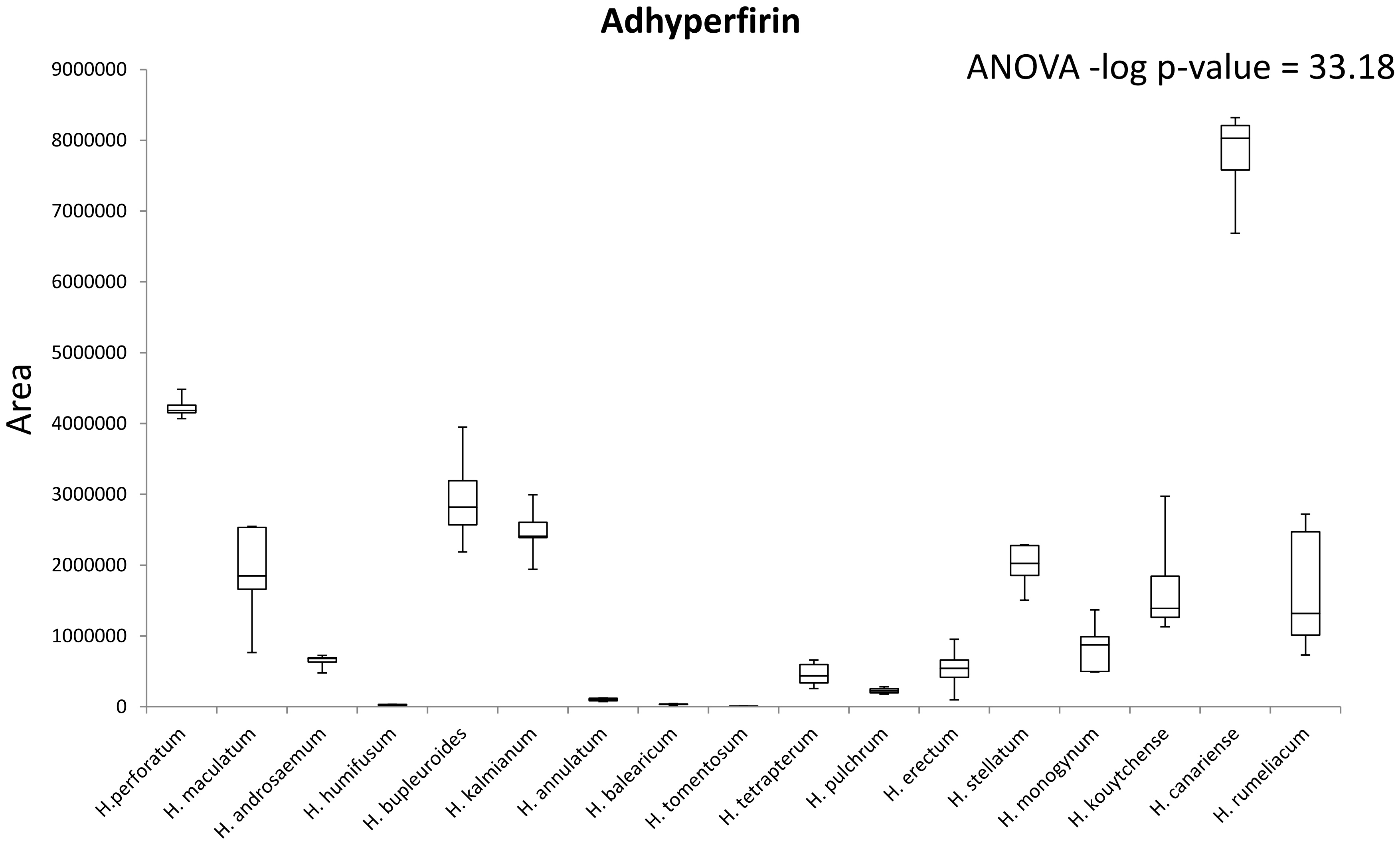
Adhyperfirin
ANOVA -log p-value = 33.18
Area
9000000
8000000
7000000
6000000
5000000
4000000
3000000
2000000
1000000
0
H.perforatum
H. maculatum
H. androsaemum
H. humifusum
H. bupleuroides
H. kalmianum
H. annulatum
H. balearicum
H. tomentosum
H. tetrapterum
H. pulchrum
H. erectum
H. stellatum
H. monogynum
H. kouytchense
H. canariense
H. rumeliacum

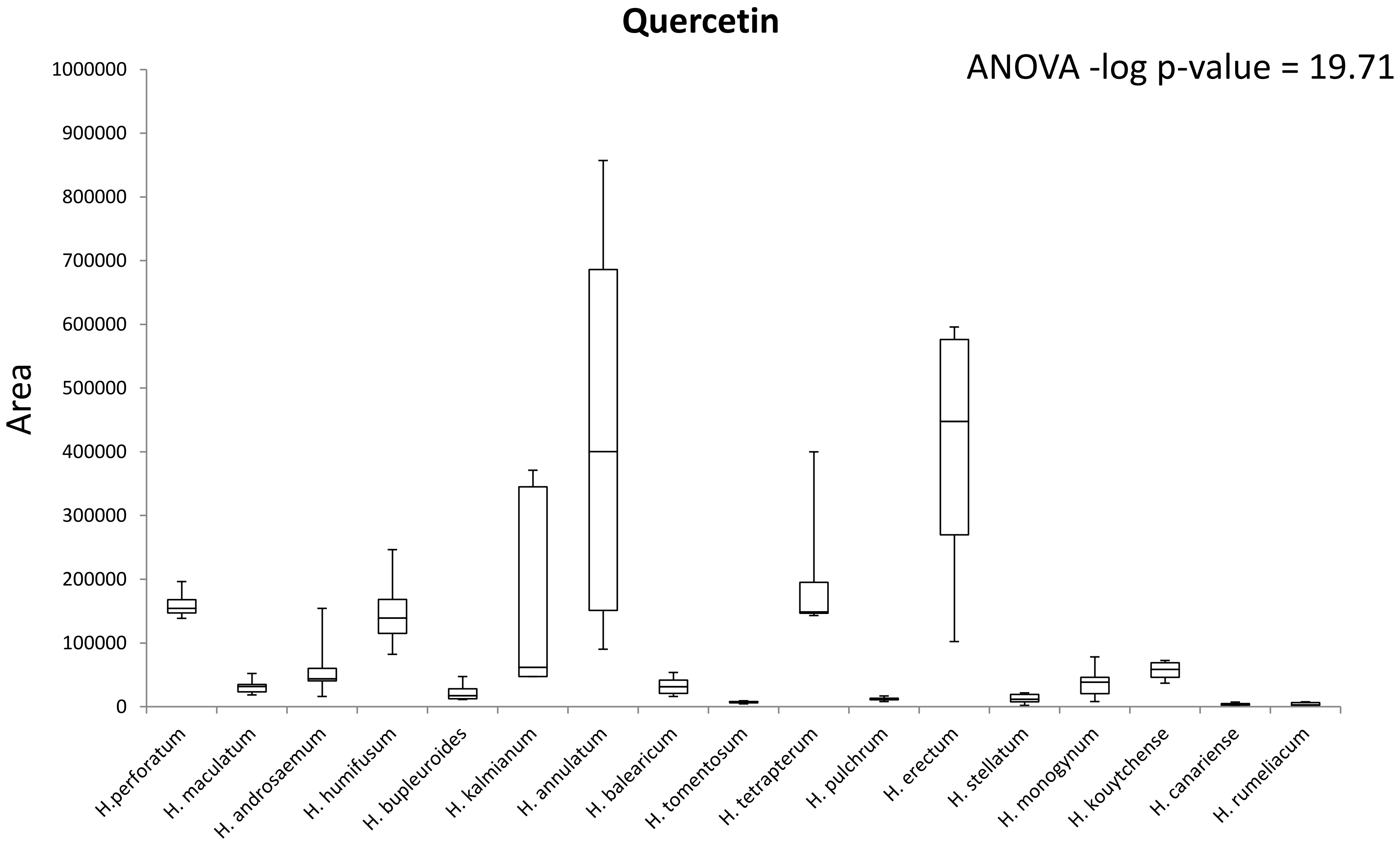
Quercetin
ANOVA -log p-value = 19.71
Area
1000000
900000
800000
700000
600000
500000
400000
300000
200000
100000
0
H.perforatum
H. maculatum
H. androsaemum
H. humifusum
H. bupleuroides
H. kalmianum
H. annulatum
H. balearicum
H. tomentosum
H. tetrapterum
H. pulchrum
H. erectum
H. stellatum
H. monogynum
H. kouytchense
H. canariense
H. rumeliacum

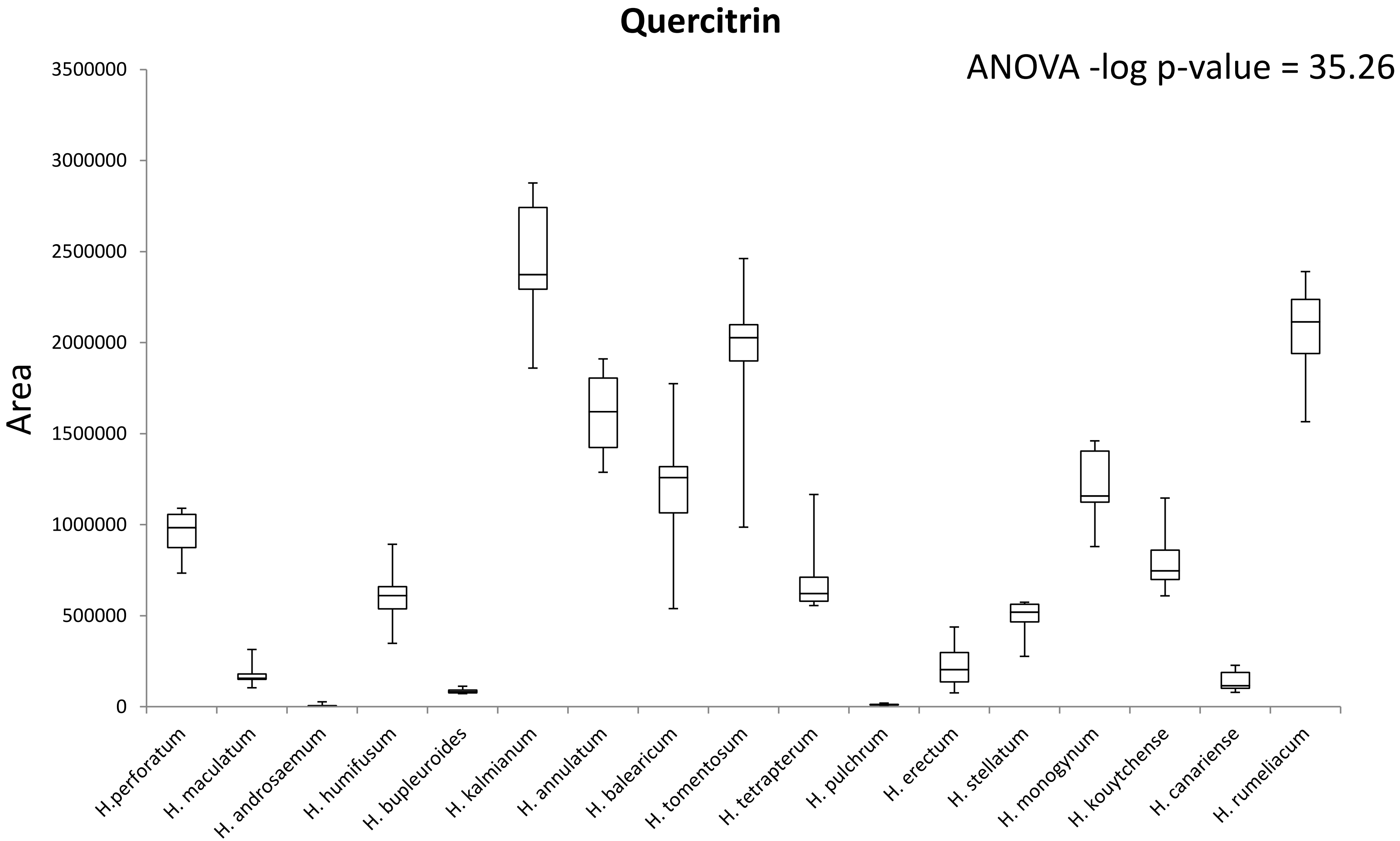
Quercitrin
ANOVA -log p-value = 35.26
Area
3500000
3000000
2500000
2000000
1500000
1000000
500000
0
H.perforatum
H. maculatum
H. androsaemum
H. humifusum
H. bupleuroides
H. kalmianum
H. annulatum
H. balearicum
H. tomentosum
H. tetrapterum
H. pulchrum
H. erectum
H. stellatum
H. monogynum
H. kouytchense
H. canariense
H. rumeliacum

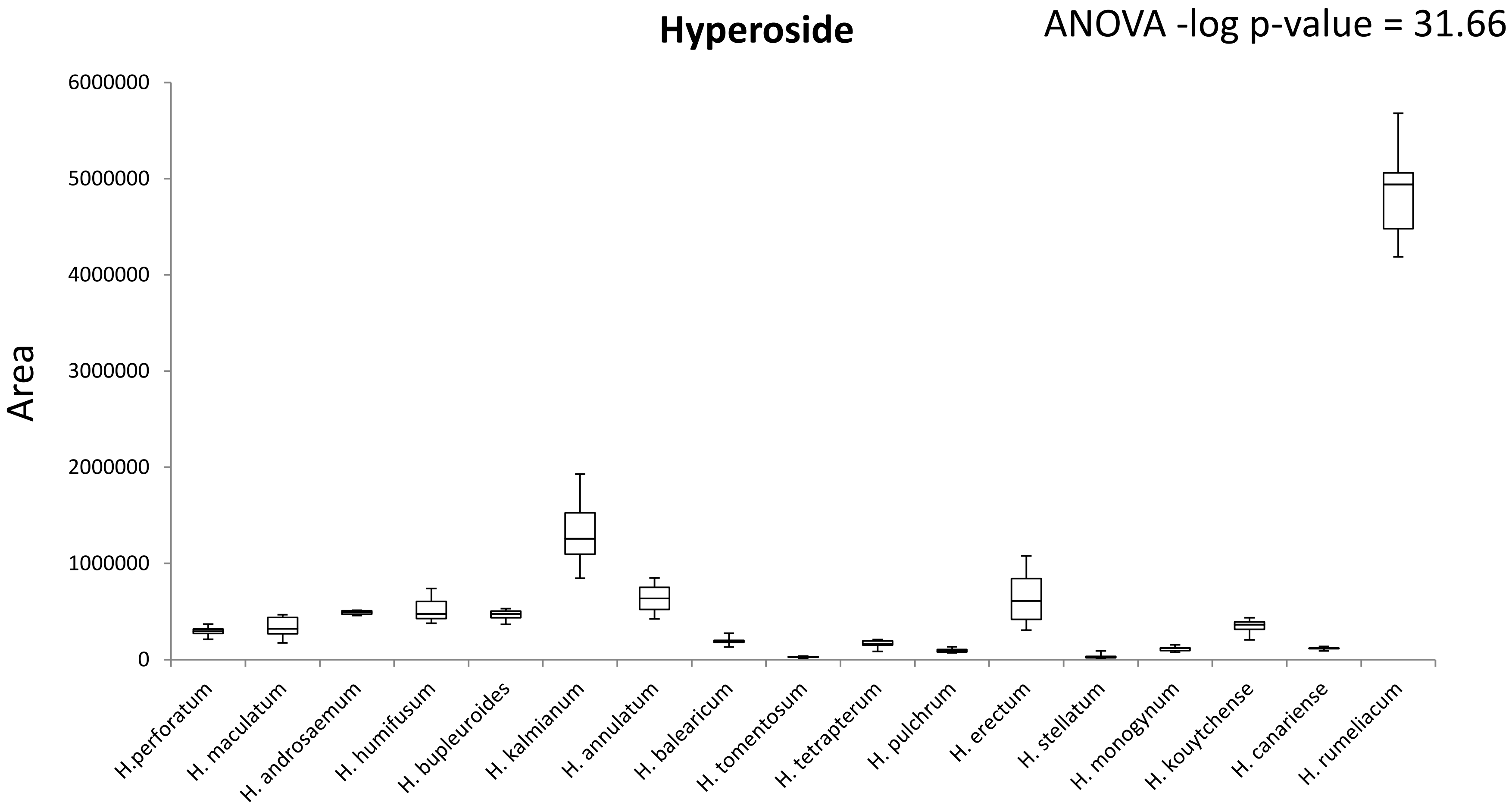
Hyperoside
ANOVA -log p-value = 31.66
Area
6000000
5000000
4000000
3000000
2000000
1000000
0
H.perforatum
H. maculatum
H. androsaemum
H. humifusum
H. bupleuroides
H. kalmianum
H. annulatum
H. balearicum
H. tomentosum
H. tetrapterum
H. pulchrum
H. erectum
H. stellatum
H. monogynum
H. kouytchense
H. canariense
H. rumeliacum

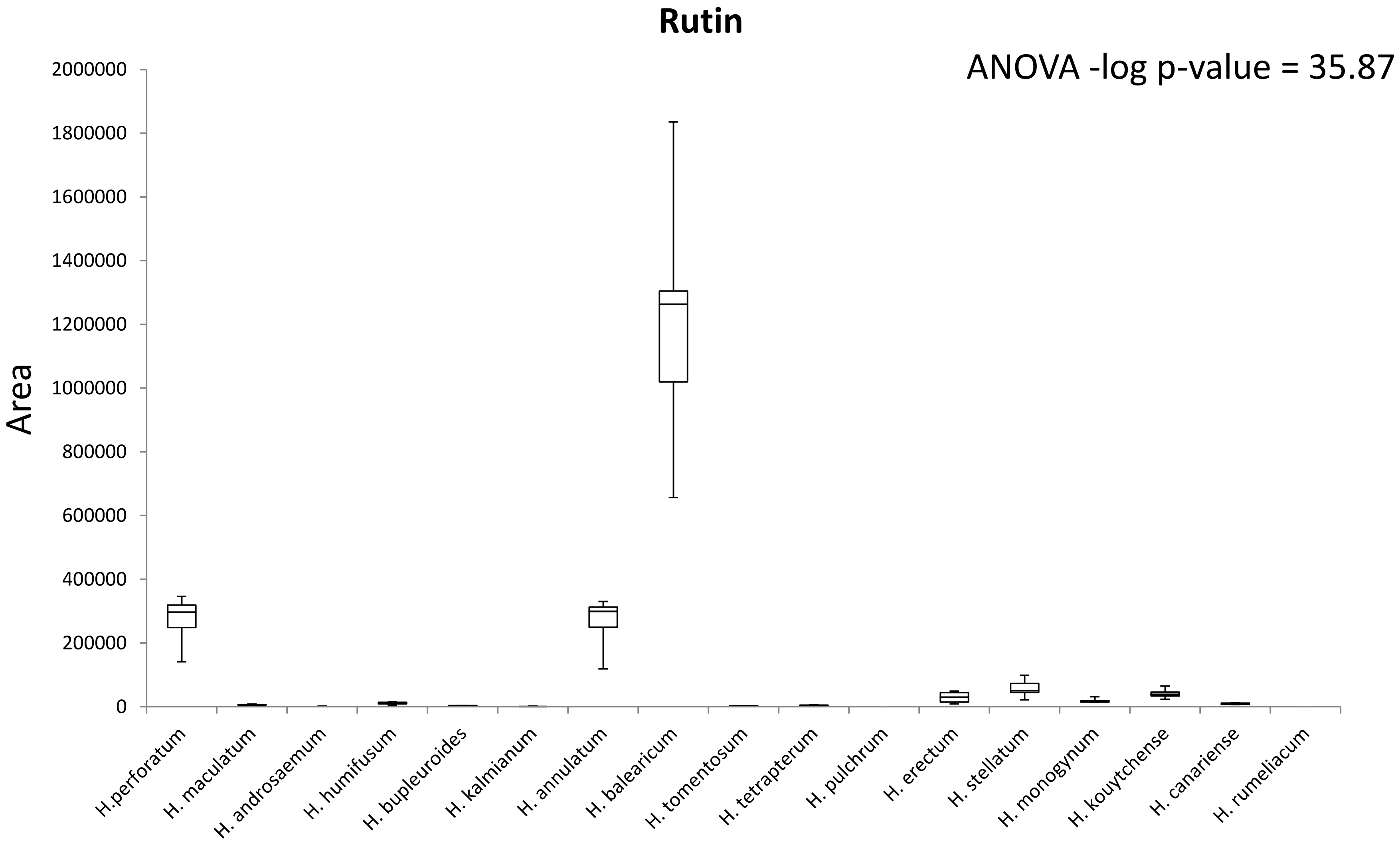
Rutin
ANOVA -log p-value = 35.87
Area
2000000
1800000
1600000
1400000
1200000
1000000
800000
600000
400000
200000
0
H.perforatum
H. maculatum
H. androsaemum
H. humifusum
H. bupleuroides
H. kalmianum
H. annulatum
H. balearicum
H. tomentosum
H. tetrapterum
H. pulchrum
H. erectum
H. stellatum
H. monogynum
H. kouytchense
H. canariense
H. rumeliacum

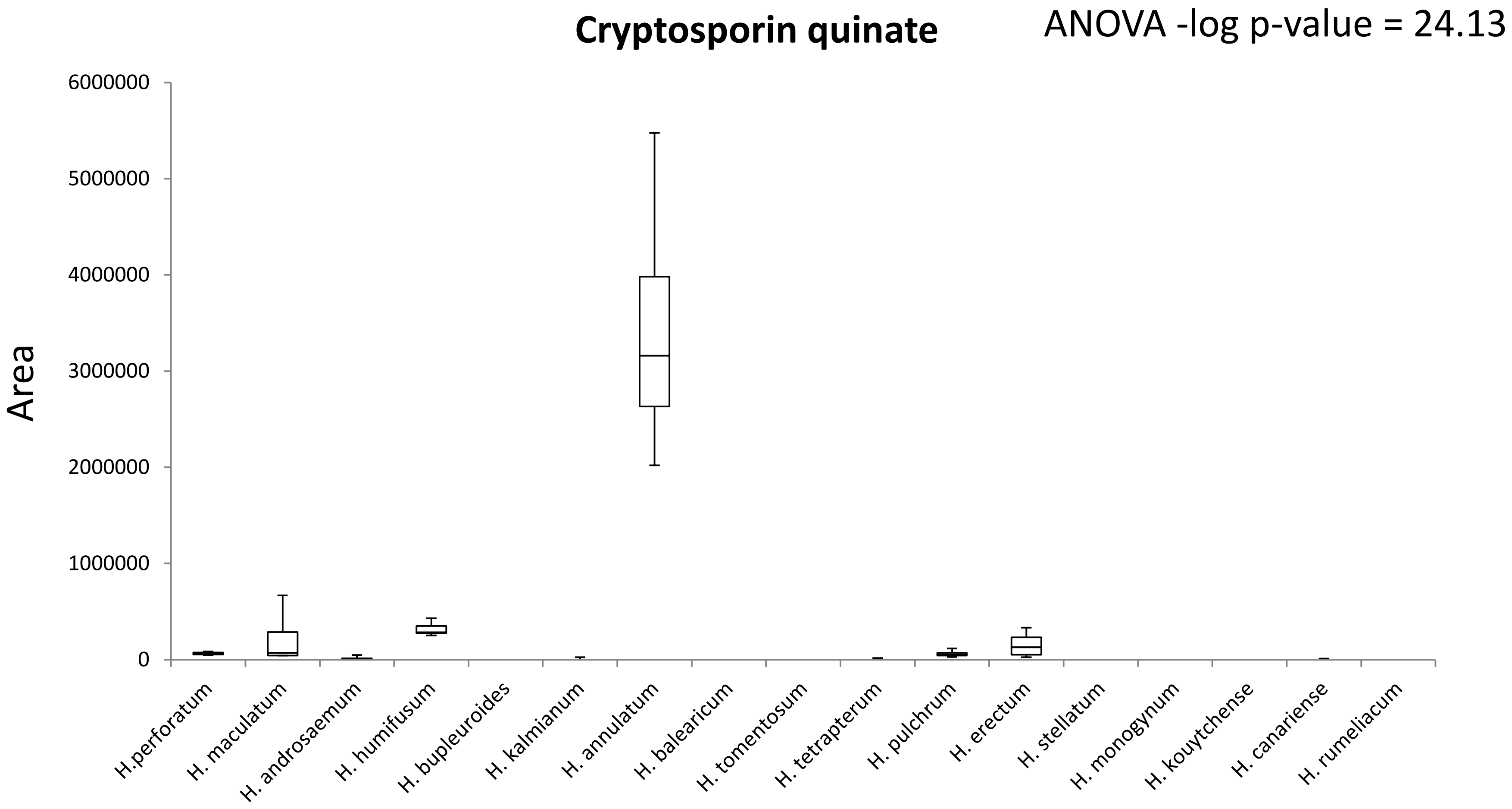
Cryptosporin quinate
ANOVA -log p-value = 24.13
Area
6000000
5000000
4000000
3000000
2000000
1000000
0
H.perforatum
H. maculatum
H. androsaemum
H. humifusum
H. bupleuroides
H. kalmianum
H. annulatum
H. balearicum
H. tomentosum
H. tetrapterum
H. pulchrum
H. erectum
H. stellatum
H. monogynum
H. kouytchense
H. canariense
H. rumeliacum

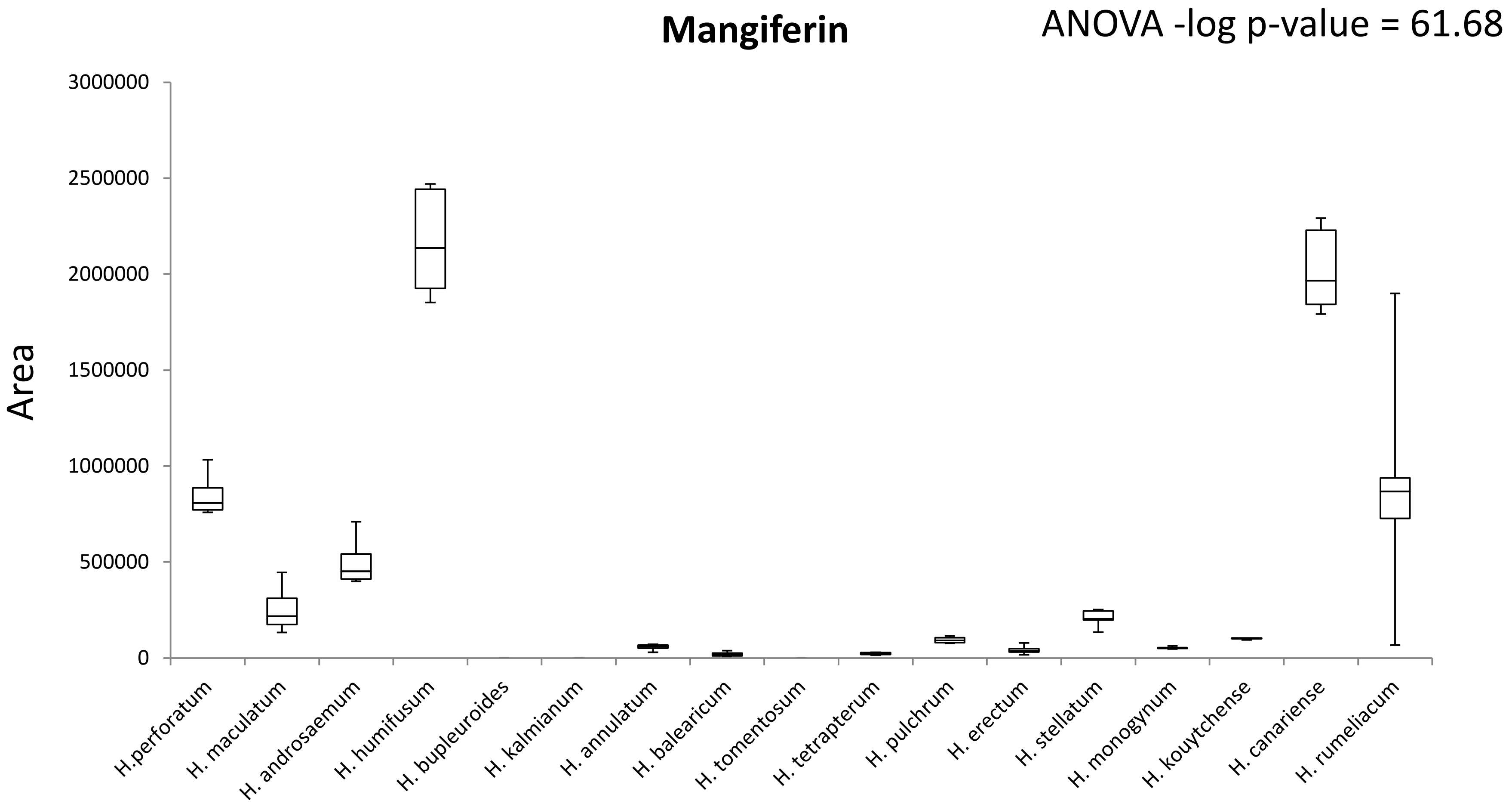
Mangiferin
ANOVA -log p-value = 61.68
Area
3000000
2500000
2000000
1500000
1000000
500000
0
H.perforatum
H. maculatum
H. androsaemum
H. humifusum
H. bupleuroides
H. kalmianum
H. annulatum
H. balearicum
H. tomentosum
H. tetrapterum
H. pulchrum
H. erectum
H. stellatum
H. monogynum
H. kouytchense
H. canariense
H. rumeliacum

4-Hydroxy-1-isobutyryl-8-methyl-3,7,8-tris(3-methyl-2-buten-1-yl)-5-(2-methyl-1-propen-1-yl)bicyclo[3.3.1]non-3-ene-2,9-dione (507.32 m/z)

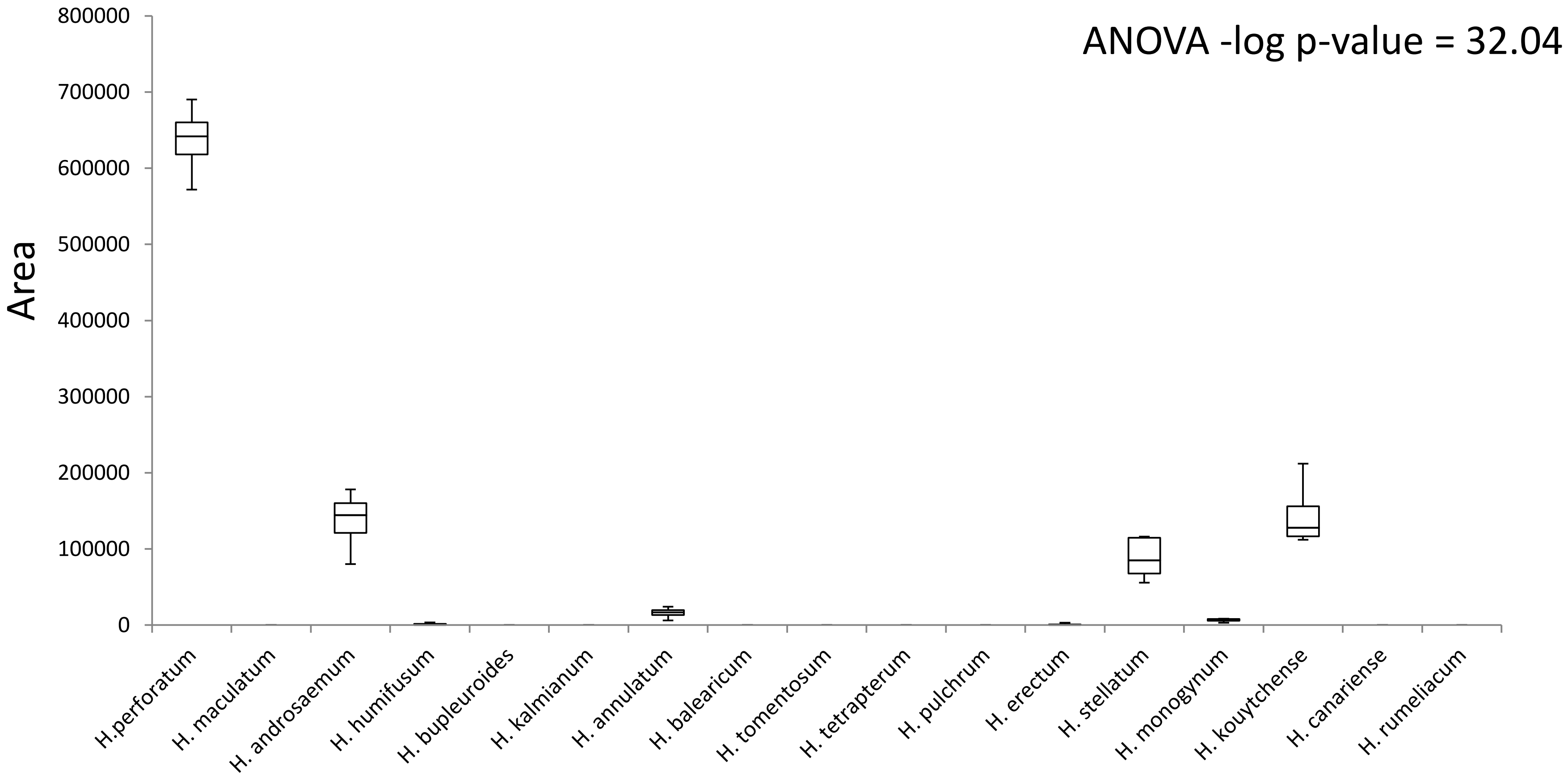

Fig. S3

a)

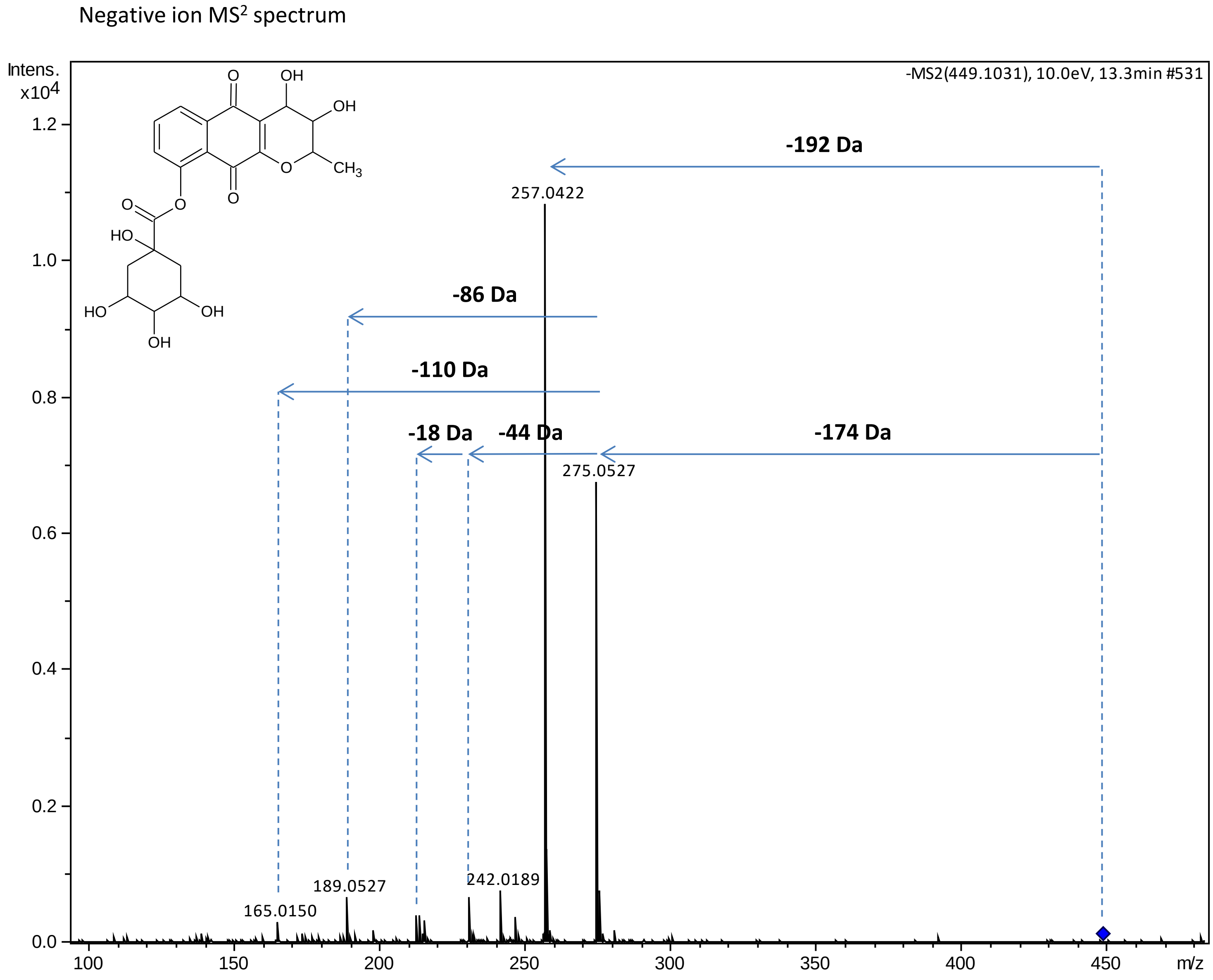
Negative ion MS2 spectrum
Intens. x10^4
-MS2(449.1031), 10.0eV, 13.3min #531
-192 Da
-86 Da
-110 Da
-18 Da
-44 Da
-174 Da
257.0422
275.0527
242.0189
189.0527
165.0150
m/z
OH
OH
CH3
HO
HO
OH
OH

b) Cryptosporin quinate fragmentation pathway of [M-H]$^-$

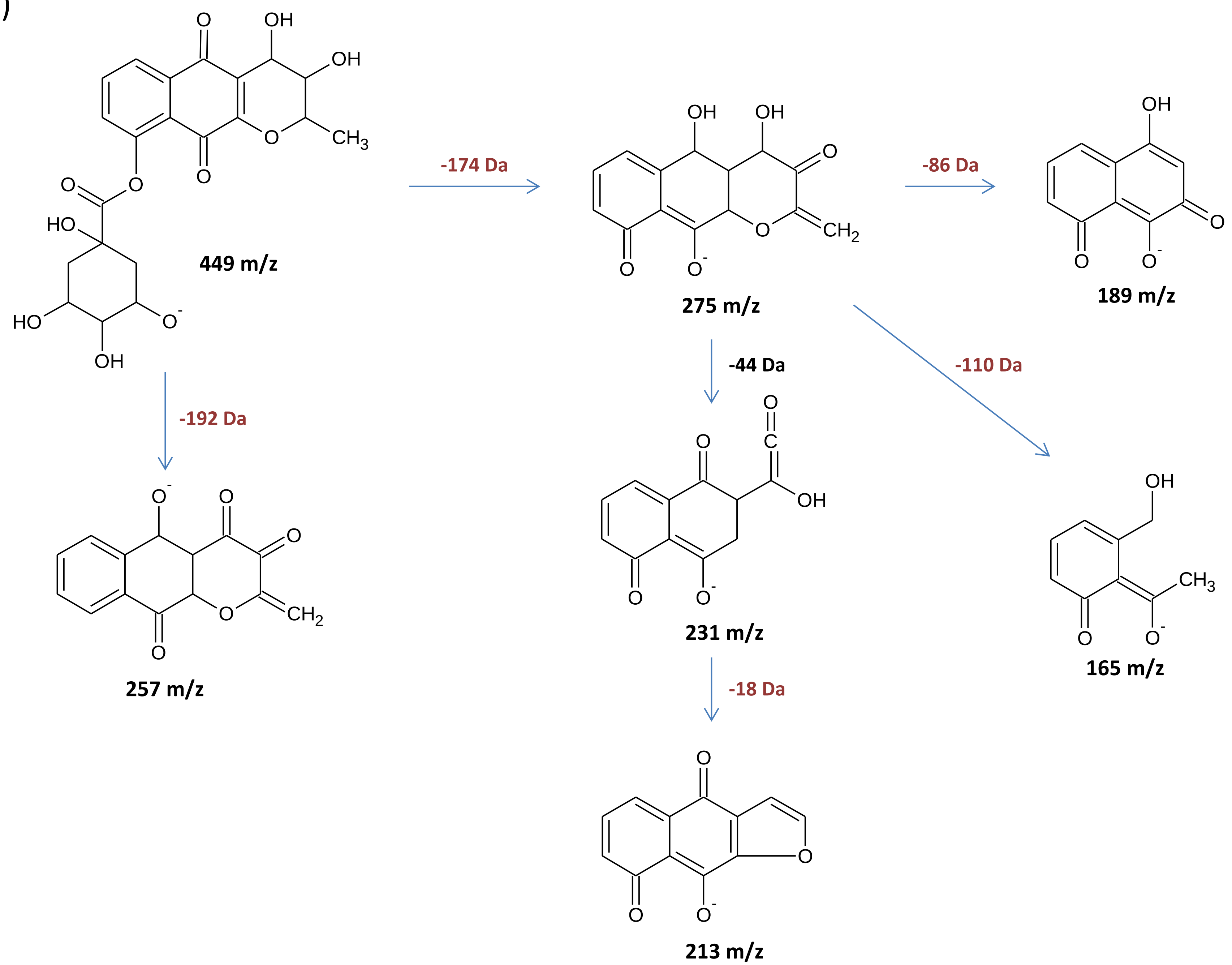

c)

Positive ion MS² spectrum

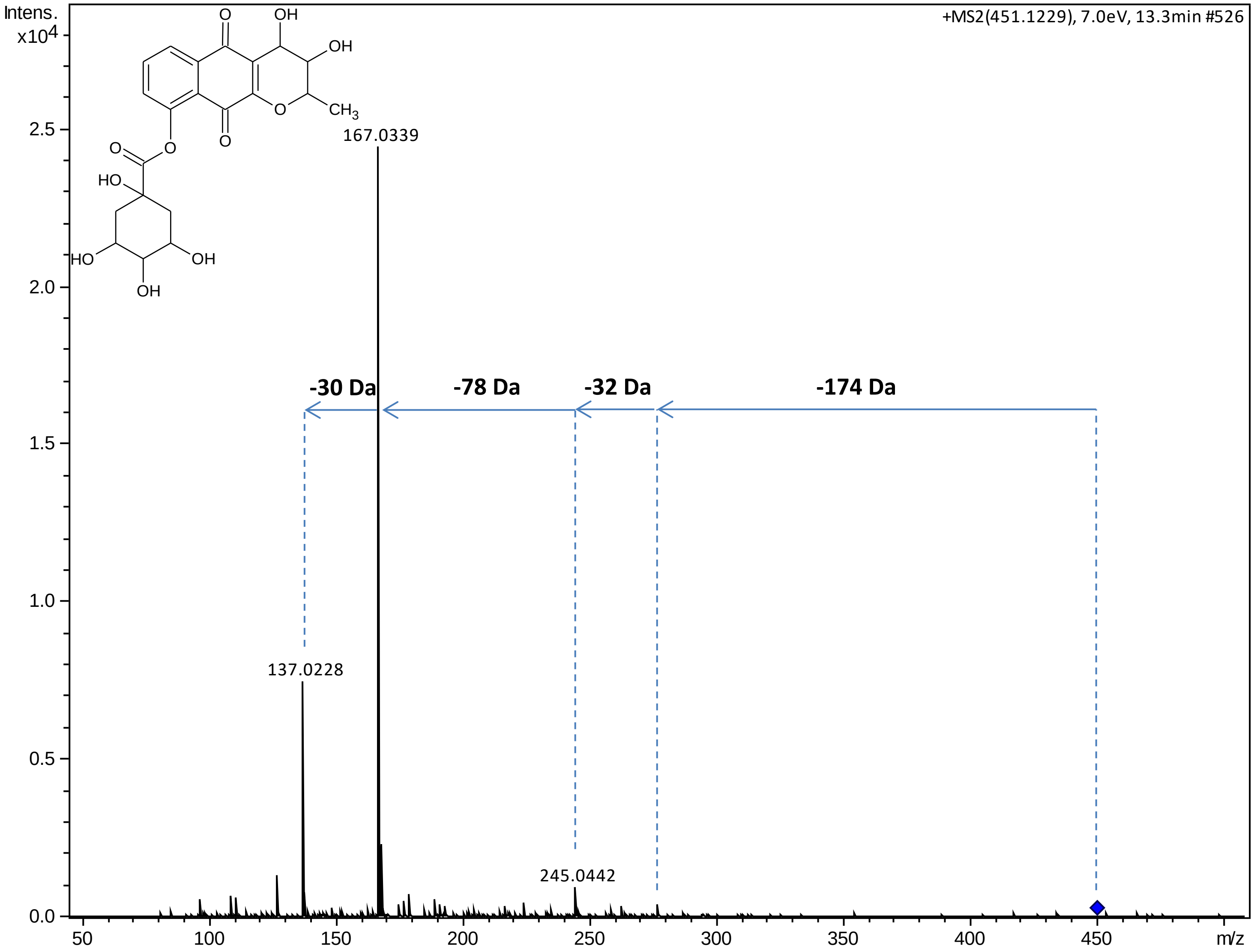

d) Cryptosporin quinate fragmentation pathway of [M+H]+

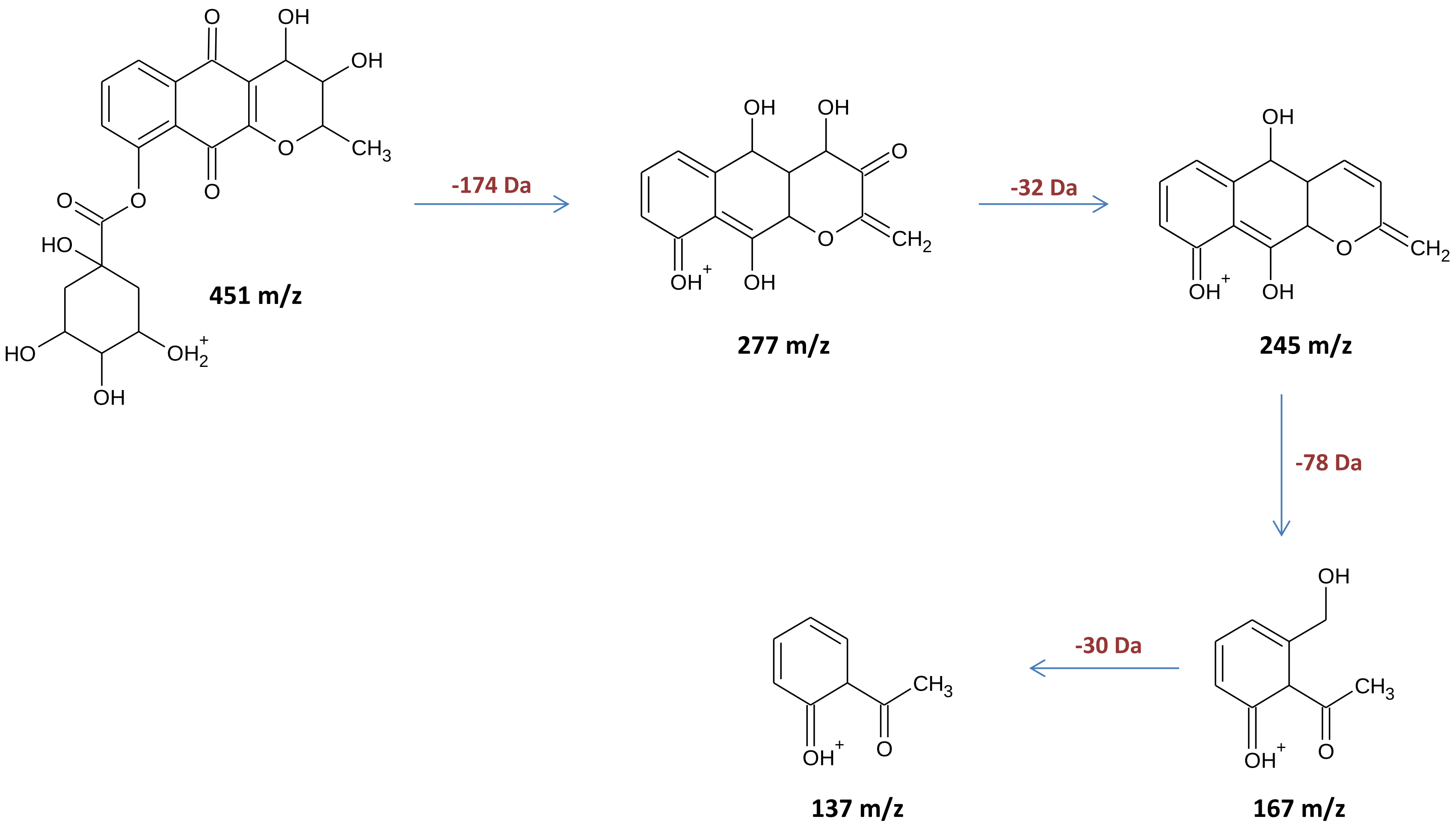

Fig. S3 Cryptosporin quinate (32) (a) structure and a negative ion MS2 spectrum, (b) fragmentation pathway in negative ion mode, (c) structure and a positive ion MS2 spectrum, and (d) fragmentation pathway in positive ion mode

4-Hydroxy-1-isobutyryl-8-methyl-3,7,8-tris(3-methyl-2-buten-1-yl)-5-(2-methyl-1-propen-1-yl)bicyclo[3.3.1]non-3-ene-2,9-dione (34 in Table 1)

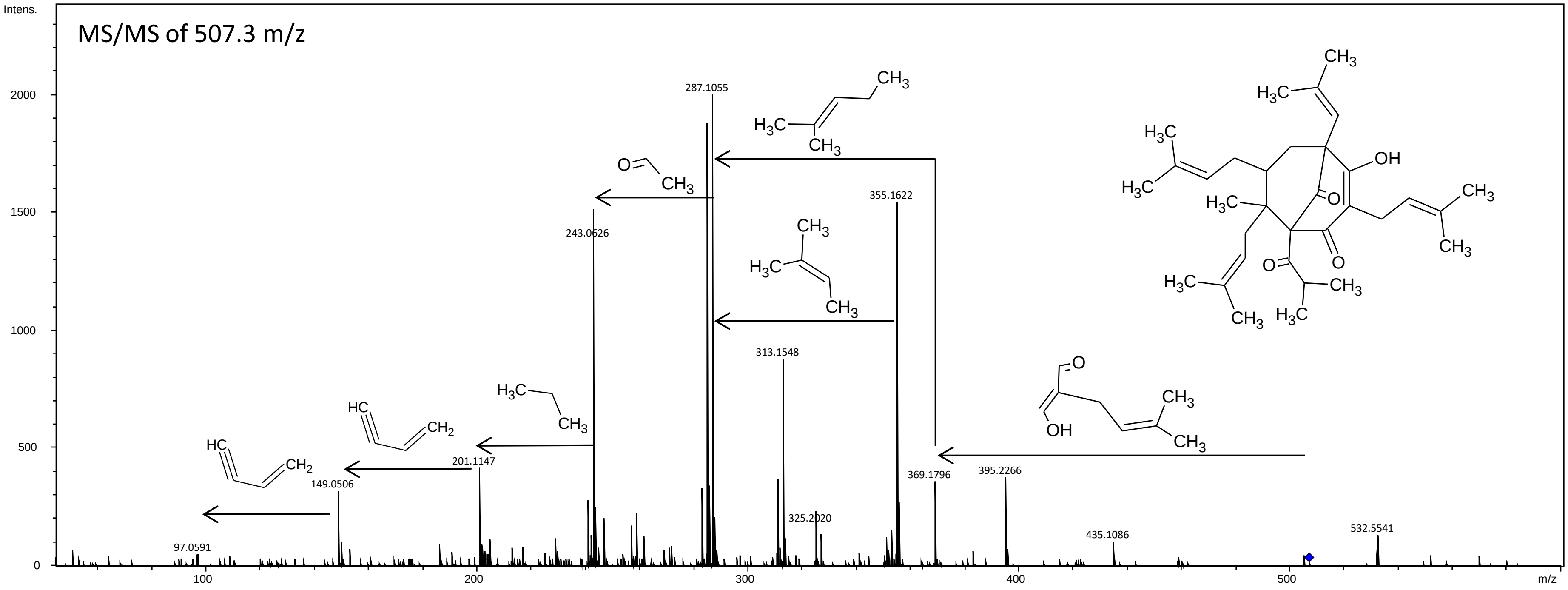

Fig. S4 Negative ion MS2 spectrum and supposed structure and fragmentation of a phloroglucinol derivative, 4-Hydroxy-1-isobutyryl-8-methyl-3,7,8-tris(3-methyl-2-buten-1-yl)-5-(2-methyl-1-propen-1-yl)bicyclo[3.3.1]non-3-ene-2,9-dione 34, with an $[M-H]^-$ at m/z 507.3479

Table S1 List of metabolites putatively identified in seventeen Hypericum spp. based on MS/MS spectra

No	Identified metabolite	Elemental composition [$C_nH_mO_x$]	[M-H]$^-$ [*m/z*] obtained (calculated, delta ppm)	Fragments obtained *m/z* (calculated mass, delta ppm)
1.	1,2,4,5-tetrahydroxy-7-(hydroxymethyl)-9,10-anthraquinone	$C_{15}H_{10}O_7$	**301.0349** (301.0353, 1.33)	**271. 0256** (271.0248, 2.9), **243.0318** (243.0298, 7.8)
2.	1,2,4,5-tetrahydroxy-7-methyl-9,10-anthraquinone-2-O-β-glucopyranoside	$C_{21}H_{20}O_{11}$	**447.0973** (447.0932, 9.17)	**285.0419** (285.0399, 5.0)
3.	skyrin-6-O-β-glucopyranoside	$C_{36}H_{28}O_{15}$	**699.1413** (699.1355, 8.30)	**519.0769** (519.0721, 9.1)
4.	Skyrin (2,2',4,4',5,5'-hexahydroxy-7,7'-dimethyl-1,1'-bianthracene-9,9',10,10'-tetrone)	$C_{30}H_{18}O_{10}$	**537.0869** (537.0827, 7.82)	**509.0897** (509.0878, 3.7), **467.0791** (467.0772, 4.0)
9.	Emodin (1,3,8-trihydroxy-6-methylanthracene-9,10-dione)	$C_{15}H_{10}O_5$	**269.0459** (269.0455, 1.49)	**240.0439** (240.0428, 4.6), **225.0567** (225.0557, 4.4), **197.0621** (197.0608, 6.6)
10.	Emodin anthrone (1,3,8-trihydroxy-6-methyl-10H-anthracen-9-one)	$C_{15}H_{12}O_4$	**255.0629** (255.0662, 12.94)	**237.0570** (237.0551, 5.4), **213.0568** (213.0551, 5.1)
12.	caffeoylquinic acid isomer – CQA I[a]	$C_{16}H_{18}O_9$	**353.0872** (353.0878, 1.70)	**191.0545** (191.0556, 8.4)
13.	caffeoylquinic acid isomer – CQA II[a]	$C_{16}H_{18}O_9$	**353.0877** (353.0878, 0.28)	**191.0549** (191.0556, 6.3)
14.	caffeoylquinic acid isomer – CQA III[a]	$C_{16}H_{18}O_9$	**353.0865** (353.0878, 3.68)	**191.0551** (191.0556, 5.3)
15.	dicaffeoylquinic acid isomer – diCQA I[a]	$C_{25}H_{24}O_{12}$	**515.1204** (515.1195, 1.75)	**191.0544** (191.0556, 9.0)
16.	dicaffeoylquinic acid isomer – diCQA II[a]	$C_{25}H_{24}O_{12}$	**515.1211** (515.1195, 3.11)	**191.0545** (191.0556, 8.4)
17.	feruoylquinic acid isomer– FerQA I[a]	$C_{17}H_{20}O_9$	**367.1044** (367.1035, 2.45)	**193.0511** (193.0500, 2.4), **191.0572** (191.0556, 5.7)
18.	feruoylquinic acid isomer – FerQA II[a]	$C_{17}H_{20}O_9$	**367.1045** (367.1035, 2.45)	**193.0518** (193.0500, 6.0), **191.0571** (191.0556, 5.2)
19.	coumaroylquinic acid isomer – CouQA I[a]	$C_{16}H_{18}O_8$	**337.0934** (337.0929, 1.48)	**191.0544** (191.0556, 9.0)
20.	coumaroylquinic acid isomer – CouQA II[a]	$C_{16}H_{18}O_8$	**337.0939** (337.0929, 2.97)	**191.0552** (191.0556, 4.8)
21.	coumaroylquinic acid isomer – CouQA III[a]	$C_{16}H_{18}O_8$	**337.0923** (337.0929, 1.78)	**191.0550** (191.0556, 5.8)
22.	coumaroylquinic acid isomer – CouQA IV[a]	$C_{16}H_{18}O_8$	**337.0939** (337.0929, 2.97)	**191.0548** (191.0556, 6.9)
24.	Adhyperforin (6-methyl-5-(2-methylbutanoyl)-1,3,7-tris(3-methylbut-2-enyl)-6-(4-methylpent-3-enyl)-4,9-dioxobicyclo[3.3.1]non-2-en-2-olate)	$C_{36}H_{54}O_4$	**549.3934** (549.3949, 2.73)	**397.2761** (397.2748, 3.2), **327.1979** (327.1965, 4.1)
25.	Furohyperforin ((1S,3S,8R,9R,10S)-3-(2-Hydroxy-2-propanyl)-8-isobutyryl-9-methyl-6,10-bis(3-methyl-2-buten-1-yl)-9-(4-methyl-3-penten-1-yl)-4-oxatricyclo[6.3.1.01,5]dodec-5-ene-7,12-dione)	$C_{35}H_{52}O_5$	**551.3725** (551.3742, 3.08)	**481.2978** (481.2959, 3.8)
26.	Hyperfirin (8S)-4-Hydroxy-1-isobutyryl-8-methyl-3,5-bis(3-methyl-2-buten-1-yl)-8-(4-methyl-3-penten-1-yl)bicyclo[3.3.1]non-3-ene-2,9-dione)	$C_{30}H_{44}O_4$	**467.3164** (467.3167, 0.63)	**397.2764** (397.2748, 4.0)
27.	Adhyperfirin ((1R,5R,6S)-2-hydroxy-6-methyl-5-(2-methylbutanoyl)-1,3-bis(3-methylbut-2-enyl)-6-(4-methylpent-3-enyl)bicyclo[3.3.1]non-2-ene-4,9-dione)	$C_{31}H_{46}O_4$	**481.3311** (481.3323, 2.49)	**413.2719** (413.2697, 5.2)
29.	Quercitrin (Quercetin 3-rhamnoside)	$C_{21}H_{20}O_{11}$	**447.0973** (447.0932, 9.17)	**301.0374** (301.0353, 6.7)
30.	Hyperoside (Quercetin 3-*O*-galactoside)	$C_{21}H_{20}O_{12}$	**463.0908** (463.0882, 5.61)	**301.0377** (301.0353, 7.7)
31.	Rutin (Quercetin 3-*O*-glucosyl-rhamnoside)	$C_{27}H_{30}O_{16}$	**609.1507** (609.1461, 7.55)	**301.0369** (301.0353, 5.1)
32.	Cryptosporin quinate	$C_{21}H_{22}O_{11}$	**449.1073** (449.1089, 3.56)	**275.0527** (275.0561, 12.4), **257.0422** (257.0455, 13.0)
33.	Mangiferin	$C_{19}H_{18}O_{11}$	**421.0771** (421.0776, 1.19)	**301.0379** (301.0353, 8.4), **331.0492** (331.0459, 9.8)
34.	4-Hydroxy-1-isobutyryl-8-methyl-3,7,8-tris(3-methyl-2-buten-1-yl)-5-(2-methyl-1-propen-1-yl)bicyclo[3.3.1]non-3-ene-2,9-dione	$C_{33}H_{48}O_4$	**507.3516** (507.3479, 7.29)	**369.1796** (369.1799, 0.8), **287.1055** (287.1016, 13.4)
